# Stringent response regulators (p)ppGpp and DksA positively regulate virulence and host adaptation of *Xanthomonas citri*

**DOI:** 10.1111/mpp.12865

**Published:** 2019-10-17

**Authors:** Yanan Zhang, Doron Teper, Jin Xu, Nian Wang

**Affiliations:** ^1^ Citrus Research and Education Center Department of Microbiology and Cell Science Institute of Food and Agricultural Sciences, University of Florida Lake Alfred 33850 FL United States

**Keywords:** citrus, host adaptation, RNA‐seq, stringent response, type 3 secretion system, *Xanthomonas citri*

## Abstract

The bacterial stringent response is a response to nutrition deprivation and other stress conditions. In Gram‐negative bacteria, this process is mediated by the small signal molecules guanosine pentaphosphate pppGpp and guanosine tetraphosphate ppGpp (collectively referred to as (p)ppGpp), and the RNA polymerase‐binding transcription factor DksA. The (p)ppGpp synthetase RelA and the bifunctional (p)ppGpp synthase/hydrolase SpoT are responsible for cellular (p)ppGpp levels. Here, we investigated the roles of DksA and (p)ppGpp in the virulence traits of *Xanthomonas citri* subsp. *citri* (Xcc), the causal agent of citrus canker. Δ*dksA* and (p)ppGpp‐deficient Δ*spoT*Δ*relA* strains caused reduced virulence and compromised growth in host plants, indicating that DksA and (p)ppGpp are required for full virulence of Xcc. To characterize the effect of stringent response regulators on gene expression, RNA‐seq was conducted using Δ*dksA* and Δ*spoT*Δ*relA* mutant strains grown in *hrp*‐inducing XVM2 medium. Transcriptome analyses showed that DksA and (p)ppGpp repressed the expression of genes encoding tRNAs, ribosome proteins, iron acquisition and flagellum assembly, and enhanced the expression of genes for histidine metabolism, type 3 secretion system (T3SS), type 2 secretion system (T2SS) and TonB‐dependent transporters. Phenotypically, the Δ*dksA* and Δ*spoT*Δ*relA* strains displayed altered motility, enhanced siderophore production and were unable to cause the hypersensitive response on non‐host plants. In conclusion, stringent response regulators DksA and (p)ppGpp play an important role in virulence, nutrition uptake and host adaptation of Xcc.

## Introduction

*Xanthomonas* spp. cause diseases in approximately 400 species of plant hosts, including many economically important crops (Ryan *et al.*, 2011). *Xanthomonas citri* subsp. *citri* (Xcc) is the causal agent of citrus canker, one of the most destructive bacterial diseases in citrus (Vojnov *et al.*, 2010). Xcc can be disseminated via wind‐driven rain and invades the host leaf mesophyll tissue through stomata or wounds (Brunings and Gabriel, 2003; Ference *et al.*, 2017). To survive and multiply, Xcc needs to overcome the stress from both the non‐host and host environments such as nutrient limitations in the phyllosphere (Fatima and Senthil‐Kumar, 2015) and the host immunity response (Dodds and Rathjen, 2010).

Gram‐negative bacterial pathogens, including Xcc, deliver numerous effectors into the host cell via the type 3 secretion system (T3SS) to manipulate host signalling, suppress immune responses and/or induce plant susceptibility genes (Jacques *et al.*, 2016; Tsuge *et al.*, 2014; White *et al.*, 2009). The T3SS is encoded by *hrp* (hypersensitive response and pathogenicity) genes and the regulation of *hrp* genes in *Xanthomonas* mainly depends on two key transcriptional regulators, HrpG and HrpX (Wengelnik and Bonas, 1996; Wengelnik *et al.*, 1996). HrpG positively regulates the expression of HrpX, which in turn binds to a conserved motif (PIP box, plant‐inducible promoter) at promoter regions of some T3SS genes, effector genes and other virulence‐related genes (Guo *et al.*, 2011; Koebnik *et al.*, 2006). Several virulence regulators were found to act upstream of HrpG and/or HrpX in *Xanthomonas*, including post‐transcriptional regulator RsmA/CsrA (Andrade *et al.*, 2014), Lon protease (Zhou *et al.*, 2018), sensor kinase HpaS (Li *et al.*, 2014), LysR‐type transcriptional activator GamR (Rashid *et al.*, 2016) and small noncoding RNA sX13 (Schmidtke *et al*., 2013). In addition, the virulence of *Xanthomonas* is also controlled by two‐component systems, quorum sensing (QS) and cyclic di‐GMP (Büttner and Bonas, 2010). Beyond the T3SS induction during infection, little is known about how Xcc coordinates virulence‐associated regulatory networks.

The bacterial stringent response is a response to nutrition starvation and other stress conditions. During the stringent response, cellular resources are relocated from the synthesis of stable RNAs (tRNA and rRNA) and ribosomal proteins to promote the expression of components crucial for stress resistance (Dalebroux and Swanson, 2012; Potrykus and Cashel, 2008). This process is mainly regulated by the small signal molecules guanosine pentaphosphate pppGpp and guanosine tetraphosphate ppGpp (collectively referred to as (p)ppGpp for simplicity), and the RNA polymerase‐binding transcription factor DksA (Haugen *et al.*, 2008). DksA, belonging to the DksA/TraR superfamily, binds to RNA polymerase (RNAP) through the secondary channel and augments the effect of (p)ppGpp on transcription initiation (Blankschien *et al*., 2009; Paul *et al.*, 2004; Perederina *et al.*, 2004). The cellular (p)ppGpp level is controlled by protein family RelA‐SpoT homologue (RSH), small alarmone synthetase (SAS) and small alarmone hydrolase (SAH) (Atkinson *et al.*, 2011). Gram‐negative bacteria generally have two long‐RSH proteins: RelA (synthetase) and SpoT (synthase and hydrolase) (Irving and Corrigan, 2018). (p)ppGpp can be synthesized by both RelA and SpoT using GTP/GDP and ATP and degraded only by SpoT, but not by RelA, into GTP/GDP and pyrophosphate (PPi) due to the loss of hydrolytic activity in the RelA (p)ppGpp hydrolysis (HD) domain (Hauryliuk *et al.*, 2015). The activity of RSH homologues in *Escherichia coli* could be regulated by small ligand, heterologous protein or at the transcriptional level (Irving and Corrigan, 2018).

Although the specific mechanism may vary between species, there are at least two paradigms for the broad effect of (p)ppGpp on cellular processes (Gourse *et al.*, 2018). In *E. coli*, both (p)ppGpp and DksA can directly bind to RNAP and this interaction positively or negatively regulates the transcription initiation depending on the kinetics of promoter open complex (Haugen *et al.*, 2008; Ross *et al.*, 2016). In addition, (p)ppGpp also regulates the global cellular process in a RNAP‐independent manner by directly interacting with enzymes or transcription factors and therefore modulating their activities (Dalebroux and Swanson, 2012).

Transcriptome analysis showed that in *E. coli* the stringent response regulates more than 10% of all genes involved in synthesis of tRNA, ribosome proteins, flagella, fatty acids, amino acids, transporters and many other central cellular components (Aberg *et al*., 2009; Durfee *et al.*, 2008; Traxler *et al.*, 2008). Although the synergistic effect between DksA and (p)ppGpp exists in most cases, there is evidence showing that they may have divergent and even opposite effects on specific traits or gene expression (Lyzen *et al.*, 2009, 2016; Magnusson *et al.*, 2007). It is notable that even the basal level of (p)ppGpp still has a regulatory effect under balanced growth conditions (Gaca *et al.*, 2013).

A stringent response not only helps bacteria adapt to a nutrient‐deprived environment, but also enhances bacterial virulence (Dalebroux *et al.*, 2010). For plant pathogens, several reports showed the effect of stringent response regulators (p)ppGpp and/or DksA on virulence in plant pathogens like *Erwinia amylovora* (Ancona *et al.*, 2015), *Pectobacterium atrosepticum* (Bowden *et al.*, 2013) and *Pseudomonas syringae* (Chatnaparat *et al.*, 2015a,b). The effect of stringent response regulators on virulence has yet to be studied in *Xanthomonas* genus. In this study, we examined stringent response regulators by generating Δ*dksA* and (p)ppGpp‐deficient Δ*spoT*Δ*relA* strains of Xcc. Using whole transcriptome analysis, virulence tests and phenotypic characterization, our study provides new insights into the functions of stringent response regulators as well as the positive and negative interplay between (p)ppGpp and DksA in *Xanthomonas* species.

## Results

### DksA and SpoT are required for the full pathogenicity of Xcc

A homology search showed that Xcc contains stringent response regulatory genes including *dksA* (locus tag XAC2358), *relA* (XAC3113) and *spoT* (XAC3393). BLASTP showed that DksA, RelA and SpoT from Xcc displayed 46.88%, 43.00% and 48.06% identity in amino acid sequence, respectively, with the homologous genes from *E. coli* K‐12 MG1655*.* DksA belongs to the DksA/TraR family and contains a zinc finger domain at the C‐terminal region, a typical feature of DksA/TraR family members (Blankschien *et al*., 2009) (Fig. S1). Both RelA and SpoT contain a (p)ppGpp synthesis (SYNTH) domain, a hydrolysis (HD) domain, a TGS (ThrRS, GTPase and SpoT) domain and an ACT (aspartokinase, chorismate mutase and TyrA) domain (Fig. S1).

Previously, a high‐throughput screen for genes of Xcc involved in citrus canker symptom development in our laboratory showed that a Tn*5* transposon insertion mutant (*dksA*:Tn*5*) was unable to induce canker symptoms (Yan and Wang, 2012). To explore the effect of stringent response regulators on pathogenicity, three deletion mutants, Δ*dksA*, Δ*relA* and double mutant Δ*spoT*Δ*relA*, were produced by double crossover homologous recombination. We were unable to obtain a *spoT* deletion mutant despite multiple attempts, which is consistent with previous studies in failing to generate the *spoT* mutant in *E. coli*. It was speculated that the hyper‐accumulation of (p)ppGpp due to disruption of *spoT* is lethal to bacteria (Xiao *et al.*, 1991)*.*

**Figure 1:**
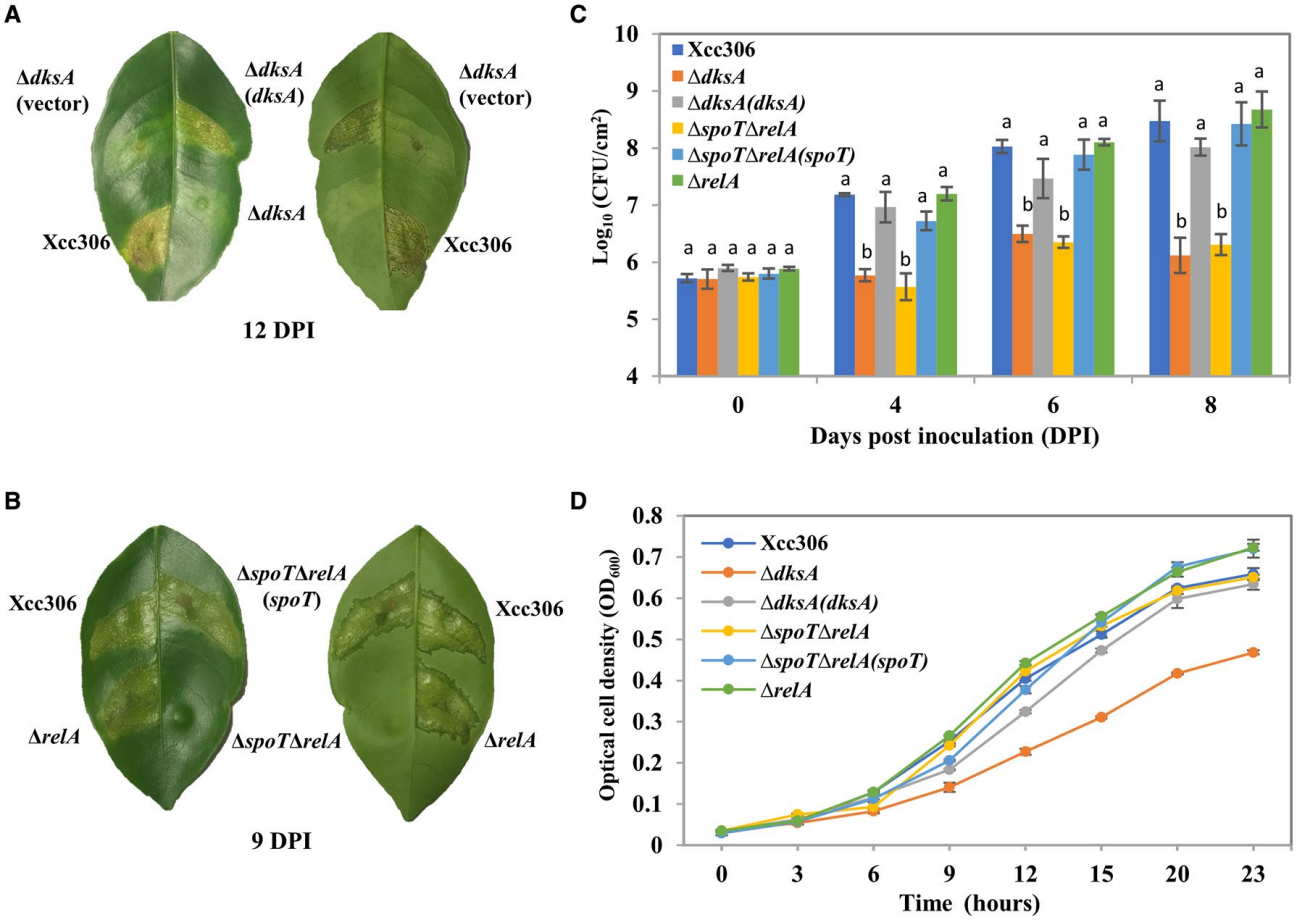
DksA and (p)ppGpp contribute to pathogenicity and bacterial growth *in planta*. (A, B) Bacterial cultures (10^8^ CFU/mL) of the indicated strains were inoculated into sweet orange leaves. Pictures were taken at the indicated time points. (C) Bacterial cultures (10^7^ CFU/mL) of the indicated strains were inoculated into sweet orange leaves and bacterial populations were determined at 0, 4, 6 and 8 days post‐inoculation. (D) Bacterial growth was monitored in the XVM2 medium. (C, D) Values represent means ± SD (*n* = 3). One‐way ANOVA with post hoc Tukey HSD test was applied to compare multiple strains. Statistical significance means *P* < 0.01. All the experiments were repeated three times independently with similar results.

To test the pathogenicity, wild‐type strain Xcc306, Δ*dksA*, Δ*relA*, double mutant Δ*spoT*Δ*relA* and complemented strains carrying recombinant plasmids were inoculated by syringe infiltration onto young Valencia sweet orange (*Citrus sinensis*) leaves. The Δ*dksA* strain with/without empty plasmid did not produce canker symptoms whereas the wild‐type strain and complemented strain caused typical canker symptoms characterized by necrosis with a corky appearance (Fig. 1A). While we did not observe any difference in symptoms between Xcc306 and Δ*relA*, Δ*spoT*Δ*relA* induced much reduced canker symptoms (Fig. 1B). The complemented strain Δ*spoT*Δ*relA* (*spoT*) restored the canker symptoms (Fig. 1B). Consistent with reduced symptom development, bacterial growth of Δ*dksA* and Δ*spoT*Δ*relA* was substantially reduced *in planta* compared to that of the wild‐type Xcc306 and complemented strains (Fig. 1C).

Bacterial growth *in vitro* was also tested in the defined XVM2 medium. The XVM2 medium was reported to mimic the environment of plant intracellular spaces and induce *hrp* gene expression (Astua‐Monge *et al.*, 2005; Wengelnik *et al.*, 1996). Δ*dksA* growth rate was significantly lower than that of the wild‐type strain in the XVM2 medium (Fig. 1D), which might partially explain the reduced canker symptoms of the Δ*dksA* mutant. Complementation of Δ*dksA* through plasmid expression of DksA successfully recovered the growth of Δ*dksA* in the XVM2 medium (Fig. 1D). The Δ*relA* and Δ*spoT*Δ*relA* mutants displayed similar or slightly enhanced growth in the XVM2 medium (Fig. 1D). To sum up, our data show that stringent response regulatory genes *dksA* and *spoT* are required for the pathogenicity of Xcc.

### Transcriptome analysis of Δ*dksA* and Δ*spoT*Δ*relA* mutants compared to the wild‐type Xcc

**Figure 2:**
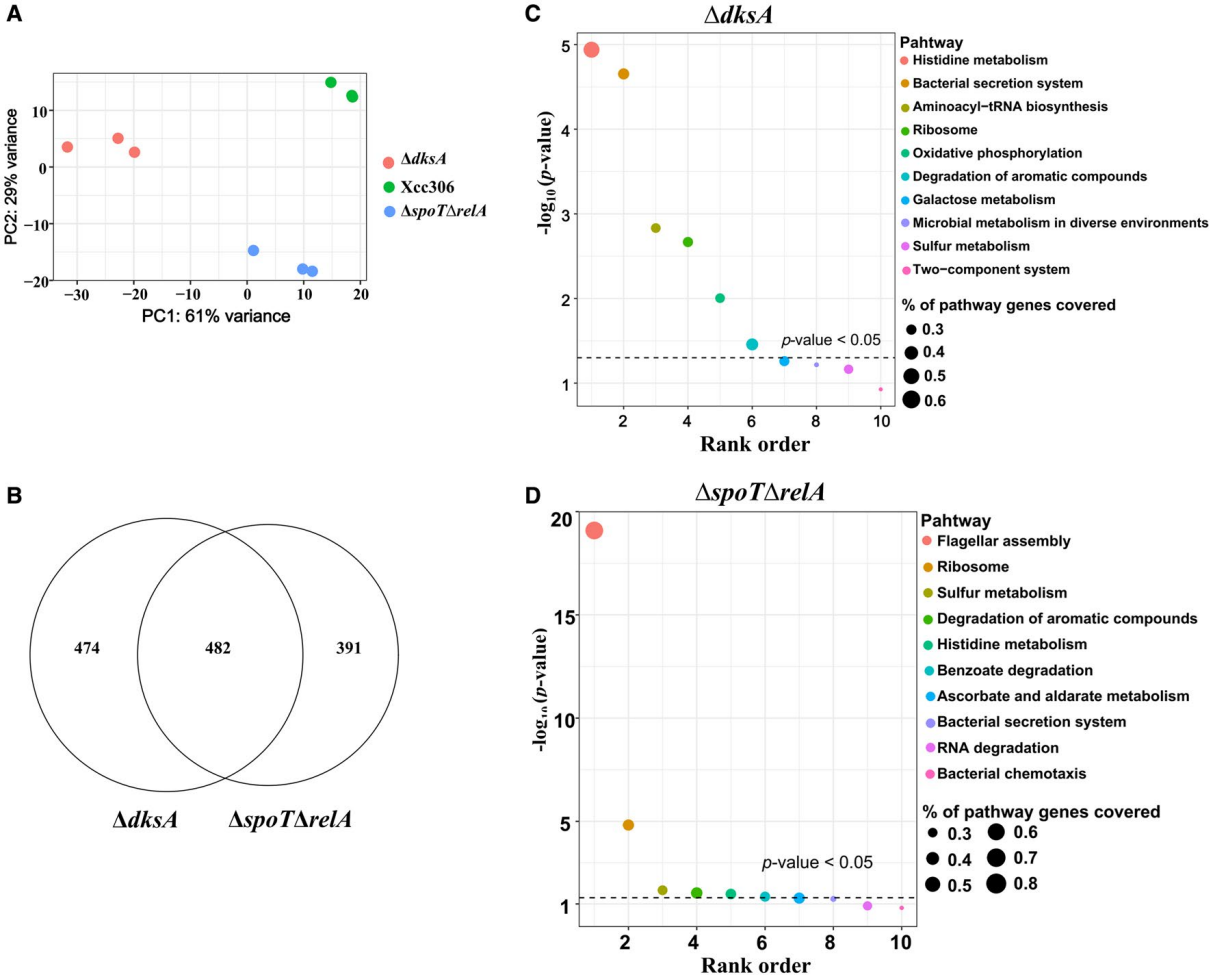
Functional enrichment analysis of differentially expressed genes (DEGs) in the Δ*dksA* and Δ*spoT*Δ*relA* strains compared to wild‐type *Xanthomonas* *citri* subsp. *citri*. (A) Principal component analysis (PCA) of nine bacterial samples. (B) Venn diagram of overlapping DEGs between Δ*dksA* and Δ*spoT*Δ*relA*. (C, D) Pathway enrichment analysis showing the over‐represented pathways for Δ*dksA* (C) and Δ*spoT*Δ*relA* (D). The dashed lines indicate the threshold of statistical significance (*P* = 0.05).

To better understand the function of stringent response regulators in virulence and other cellular processes, RNA‐seq was conducted to determine the transcription profile of wild‐type Xcc306, Δ*dksA* and Δ*spoT*Δ*relA* strains. Bacteria were grown in the XVM2 medium and total RNA was extracted from the mid‐log phase (OD_600_ = 0.35) cultures. In total, nine RNA samples (three biological repeats for each strain) were sent for RNA‐seq (sequencing data information is provided in Table S1). To explore the similarities and dissimilarities between samples, principal component analysis (PCA) was performed. The three biological repeats of each strain clustered together into distinct groups from other strains, indicating that the major variation comes from the difference between each strain (Fig. 2A). Similarly, the heatmap shows that three biological repeats are clustered together (Fig. S2). In addition, RT‐qPCR was conducted to confirm the RNA‐seq data based on selected genes, which shows that log_2_‐transformed values derived from both methods are highly correlated (Fig. S3).

Differentially expressed genes (DEGs) were defined as genes with the absolute value of log_2_ fold change (FC) equal to or more than 2 and adjusted *P*‐value equal to or smaller than 0.01. The The differential gene expression test revealed that in the Δ*spoT*Δ*relA* mutant, 873 genes were identified as DEGs (Fig. 2B) and among them 179 genes were up‐regulated and 694 were down‐regulated (Table S2). In the Δ*dksA* mutant, 956 genes were identified as DEGs (Fig. 2B) and among them 196 genes were up‐regulated and 760 were down‐regulated (Table S3). There are approximately 4500 genes in the Xcc genome, of which 20% were regulated in the Δ*dksA* and Δ*spoT*Δ*relA* mutants. More than 50% of DEGs in both mutants (482 genes) are common, which supports the claim that DksA is the cofactor of (p)ppGpp in many cases (Fig. 2B).

To discover functionality‐related genes regulated by DksA and (p)ppGpp, DEGs were grouped into different functional categories based on the COG database (Fig. S4). The Fisher exact test was conducted to identify the over‐represented functional groups (*P* < 0.05) and showed that both DksA and (p)ppGpp are involved in cell motility, inorganic ion transport and metabolism, and intracellular trafficking and secretion (Table 1). To identify the specific pathways regulated by DksA and (p)ppGpp, pathway enrichment analysis was performed based on the Kyoto Encyclopedia of Genes and Genomes (KEGG) database (Kanehisa and Goto, 2000). The results showed that the over‐represented pathways in both mutant strains included histidine metabolism, bacterial secretion systems, ribosome biosynthesis and degradation of aromatic compounds (*P* value < 0.05) (Fig. 2C,D). Flagellar assembly was the most over‐represented pathway in Δ*spoT*Δ*relA* rather than Δ*dksA*, which indicates the different effect on flagellar assembly between (p)ppGpp and DksA (Fig. 2C,D). To sum up, these results highlight the similarities in the regulons of DksA and (p)ppGpp, and reveal their potential roles.

**Table 1:** Functional enrichment analysis based on COG data

Functional classification (COGs)	Symbol	*P*‐value (Δ*dksA*)	*P*‐value(Δ*spoT*Δ*relA*)
RNA processing and modification	A	NS	NS
Chromatin structure and dynamics	B	NS	NS
Energy production and conversion	C	NS	NS
Cell cycle control, mitosis and meiosis	D	NS	NS
Amino acid transport and metabolism	E	NS	NS
Nucleotide transport and metabolism	F	NS	NS
Carbohydrate transport and metabolism	G	0.0432	NS
Coenzyme transport and metabolism	H	NS	NS
Lipid transport and metabolism	I	NS	NS
Translation, ribosomal structure and biogenesis	J	NS	NS
Transcription	K	NS	NS
Replication, recombination and repair	L	NS	NS
Cell wall/membrane biogenesis	M	NS	NS
Cell motility	N	0.0209	5.5E‐05
Post‐translational modification, protein turnover, chaperones	O	NS	NS
Inorganic ion transport and metabolism	P	0.0004	0.0068
Secondary metabolites biosynthesis, transport and catabolism	Q	NS	NS
General function prediction only	R	NS	NS
Function unknown	S	NS	NS
Signal transduction mechanisms	T	NS	NS
Intracellular trafficking and secretion	U	0.0054	0.0056
Defence mechanisms	V	NS	NS
Extracellular structures	W	NS	NS
Cytoskeleton	Z	NS	NS

NS, no statistical significance.

### DksA and (p)ppGpp repress tRNA and ribosome protein biosynthesis and activate histidine metabolism

**Figure 3:**
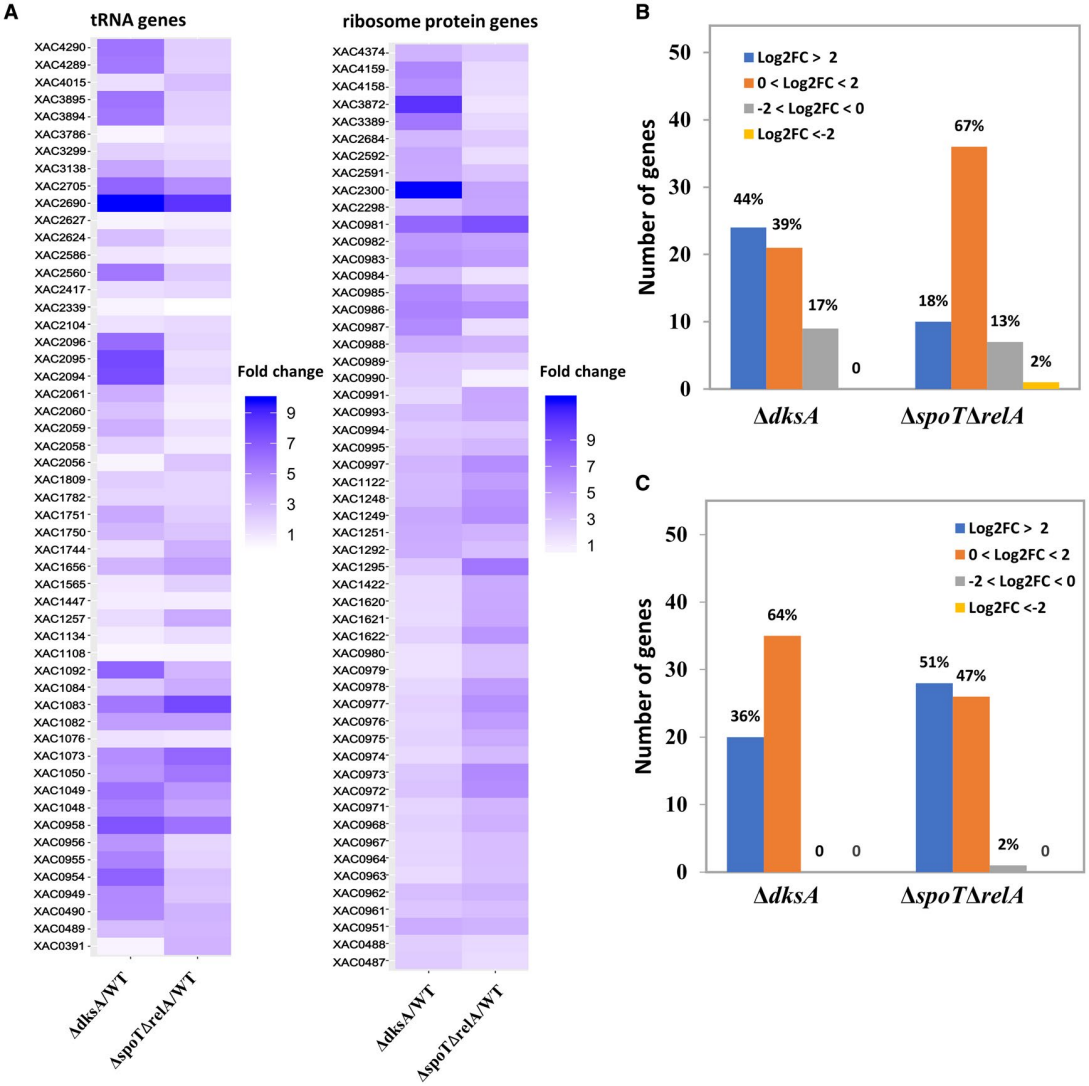
DksA and (p)ppGpp repress gene expression of tRNA and ribosome proteins. (A) Heatmap representing the gene expression profile for tRNA genes and ribosome protein genes. (B, C) Summary of gene expression levels of tRNA genes (B) and ribosome protein genes (C) in the Δ*dksA* and Δ*spoT*Δ*relA* strains. Log2FC refers to log_2_ fold change (Δ/WT; mutant/wild‐type). Data labels indicate the percentage of genes in each category.

A hallmark of the stringent response is the inhibition of stable RNA and ribosome protein biosynthesis (Lemke *et al.*, 2011; Paul *et al.*, 2004). KEGG database‐based enrichment analysis suggested that ribosome biosynthesis was over‐represented in the DksA and (p)ppGpp regulons. We analysed the transcript abundance of genes involved in tRNA and ribosome biosynthesis of Xcc. The relative gene expression levels of 54 tRNA‐encoding genes and 55 ribosome protein‐encoding genes in the Δ*dksA* and Δ*spoT*Δ*relA* mutants compared to the wild‐type Xcc were listed (Tables S4 and S5). Heatmap analysis displayed the relative gene expression profile for both Δ*dksA* and Δ*spoT*Δ*relA* strains compared to the wild‐type Xcc (Fig. 3A). Specifically, 24 of the 54 tRNA genes were up‐regulated DEGs (log_2_FC > 2) in the Δ*dksA* strain (Fig. 3B). Similarly, 10 of the 54 tRNA genes were up‐regulated DEGs (log_2_FC > 2) in the Δ*spoT*Δ*relA* strain (Fig. 3B). For ribosome protein‐encoding genes, 20 and 28 were up‐regulated DEGs (log_2_FC > 2) in Δ*dksA* and Δ*spoT*Δ*relA*, respectively (Fig. 3C)*.*

Studies from *E. coli* have shown that DksA and (p)ppGpp can directly stimulate certain promoters for amino acid biosynthesis and transport (Paul *et al.*, 2005). Based on pathway enrichment analysis, we found that many genes involved in histidine metabolism were down‐regulated in the Δ*dksA* and Δ*spoT*Δ*relA* strains (Fig. S5). Taken together, these results indicate that DksA and (p)ppGpp of Xcc negatively regulate tRNA and ribosome protein biosynthesis and positively regulate histidine metabolism.

### DksA and (p)ppGpp positively regulate T3SS and T2SS

*Xanthomonas* possesses six protein secretion systems (type 1 to type 6). Type 2 secretion system (T2SS) and T3SS have been shown to be important to *Xanthomonas* virulence (Büttner and Bonas, 2010). Functional enrichment analysis showed that bacterial secretion systems are under the control of DksA and (p)ppGpp of Xcc (Table 1). Upon closer inspection, we identified that T3SS and the *xcs* T2SS genes are significantly down‐regulated in the Δ*dksA* and Δ*spoT*Δ*relA* mutants compared to the wild‐type (Table S6). The *hrp*/*hrc* cluster, which encodes the T3SS, is composed of 24 genes distributed in several operons. Twenty‐one (87.5%) and 17 (70.8%) T3SS genes were down‐regulated DEGs in the Δ*dksA* and Δ*spoT*Δ*relA* mutants compared to the wild‐type Xcc, respectively (Table 2). The *xcs* T2SS gene cluster contains 12 genes encoded in a single operon and 11 and 7 T2SS genes are down‐regulated DEGs in the Δ*dksA* and Δ*spoT*Δ*relA* mutants compared to wild‐type Xcc, respectively (Table S6). It should be noted that Xcc harbours a second T2SS (*xps*) that was reported to be more significant to bacterial virulence (Szczesny *et al.*, 2010). No difference was observed in the expression of the *xps* genes in either Δ*dksA* or Δ*spoT*Δ*relA* compared to the wild‐type Xcc.

**Table 2:** Down‐regulated type 3 secretion system (T3SS)‐related genes in mutants compared to wild‐type *Xanthomonas* *citri* subsp. *citri*

Functional group	Genes			
T3SS regulatory gene	*hrpX* ‡			
*hrp*/*hrc* gene cluster	*hpaB*	*hrpD5*	*hpaA*	*hrcS*
	*hrcR*	*hrcQ*	*hpaP*	*hpa2*
	*hrcU*	*hrcN*	*hrcJ*	*hrpB4*
	*hrpB5*	*hpa1*	*hrpB7*	*hrcT*
	*hrcC* †	*hrcV* †	*hrpB1* †	*hrpB2* †
	*hrpF* †	*hrpE* ‡		
T3SS effector genes	*xopI*	*xopK*	*xopN*	*xopZ*
	*xopF*	*xopX* †	*xopM* †	*xopAP* †
	*xopE1* †	*xopAU* †	*xopQ* †	*xopAV* †
	*xopL* †	*xopV* †	*xopP* †	*xopS* ‡
	*avrBs2* ‡			

^†^ Down‐regulated DEGs only in Δ*dksA*.

^‡^ Down‐regulated DEGs only in Δ*spoT*Δ*relA.*

The other genes are down‐regulated differentially expressed genes (DEGs) in both mutant strains.

In addition to the secretion apparatuses, we also observed significant differences in T3SS key regulatory gene *hrpX*, T3SS effector coding genes and T2SS hydrolase genes (Tables 2 and S6). Xcc encodes approximately 30 effector genes as described in the *Xanthomonas* Resource website (http://www.xanthomonas.org/t3e.html). Our data show that 15 (50.0%) and 7 (23.3%) effector genes are down‐regulated DEGs in the Δ*dksA* and Δ*spoT*Δ*relA* mutants compared to the wild‐type Xcc, respectively (Table 2).

**Figure 4:**
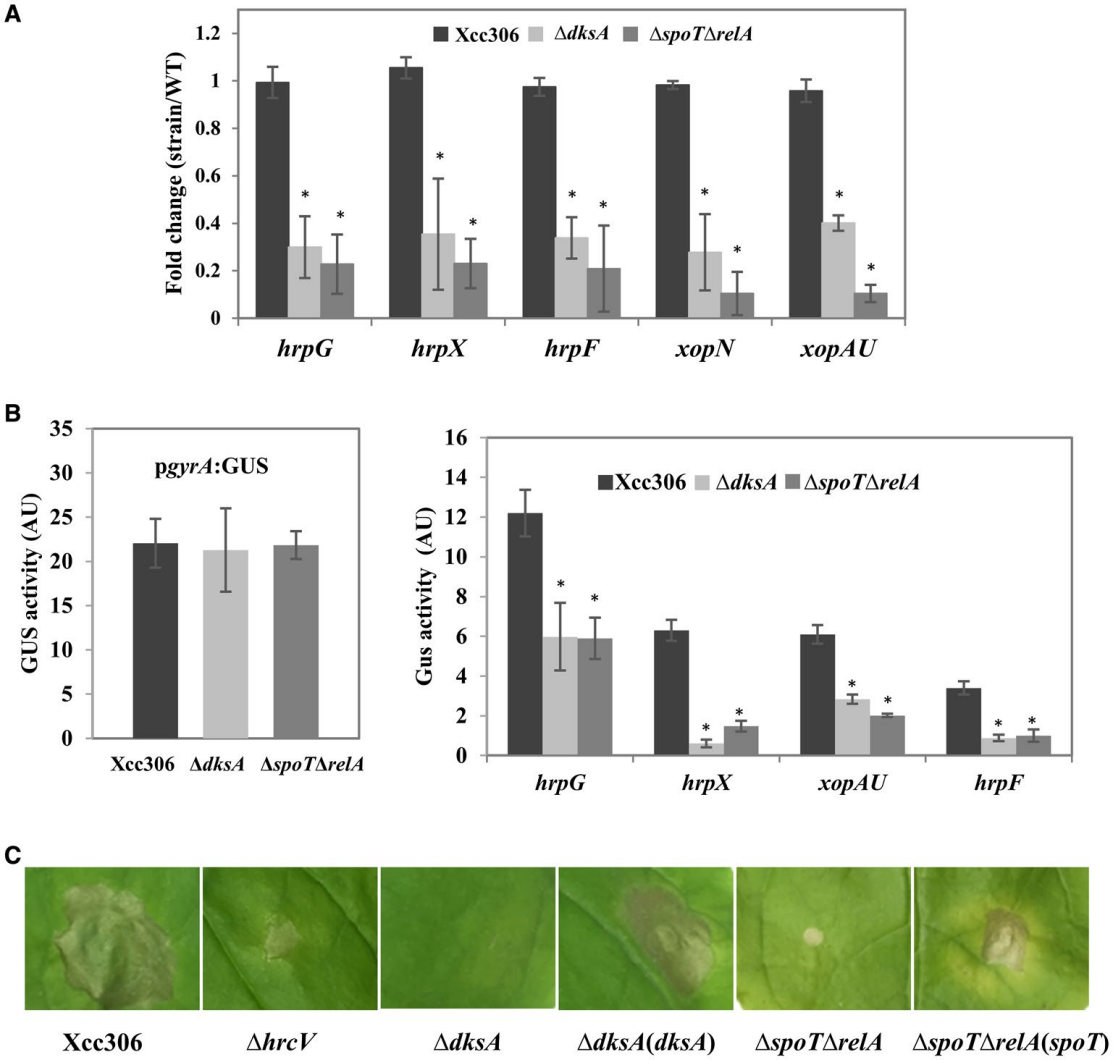
DksA and (p)ppGpp positively regulate expression of the type 3 secretion system (T3SS) genes. (A) mRNA abundance measurement of the selected T3SS genes by RT‐qPCR. For normalization, *gyrA* was used as an endogenous control. (B) Promoter activity of the indicated promoters was measured for Xcc306, Δ*dksA* and Δ*spoT*Δ*relA* strains harbouring translational fusion plasmids. (C) Bacterial cultures (2 × 10^8^ CFU/mL) of the indicated strains were infiltrated into *Nicotiana benthamiana* leaves. Leaves were photographed at 8 days post‐inoculation. (A, B) Values represent means ± SD and asterisks indicate statistical significance using Student’s *t*‐test (*P* < 0.05, *n* = 3). All the experiments were repeated independently three times with similar results.

To confirm the regulatory effect on the T3SS‐related genes, RT‐qPCR was used to measure the mRNA level of selected genes including two regulatory genes (*hrpG, hrpX*), one T3SS component gene (*hrpF*) and two effector genes (*xopN, xopAU*) in the Δ*dksA* and Δ*spoT*Δ*relA* mutants compared to the wild‐type Xcc. The transcript level of selected genes in Δ*dksA* and Δ*spoT*Δ*relA* were two to five times lower than that of wild‐type Xcc306 (Fig. 4A). Consistent with RT‐qPCR results, β‐glucuronidase (GUS) assays using translational fusion plasmids showed that the promoter activity of four selected genes (*hrpG*, *hrpX*, *xopAU*, *hrpF*) in either mutant strain was substantially lower than that of wild‐type Xcc306 (Fig. 4B). T3SS and effectors are known to be responsible for the hypersensitive response (HR) of Xcc on *Nicotiana benthamiana* (Adlung *et al.*, 2016; Sankaranarayanan *et al.*, 2014)*.* To test whether the Δ*dksA* and Δ*spoT*Δ*relA* mutants were affected in the ability to trigger HR, wild‐type strain Xcc306, mutant strains including a T3SS‐disrupted mutant *hrcV*:Tn*5* and complemented strains were inoculated by syringe infiltration on *N. benthamiana*. In contrast to wild‐type Xcc306 and complemented strains, *hrcV*:Tn*5*, Δ*dksA* and Δ*spoT*Δ*relA* were unable to cause HR (Fig. 4C). Taken together, these results indicate that DksA and (p)ppGpp positively regulate the T3SS system to promote virulence.

### Motility‐related structural genes are negatively regulated by DksA and (p)ppGpp

**Figure 5:**
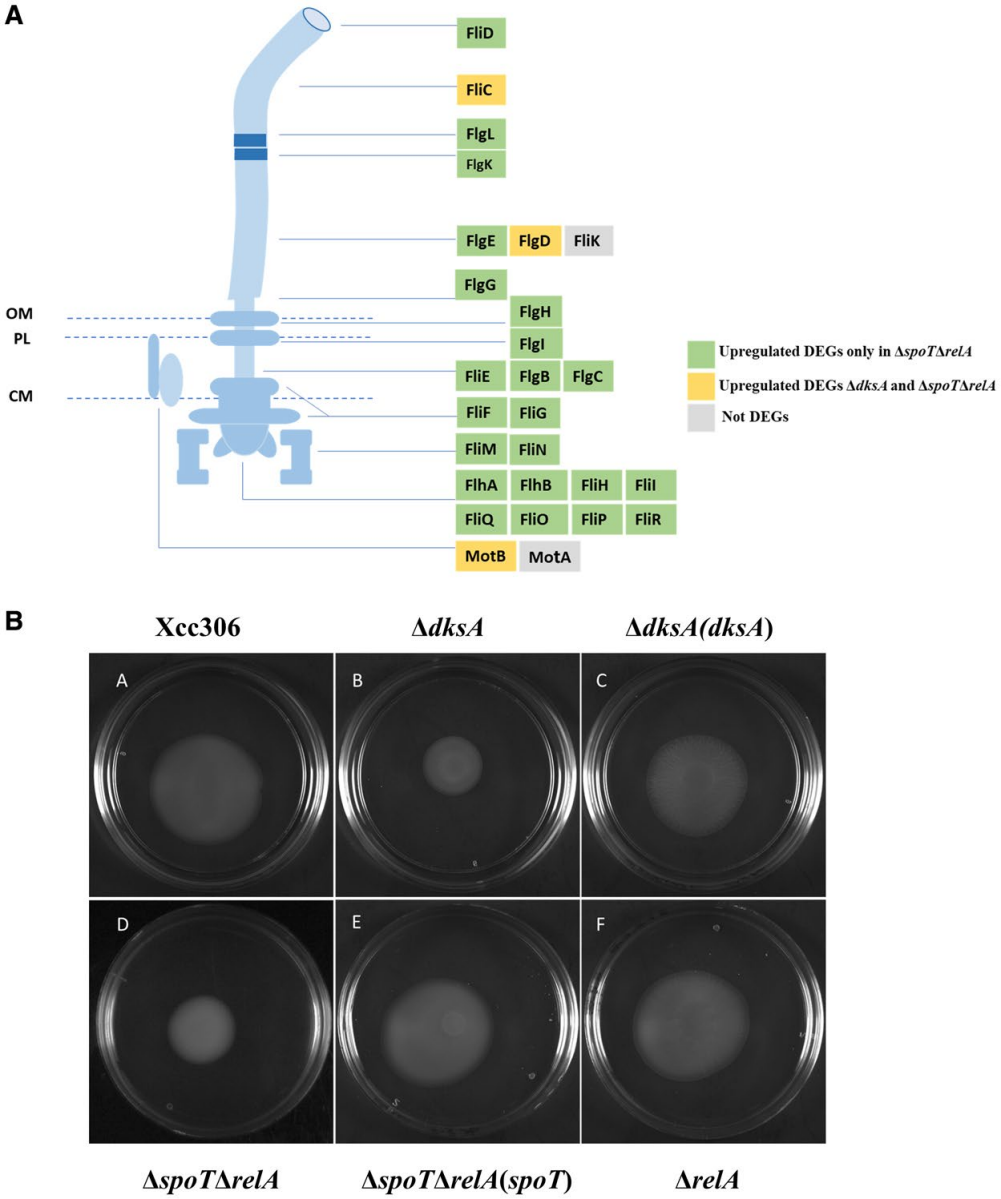
Negative regulation of flagellar assembly genes by (p)ppGpp. (A) Schematic diagram of bacterial flagellum. Gene expression changes in the Δ*dksA* and Δ*spoT*Δ*relA* strains compared to wild‐type *Xanthomonas* *citri* subsp. *citri* (Xcc306) are indicated by different colours. OM, outer membrane; PL, peptidoglycan layer; CM, cytoplasmic membrane. (B) Motility test of bacteria on 0.25% nutrient agar plate. Plates were incubated for 48 h before photographing. The experiment was repeated independently three times with similar results.

Bacterial flagellum biosynthesis consumes considerable metabolic energy and has been considered as a virulence trait (Josenhans and Suerbaum, 2002). Both the flagellum‐dependent and type IV pilus‐dependent motility are present in Xcc and play important roles in adhesion, biofilm formation and virulence (Dunger *et al.*, 2014; Malamud *et al.*, 2011). COG‐based functional and KEGG‐based pathway enrichment analyses showed that motility‐related structural genes were affected in both Δ*dksA* and Δ*spoT*Δ*relA* strains. For flagellar assembly genes, 25 of 27 were up‐regulated DEGs in the Δ*spoT*Δ*relA* strain while only three were up‐regulated DEGs in the Δ*dksA* strain (Fig. 5A). Type IV pilus biogenesis‐related genes displayed different expression patterns: 22 of 28 type IV pilus biogenesis‐related genes, including the regulatory gene *fimX* (XAC2398), were up‐regulated DEGs in Δ*dksA* whereas no up‐regulated DEGs were found in Δ*spoT*Δ*relA* (Table S7)*.*

To test whether the gene expression changes affect flagellar morphology, wild‐type Xcc306, Δ*dksA* and Δ*spoT*Δ*relA* were grown on the XVM2 agar medium for observation under a transmission electron microscope (TEM). No obvious differences in flagellar morphology were observed (Fig. S6). Bacterial motility was tested on 0.25% semi‐solid nutrient agar plates. Unexpectedly, Δ*dksA* and Δ*spoT*Δ*relA* showed reduced motility compared to Xcc306, Δ*relA* and complemented strains (Fig. 5B). To sum up, these results indicate that stringent response regulators DksA and (p)ppGpp inhibit the gene expression involved in flagellar and pili biosynthesis.

### TonB‐dependent transporters genes are positively regulated by DksA and (p)ppGpp

**Figure 6:**
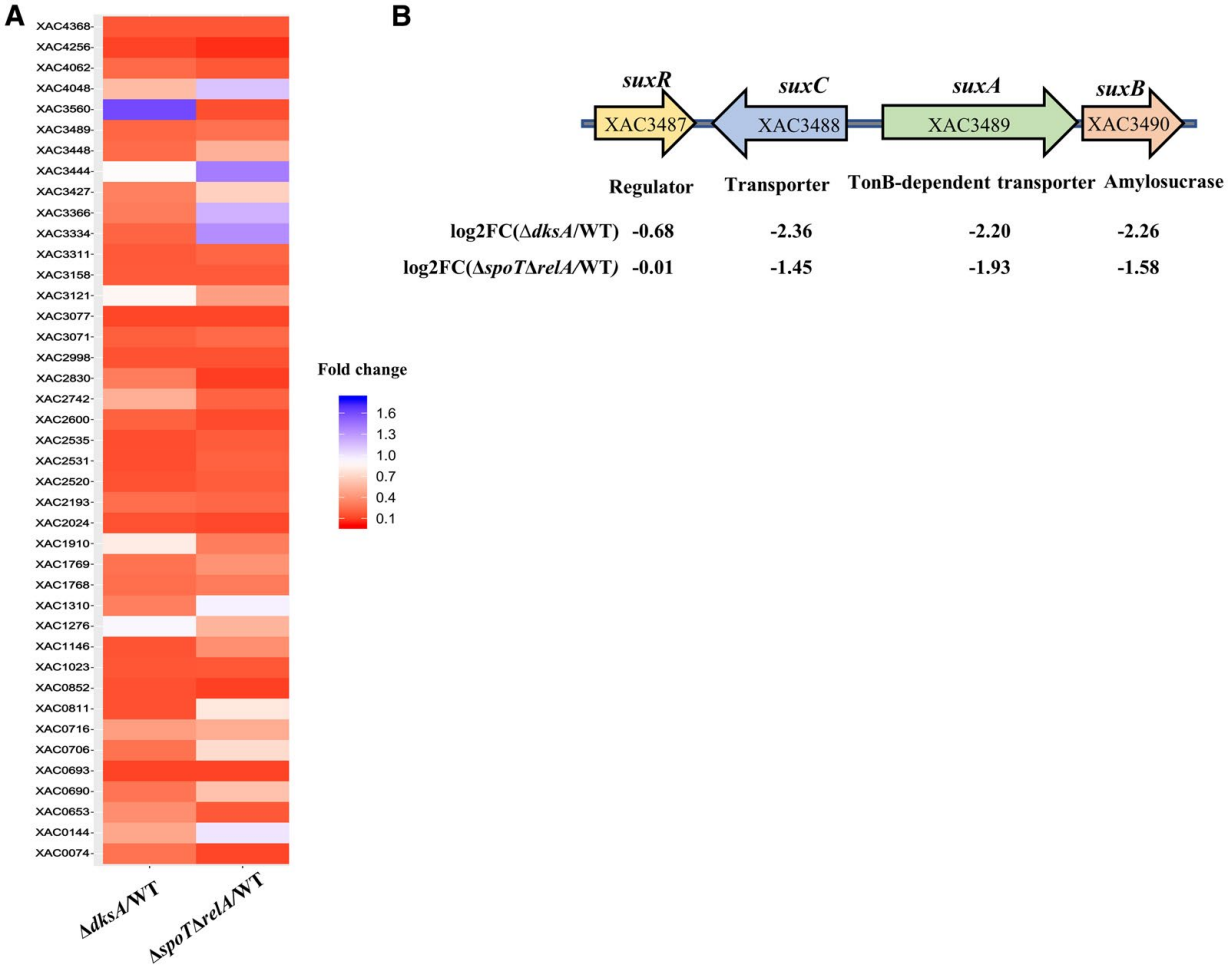
Positive regulation of TonB‐dependent transporter (TBDT) genes by DksA and (p)ppGpp. (A) Heatmap representing the gene expression profile of TBDT genes. (B) The organization of the *sux* locus and the gene expression changes in the Δ*dksA* and Δ*spoT*Δ*relA* strains compared to wild‐type *Xanthomonas citri* subsp. *citri*.

TonB‐dependent transporters (TBDTs) are bacterial outer membrane proteins that are used for active uptake of nutrients such as siderophores, vitamin B12, nickel and carbohydrates in Gram‐negative bacteria (Noinaj *et al.*, 2010; Schauer *et al.*, 2008). Xcc306 encodes 46 TBDTs (da Silva *et al.*, 2002), which are listed in Table S8. Heatmap analysis displayed the gene expression profile of TBDTs in the Δ*dksA* and Δ*spoT*Δ*relA* strains compared to the wild‐type Xcc (Fig. 6A). Among them, 23 (50%) TBDTs were down‐regulated DEGs (log_2_FC > −2) in the Δ*dksA* and Δ*spoT*Δ*relA* mutants compared to the wild‐type Xcc, respectively (Table S8).

A previous study showed that TBDT‐coding genes were over‐represented in *Xanthomonas* spp. and *Xanthomonas campestris* pv. *campestris* employs TBDTs in the sucrose transport and metabolism as well as virulence through a conserved *sux* (sucrose utilization in *Xanthomonas*) locus (Blanvillain *et al*., 2007). The same *sux* locus was also found in Xcc and is composed of four genes encoding a regulatory protein (XAC3487), a sugar inner membrane transporter (XAC3488), a TonB‐dependent transporter (XAC3489) and an amylosucrase (XAC3490) (Fig. 6B). The *suxA*, a TonB‐dependent transporter, *suxB* and *suxC* genes were down‐regulated in both Δ*dksA* and Δ*spoT*Δ*relA* mutants compared to the wild‐type Xcc (Fig. 6B). To sum up, these results indicate that DksA and (p)ppGpp promote the expression of TBDTs, which probably enhance the uptake of nutrients, including sucrose.

### Differential regulation of *xss* gene cluster by DksA and (p)ppGpp

**Figure 7:**
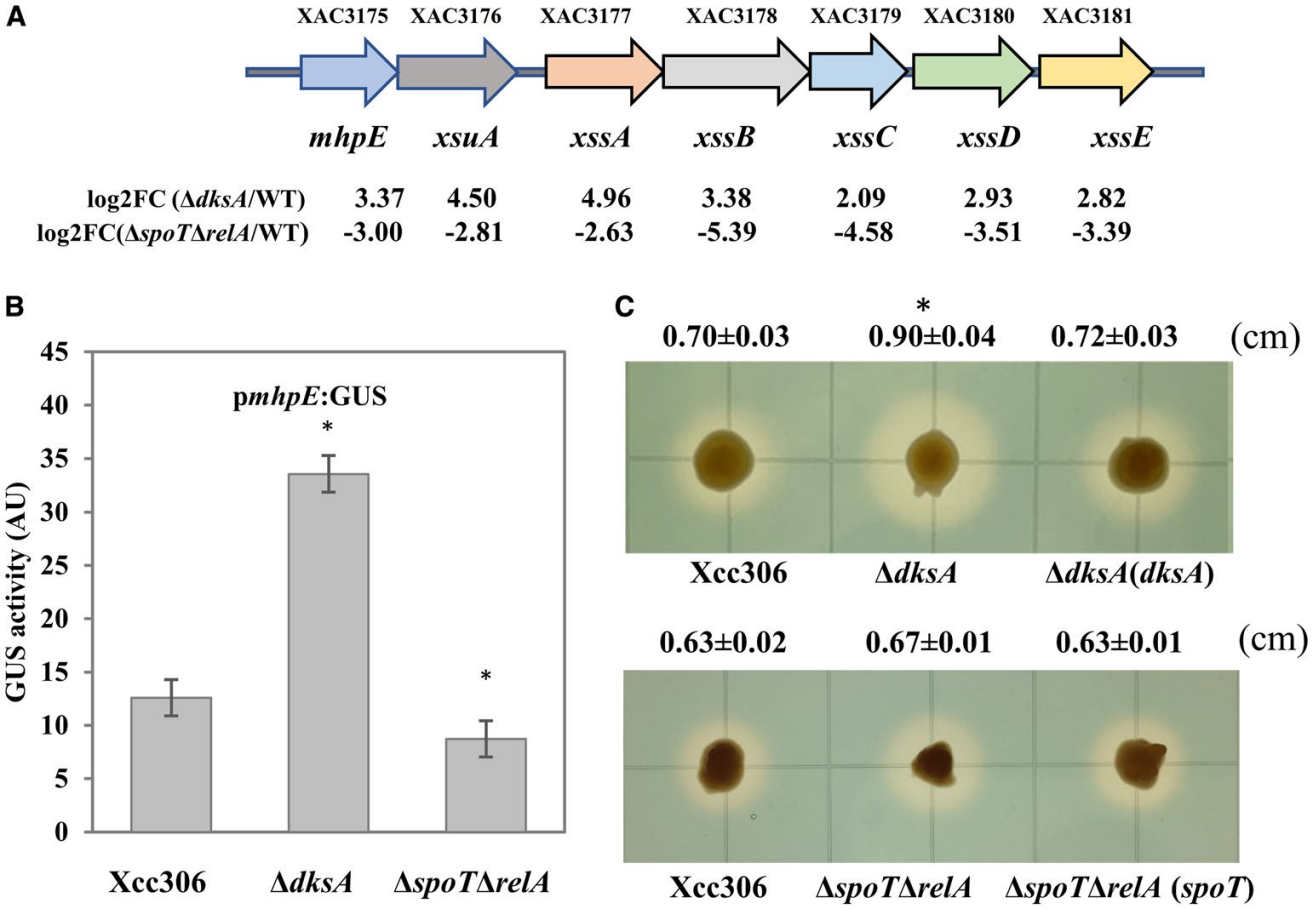
Differential regulation of the *xss* gene cluster by DksA and (p)ppGpp. (A) Organization of siderophore synthesis and utilization gene cluster (*xss)* in *Xanthomonas citri* subsp. *citri* (Xcc306). (B) Promoter activity of *mphE* in Xcc306, Δ*dksA* and Δ*spoT*Δ*relA* strains by β‐glucuronidase (GUS) assay. Values represent means ± SD and asterisks indicate statistical significance using Student’s *t*‐test (*P* < 0.05, *n* = 7). (C) Siderophore production by chrome azurol S (CAS) assay. Fresh bacteria were inoculated on nutrient agar plates supplemented with 200 µM 2,2'‐dipyridyl and photographed after 36 h. Values represent means ± SD of the halo diameter and the asterisk indicates statistical significance using Student’s *t*‐test (*P* < 0.05, *n* = 3). The experiments were repeated independently three times with similar results.

Although DksA mostly acts cumulatively with (p)ppGpp, divergent and even opposite effects on gene expression and specific traits have been reported as well (Lyzen *et al.*, 2009, 2016; Magnusson *et al.*, 2007). We conducted cluster analysis and displayed the gene expression profile of 482 common DEGs in Δ*dksA* and Δ*spoT*Δ*relA* strains (Fig. S7). Among them, 426 genes were down‐regulated and 40 genes were up‐regulated in both mutant strains compared to the wild‐type Xcc. Divergent regulation between Δ*dksA* and Δ*spoT*Δ*relA* was observed for 16 genes (Table S9). Interestingly, we found that the *xss* gene cluster (composed of *mhpE*, *xsuA* and *xssA*‐E), which is involved in biosynthesis, export and utilization of siderophores (Pandey and Sonti, 2010; Pandey *et al.*, 2017), was up‐regulated in Δ*dksA* and down‐regulated in Δ*spoT*Δ*relA* compared to the wild‐type Xcc (Fig. 7A).

Consistent with the RNA‐seq data, the GUS assay showed that the promoter activity of *mphE* is significantly increased in Δ*dksA* and slightly decreased in Δ*spoT*Δ*relA* (Fig. 7B). The ability of these mutant strains to produce siderophores was assessed by a chrome azurol S (CAS) assay. The Δ*dksA* mutant produced a bigger halo around the colonies, indicating more siderophore production (Fig. 7C). However, no obvious difference was observed between wild‐type Xcc306, Δ*spoT*Δ*relA* and Δ*spoT*Δ*relA* (*spoT*) strains (Fig. 7C). Taken together, these results indicate that the *xss* gene cluster is differentially regulated by DksA and (p)ppGpp, and the DksA‐mediated repression of siderophore production might be due to the inhibition of *xss* gene cluster expression.

## Discussion

To adapt to the host environment, plant pathogens need to rely on a sensory system to monitor external signals and subsequently regulate physiological processes and virulence traits. It has long been known that plant pathogens could activate T3SS in contact with host cells (Tang *et al.*, 2006). However, beyond virulence induction, less is known about how plant pathogens prepare for infection regarding physiological changes. Our transcriptome analysis revealed that the stringent response regulator DksA and (p)ppGpp of Xcc have a profound effect on gene expression involved in biosynthesis of stable RNA, ribosome proteins, flagellum and type IV pilus, TBDTs, T2SS and T3SS. Further analysis showed changes in associated traits like pathogenicity, HR induction, motility and siderophore production. Our study further expands the understanding of interplay between the stringent response and virulence.

Deletion of *dksA* or double deletion of s*poT and relA* severely affects bacterial pathogenicity and growth in compatible host plants, indicating that stringent response regulators play an essential role in virulence regulation. Deletion of *relA*, which was reported as the main producer of (p)ppGpp in *E. coli* (Hauryliuk *et al.*, 2015), had little to no effect on the virulence of Xcc. We speculate that since SpoT has weak (p)ppGpp synthetase activity, a certain amount of (p)ppGpp might be present in the Δ*relA* strain, thus masking the effect of *relA* mutation.

The contribution of DksA and/or (p)ppGpp to virulence was reported in multiple animal and plant pathogenic bacteria such as *Pseudomonas aeruginosa*, *Vibrio cholera*, *Streptococcus pneumonia*, *Salmonella enterica*, enterohaemorrhagic *E. coli* (EHEC), *E. amylovora* and *P. syringae* (Ancona *et al.*, 2015; Azriel *et al.*, 2015; Bhadra *et al.*, 2012; Chatnaparat *et al.*, 2015a,b; Kazmierczak *et al.*, 2009; Nakanishi *et al.*, 2006; Xu *et al.*, 2016). In EHEC, (p)ppGpp and DksA could directly activate transcription of two transcriptional regulators, Pch and Ler, which control the gene expression of T3SS and secreted proteins (Nakanishi *et al.*, 2006). Consistent with previous study in other microorganisms, DksA and (p)ppGpp also regulate T3SS genes in Xcc, even though the detailed mechanism remains to be determined. Besides T3SS, our study also provides evidence indicating that T2SS is also under the control of DksA and (p)ppGpp, which further explains their contribution to Xcc virulence.

Under nutrient limitation, (p)ppGpp and DksA inhibit the biosynthesis of ribosome and cell surface organelles (flagella and pili), which is consistent with the high amount of energy needed in synthesizing those macromolecular complexes. For example, about 2% of biosynthetic energy was required for *E. coli* flagellar synthesis (Soutourina and Bertin, 2003). The negative regulation of ribosome and/or flagella biosynthesis was also observed in *E. coli* by DNA microarray (Durfee *et al.*, 2008; Traxler *et al.*, 2008). Another *in vitro* study showed that DksA and (p)ppGpp directly regulate the flagellar cascade of *E. coli* by inhibiting the transcription of two regulatory genes *flhDC* and *fliA* (Lemke *et al.*, 2009). It is possible that Xcc may adopt a similar mechanism to regulate the flagellar transcriptional cascade. Interestingly, our RNA‐seq data indicate that in Xcc, (p)ppGpp has a strong negative effect on flagellar assembly whereas DksA mainly controls gene expression of type IV pili. Despite the higher expression of flagellar genes in the Δ*dksA* and Δ*spoT*Δ*relA* mutants compared to the wild‐type Xcc, reduced motility was observed for both mutants, which requires further investigation.

One striking feature identified in our analysis is the significant down‐regulation of TBDTs in the Δ*dksA* and Δ*spoT*Δ*relA* strains compared to the wild‐type Xcc. One community proteogenomics study revealed that various TBDTs were highly expressed in phyllosphere bacteria from soybean, clover and *Arabidopsis thaliana* plants, indicating the importance of TBDTs in microbial adaptation to the phyllosphere (Delmotte *et al.*, 2009). Interestingly, TBDTs are significantly over‐represented in *Xanthomonas* genomes compared to other bacteria (Schauer *et al.*, 2008). Although it is unclear whether over‐represented TBDTs are required for epiphytic fitness of *Xanthomonas*, the involvement of TBDTs in carbohydrate uptake and virulence was proposed (Blanvillain *et al*., 2007; Boulanger *et al.*, 2010). Given that some effectors target plant *SWEET* genes to cause nutrient efflux for the benefit of some bacterial pathogens, one interesting question remaining to be addressed is whether effector‐mediated sucrose efflux could be acquired by bacterial TBDTs (Jacques *et al.*, 2016).

Due to the low solubility and potential toxicity of iron, bacteria have evolved several iron acquisition mechanisms, including a siderophore‐based iron transport system, and iron homeostasis is tightly regulated in the cell (Andrews *et al.*, 2003). The comparison of the DksA and (p)ppGpp regulons indicated that the *xss* gene cluster involved in siderophore production, transport and metabolism is differentially regulated. The GUS assay showed that the promoter activity of *mphE* was increased significantly in Δ*dksA* and slightly decreased in Δ*spoT*Δ*relA* compared to the wild‐type Xcc. Consistent with this result, more siderophore production was observed in Δ*dksA*. The relationship between iron uptake and stringent response regulators is not fully understood. It was reported that iron deprivation induces SpoT‐dependent accumulation of (p)ppGpp, which increases the expression of iron uptake genes (Vinella *et al.*, 2005). Another study from *S. enterica* suggested that DksA represses the expression of some iron homeostasis‐related genes in response to nitrosative stress (Crawford *et al.*, 2016). Our data show that DksA suppresses the expression of the iron‐uptake related genes such as the *xss* cluster under nutrient‐rich conditions whereas (p)ppGpp has an opposite regulation effect on those genes. It is possible that the interplay between these two factors enables bacteria to maintain iron homeostasis.

**Figure 8:**
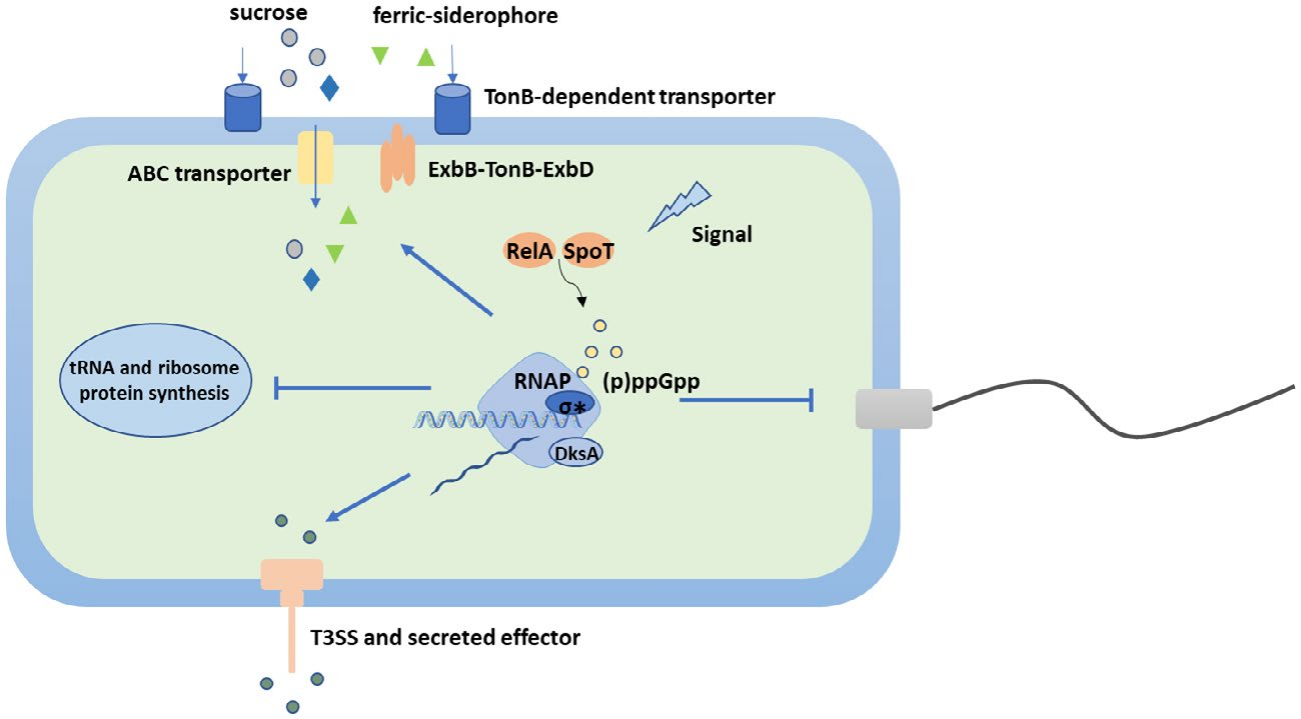
A working model of stringent response regulators of *Xanthomonas citri* subsp. *citri* during host colonization. ↓, positive regulation; ⊥, negative regulation. Regulatory steps in the model are mainly at the transcriptional level.

Based on our findings, a working model was proposed to demonstrate how DksA and (p)ppGpp contribute to Xcc regulation of different traits during colonization (Fig. 8). For survival and multiplication in the host apoplastic space, Xcc needs to conquer many barriers, including low nutrients, iron stress and plant immunity defence. These unfavourable stress conditions potentially activate a stringent response in the bacteria. In this case, accumulated (p)ppGpp together with DksA will relocate cellular resources and save biosynthetic energy by repressing the biosynthesis of stable RNA, ribosome proteins, flagella and type IV pili. Meanwhile, expression of genes for T3SS and TBDTs are enhanced to promote the bacterial virulence and nutrient uptake. The sophisticated control of different traits involving DksA and (p)ppGpp helps the pathogens achieve a balance between fitness and virulence, and contribute to host adaptation and colonization.

## Experimental Procedures

### Bacterial strains, growth conditions and plasmids

The strains, plasmids and primers used in this study are listed in Tables S10 and S11. *Escherichia coli* cells were cultured in lysogeny broth (LB) medium at 37 °C. *Xanthomonas* strains were cultured in nutrient broth (NB) medium and on nutrient agar (NA) plates at 28 °C. For induction of *hrp* genes, Xcc strains were grown in XVM2 medium (Wengelnik and Bonas, 1996). Concentrations of antibiotics were as follows: ampicillin (100 µg/mL), kanamycin (50 µg/mL), gentamycin (10 µg/mL) and spectinomycin (100 µg/mL).

### Generation of mutant strains and complemented strains

The procedures for generating deletion mutants are described elsewhere and were used with slight modification (Murphy *et al.*, 2000). Briefly, the genomic DNA template was extracted using a Genomic DNA Purification Kit (Promega, Madison, WI, USA). For each target gene (*dksA*, *relA* and *spoT*), the upstream and downstream flanking regions were amplified. A second overlap PCR was performed to connect two fragments using the forward primer of the upstream region and the reverse primer of the downstream region (Table S11). The whole fragment was sequenced and subsequently inserted into the multiple cloning sites of the pOK1 suicide vector (Huguet *et al*., 1998). The vector was transformed into Xcc by electroporation and markerless deletion mutants were produced using a two‐step sucrose counterselection procedure (Zhou *et al.*, 2015). Note that the double mutant Δ*spoT*Δ*relA* was generated in the background of Δ*relA*.

To construct the complemented strains, DNA fragments covering the entire coding region and the promoter region of target genes were amplified and inserted into the multiple cloning site of the plasmid pBBR1MCS‐2 (Kovach *et al.*, 1995). Plasmids were introduced into corresponding mutant strains by electroporation.

### RNA extraction, sequencing and data analysis

Fresh colonies of Xcc306, Δ*dksA* and Δ*spoT*Δ*relA* strains were picked up from NA plates and grown overnight in NB medium at 28 °C. Three biological repeats were used for each strain. Bacterial cells were harvested and washed before inoculation into the XVM2 medium. On reaching the exponential stage (OD_600_ = 0.35), bacterial cells were mixed with two volumes of RNA protect bacterial reagent (Qiagen, Valencia, CA, USA) and RNA was extracted following the instructions of the RNeasy Mini Kit (Qiagen). Residual genomic DNA was removed by a TURBO DNA‐free kit (Ambion, Austin, TX, USA). The mRNA enrichment and library construction were described elsewhere (Jalan *et al.*, 2013). Briefly, the ribosomal RNA was removed using the Ribo‐Zero™rRNA Removal Kit for bacteria (Illumina, Madison, USA) according to the manufacturer’s instructions. The remaining transcripts were fragmented and cDNA was synthesized using mRNA templates via reverse transcription. cDNA libraries were constructed using the TrueSeq Stranded mRNA Sample Prep kit (Illumina). Paired end reads (150 bp) were generated for all the RNA samples using a Hiseq 3000 sequencer platform (Novo Gene, Beijing China). The RNA raw reads were deposited at the NCBI SRA database under the bio‐project accession no. PRJNA513356.

To determine the DEGs, mapping reads to the reference genome, transcript abundance quantification and differential expression analysis were performed using Rockhopper (McClure *et al.*, 2013). The DEGs were selected based on an absolute value of log_2_ fold change (mutant strain/wild‐type strain) greater than or equal to 2 and an adjusted *P*‐value less than or equal to 0.01. The R package DESeq2 (v. 1.20.0) (Love *et al.*, 2014) and Cluster 3.0 (de Hoon *et al.*, 2004) were used for PCA analysis and cluster analysis. Based on the KEGG pathway database, clusterProfiler (v. 2.8.1) was used for pathway enrichment analysis (Yu *et al*., 2012). Enrichment analysis of all DEGs based on the Clusters of Orthologous Groups (COGs) of proteins database was performed using Fisher’s exact test (Abatangelo *et al.*, 2009).

### Pathogenicity tests, *in planta* bacterial growth and HR tests

Pathogenicity assays were performed in a quarantine greenhouse located at the Citrus Research and Education Center, Lake Alfred, FL, USA. The wild‐type strain Xcc306, Δ*dksA*, Δ*relA*, Δ*spoT*Δ*relA* and complemented strains were cultured overnight in NB medium at 28 °C. After centrifugation, bacterial pellets were washed and resuspended in sterile water. The concentration of bacterial solution was adjusted to 10^8^ (for monitoring symptoms in sweet orange), 2 × 10^8^ (for monitoring HR) or 10^7^ (for monitoring bacterial growth) CFU/mL (Andrade *et al.*, 2014; Teper *et al.*, 2019; Yan and Wang, 2012; Zhou *et al.*, 2018). Bacteria were infiltrated into immature leaves of sweet orange (*C. sinensis*) and *N. benthamiana.*

To measure the bacterial population, three leaf disks were taken out from inoculation sites per strain per time point. Leaf disks with a diameter of 0.6 cm were put into a 1.5 mL Eppendorf tube containing sterile water and ground by a drill. The bacterial solution was serially diluted and spotted on NA plates for incubation at 28 °C. The bacterial population given as CFU/mL was calculated after 48 h.

### *In vitro* bacterial growth

Fresh overnight bacterial culture was inoculated into a 50 mL centrifuge tube containing 10 mL NB or XVM2 media at an initial optical density of OD_600_ = 0.05. At each time point, 200 µL bacterial culture of each strain was taken out and measured using microplate spectrophotometer (Bio‐Rad Laboratories, Hercules, CA, USA). The experiments were repeated at least twice in triplicate with similar results.

### RT‐qPCR

For two‐step quantitative reverse transcription PCR (RT‐qPCR), 1 µg RNA was used for cDNA synthesis using a qScript cDNA Synthesis Kit (Quantabio, Beverly, MA, USA). Primers were designed by online software Primer 3 (http://bioinfo.ut.ee/primer3-0.4.0/). All primer sequences are listed and classified in Table S11. The reaction mixture was prepared following the instructions for the SYBR Green Master Mix kit (Clontech Laboratories, Mountain View, CA, USA) on a QuantStudio 3 Real‐Time PCR System (Thermo Fisher Scientific, Waltham, MA, USA). The expression level of *gyrA* was used as an endogenous control. The fold change of target genes expression was calculated using the formula 2^–ΔΔCt^ (Livak and Schmittgen, 2001). This experiment was repeated three times in triplicate with similar results.

### Motility assay

Bacterial motility was tested using a semisolid NA plate containing 0.3% agar. Bacteria were grown in NB overnight with shaking at 200 rpm, and then centrifuged down, washed and diluted to OD_600_ = 0.5 in sterile water. A 5 µL suspension of each strain was spotted on the centre of the plates and incubated at 28 °C. Plates were photographed after 48 h. The assay was repeated three times independently in triplicate with similar results.

### GUS activity assay

To generate GUS reporter plasmids the putative promoter region of the tested genes was amplified and cloned into pGUS vector (Teper *et al.*, 2019) and transferred into wild‐type Xcc306 and mutant strains by electroporation.

GUS activity was quantified as described previously (Zhou *et al.*, 2017) with slight modifications. Briefly, bacterial cells grown in the XVM2 medium were harvested and resuspended in phosphate‐buffered saline (PBS) and followed by sonication. A volume of 20 µL clear supernatant was added to 80 µL PBS buffer containing 1.25 µM *p*‐nitrophenyl β‐d‐glucopyranoside (PNPG) in a 96‐well plate. When a yellow colour developed during incubation at 37 °C, 100 µL stop buffer (0.4 M Na_2_CO_3_) was added to stop the reaction immediately. Meanwhile, the reaction time was recorded. The absorbance of the reaction solution was measured at 405 nm and normalized by a protein amount that was measured by the Bradford method at 595 nm using a Bio‐Rad Protein Assay Kit (Bio‐Rad). GUS activity was quantified by arbitrary units (AU) and determined as A_405_/(time in min × A_595_ × 0.02). The experiment was repeated at least twice in triplicate with similar results.

### Siderophore production assay

The CAS assay was conducted as described elsewhere (Cordero *et al.*, 2012). Briefly, 1 L of CAS‐Fe‐hexadecyl‐trimethyl‐ammonium bromide (HDTMA) dye was prepared as follows: 10 mL of a 10 mM ferric chloride (FeCl_3_) in 100 mM HCl solution was mixed with 590 mL of a 1 mM aqueous solution of CAS. The Fe‐CAS solution was then added to 400 mL of 2 mM aqueous solution of HDTMA. The resulting CAS‐Fe‐HDTMA solution was autoclaved for 20 min. The solution was stored at room temperature and covered with aluminium foil. For 100 mL of CAS‐agar, 10 mL of CAS‐Fe‐HDTMA dye was mixed with 90 mL of NB‐based media supplemented with 2,2ʹ‐dipyridyl (DP). To prepare samples, bacterial strains grown on NA plates were spotted onto the CAS agar plate and incubated at 28 °C for 36 h. Halo phenotype around colonies is indicative of siderophore production. The assay was repeated three times independently with similar results.

## Supporting information


**Fig. S1** Protein domain analyses of DksA, RelA and SpoT in Xcc. The domains of the DksA, SpoT and RelA are shown. Protein sequence analysis was performed using online software InterPro (https://www.ebi.ac.uk/interpro/).

**Fig. S2** Heatmap analysis of the sample‐to‐sample distances. An overview over similarities and dissimilarities between samples. Sample‐to‐sample distances were calculated from a count matrix based on all chromosome genes. The heatmap was derived from the distance matrix using R package DESeq2 (v. 1.20.0).

**Fig. S3** Comparison of gene expression by RT‐qPCR and RNA‐seq. At least 16 genes were randomly selected for Δ*dksA *and Δ*spoT*Δ*relA *strains. The mRNA abundance of these genes was tested by RT‐qPCR using *gyrA *as an endogenous control. The R‐squared value and the *P*‐value were calculated by R software (v. 3.5.1).

**Fig. S4** Distribution of DEGs of Δ*dksA* and Δ*spoT*Δ*relA* compared to wild‐type Xcc according to COG categories. For Xcc306, the numbers mean the total genes in each category according to the Xanthomonas Genome Browser (http://xgb.leibniz‐fli.de/cgi/cog.pl?ssi=free).

**Fig. S5** Down‐regulated DEGs involved in the histidine metabolism pathway. Genes in red represent down‐regulated DEGs in the Δ*dksA* and Δ*spoT*Δ*relA *mutants compared to wild‐type Xcc, genes in orange represent down‐regulated DEGs only in Δ*dksA*, genes in green represent down‐regulated DEGs only in Δ*spoT*Δ*relA*. This pathway is based on the KEGG pathway database (https://www.genome.jp/kegg‐bin/show_pathway?xac00340).

**Fig. S6** Transmission electron microscopic observation of Xcc306, Δ*dksA* and Δ*spoT*Δ*relA* strains. Fresh bacteria of Xcc306, Δ*dksA* and Δ*spoT*Δ*relA *from XVM2 solid agar medium were transferred to formvar/carbon‐coated 400‐mesh copper grids and stained with 0.25% aqueous ammonium molybdate. The grids were allowed to dry for 1 h before observation with an FEI Morgagni 268 transmission electron microscope (FEI, OR, USA). The black arrows indicate bacterial polar flagella.

**Fig. S7** Gene expression profile of common DEGs for Δ*dksA* and Δ*spoT*Δ*relA*. Hierarchical cluster analysis shows that all common DEGs were grouped into four clusters: I, II, III and IV. Forty genes (IV) were up‐regulated, and 426 genes (II) were down‐regulated in both mutants. Twelve genes (I) were up‐regulated in Δ*dksA* but down‐regulated in Δ*spoT*Δ*relA*. Four genes (III) were down‐regulated in Δ*dksA* but up‐regulated in Δ*spoT*Δ*relA*.

**Table S1** Data quality summary.

**Table S2** Differentially regulated genes in the ΔspoTΔrelA mutant compared to the wild‐type Xcc.

**Table S3** Differentially regulated genes in the ΔdksA mutant compared to wild‐type (WT) Xcc.

**Table S4** Gene expression of tRNA‐coding genes.

**Table S5** Gene expression of ribosome protein genes.

**Table S6** Gene expression profile of T3SS‐ and T2SS‐related genes in Xcc.

**Table S7** Gene expression profile for putative type 4 pilus biogenesis and regulation genes of Xcc.

**Table S8** Gene expression level of TBDT genes of Xcc.

**Table S9** Differentially regulated genes by DksA and ppGpp.

**Table S10** Strains and plasmids used in this study.

**Table S11** Primer sequence used in this study.

## Data Availability

The data that support the findings of this study are available from the corresponding author on reasonable request.
