# Supplementary figures and images for: Stringent response regulators (p)ppGpp and DksA positively regulate virulence and host adaptation of Xanthomonas citri
Source: Mol Plant Pathol. 2019 Oct 17;20(11):1550–65. doi: 10.1111/mpp.12865 (PMC6804348; doi:10.1111/mpp.12865)

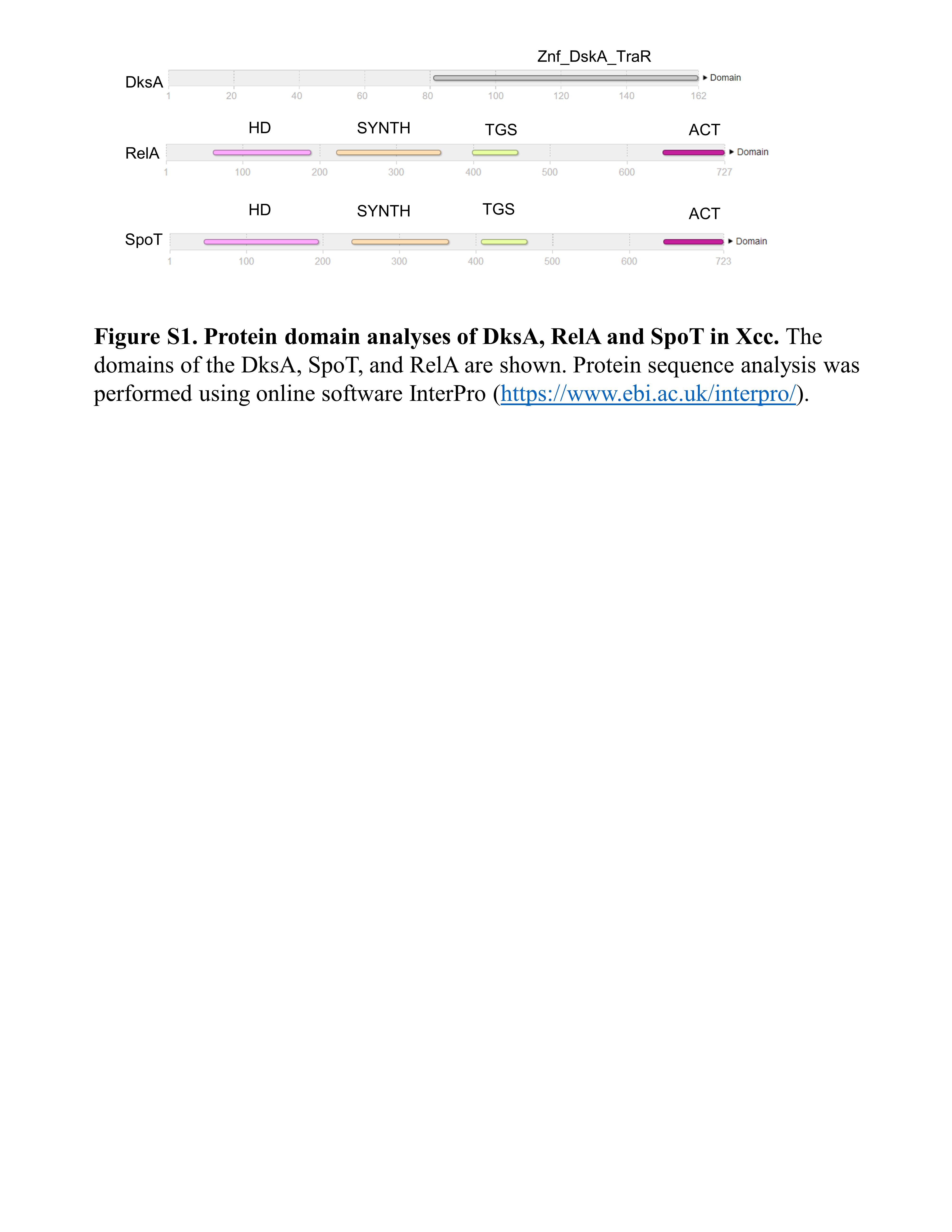

Supplement: Supplementary file 1 — Fig. S1 Protein domain analyses of DksA, RelA and SpoT in Xcc. The domains of the DksA, SpoT and RelA are shown. Protein sequence analysis was performed using online software InterPro (https://www.ebi.ac.uk/interpro/). [file MPP-20-1550-s001.TIF]

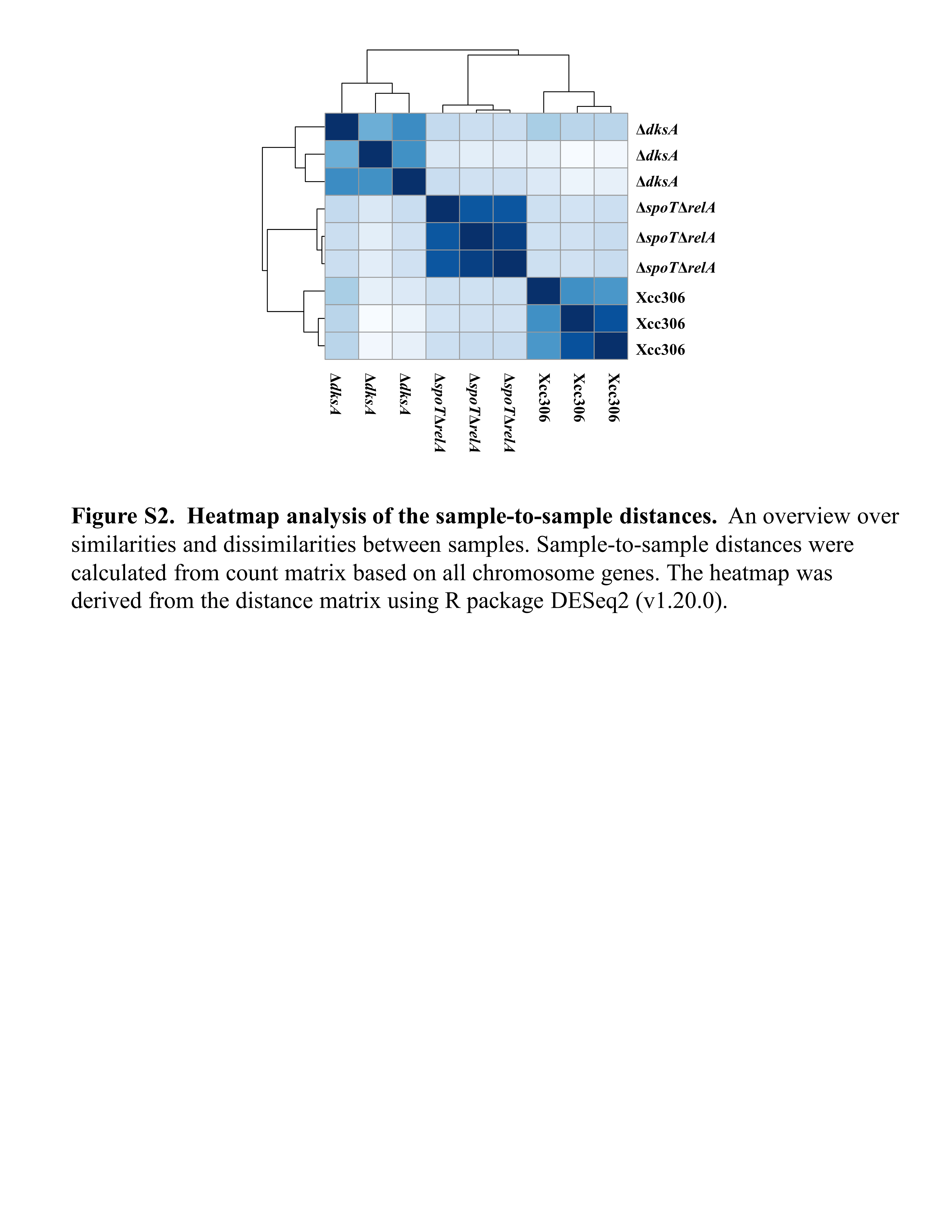

Supplement: Supplementary file 2 — Fig. S2 Heatmap analysis of the sample‐to‐sample distances. An overview over similarities and dissimilarities between samples. Sample‐to‐sample distances were calculated from a count matrix based on all chromosome genes. The heatmap was derived from the distance matrix using R package DESeq2 (v. 1.20.0). [file MPP-20-1550-s002.TIF]

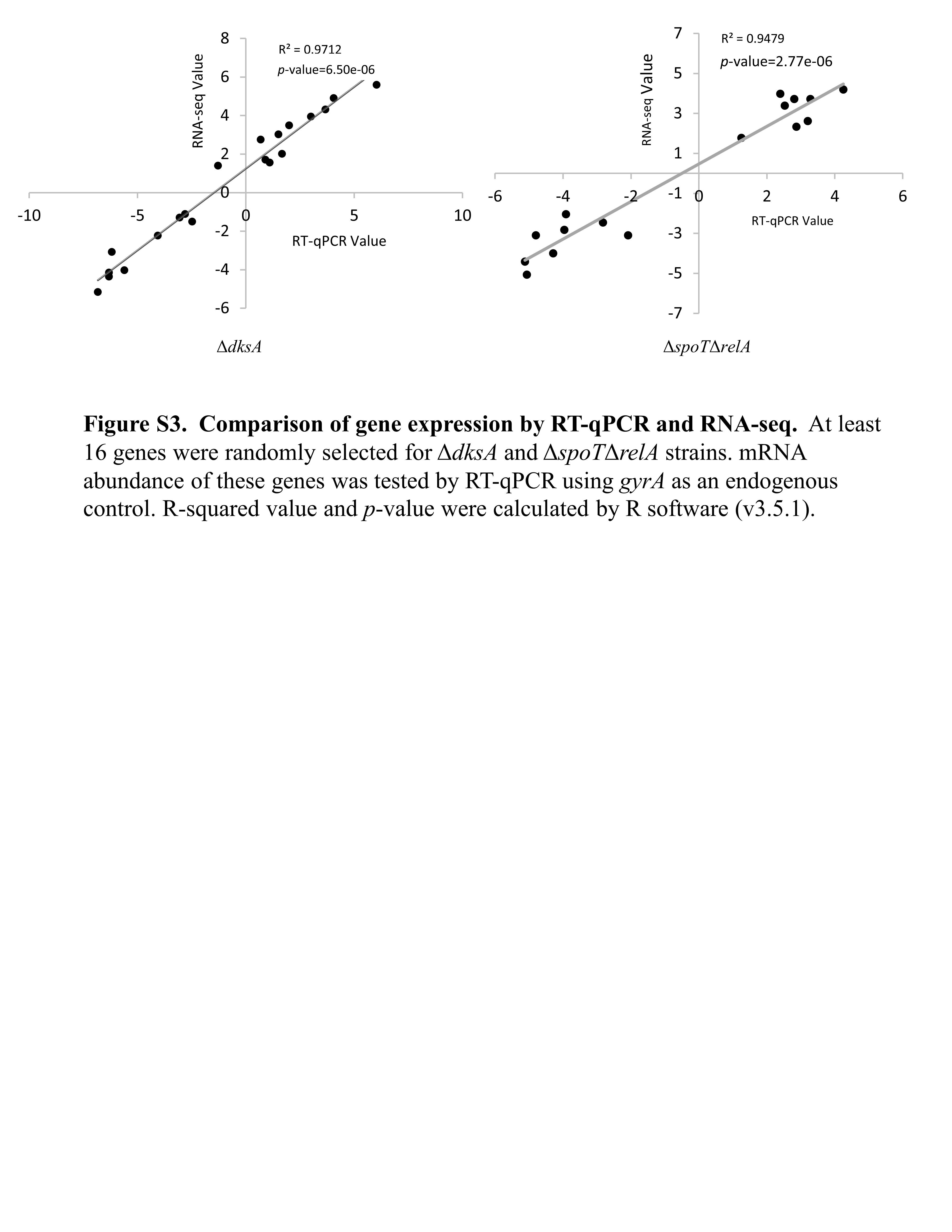

Supplement: Supplementary file 3 — Fig. S3 Comparison of gene expression by RT‐qPCR and RNA‐seq. At least 16 genes were randomly selected for ΔdksA and ΔspoTΔrelA strains. The mRNA abundance of these genes was tested by RT‐qPCR using gyrA as an endogenous control. The R‐squared value and the P‐value were calculated by R software (v. 3.5.1). [file MPP-20-1550-s003.TIF]

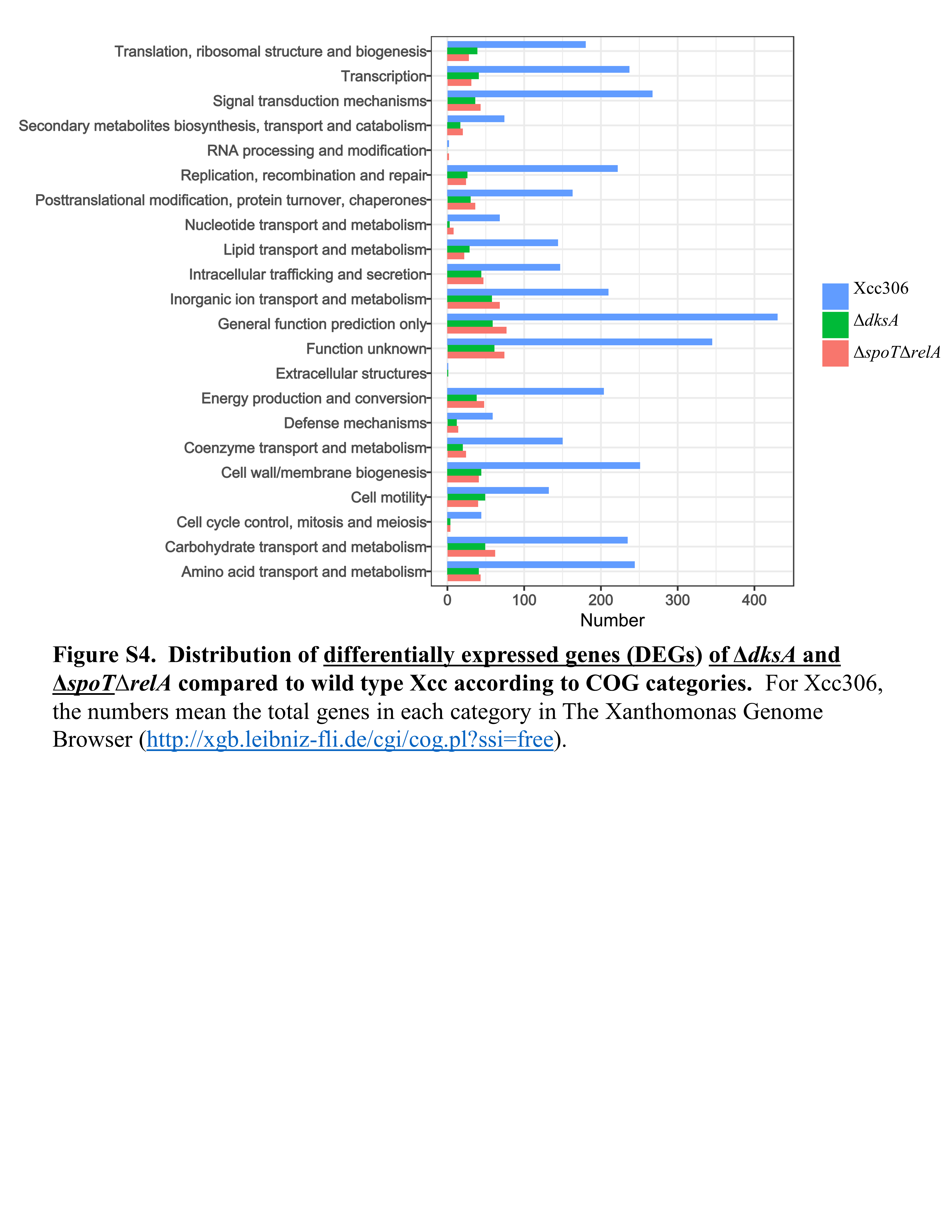

Supplement: Supplementary file 4 — Fig. S4 Distribution of DEGs of ΔdksA and ΔspoTΔrelA compared to wild‐type Xcc according to COG categories. For Xcc306, the numbers mean the total genes in each category according to the Xanthomonas Genome Browser (http://xgb.leibniz‐fli.de/cgi/cog.pl?ssi=free). [file MPP-20-1550-s004.TIF]

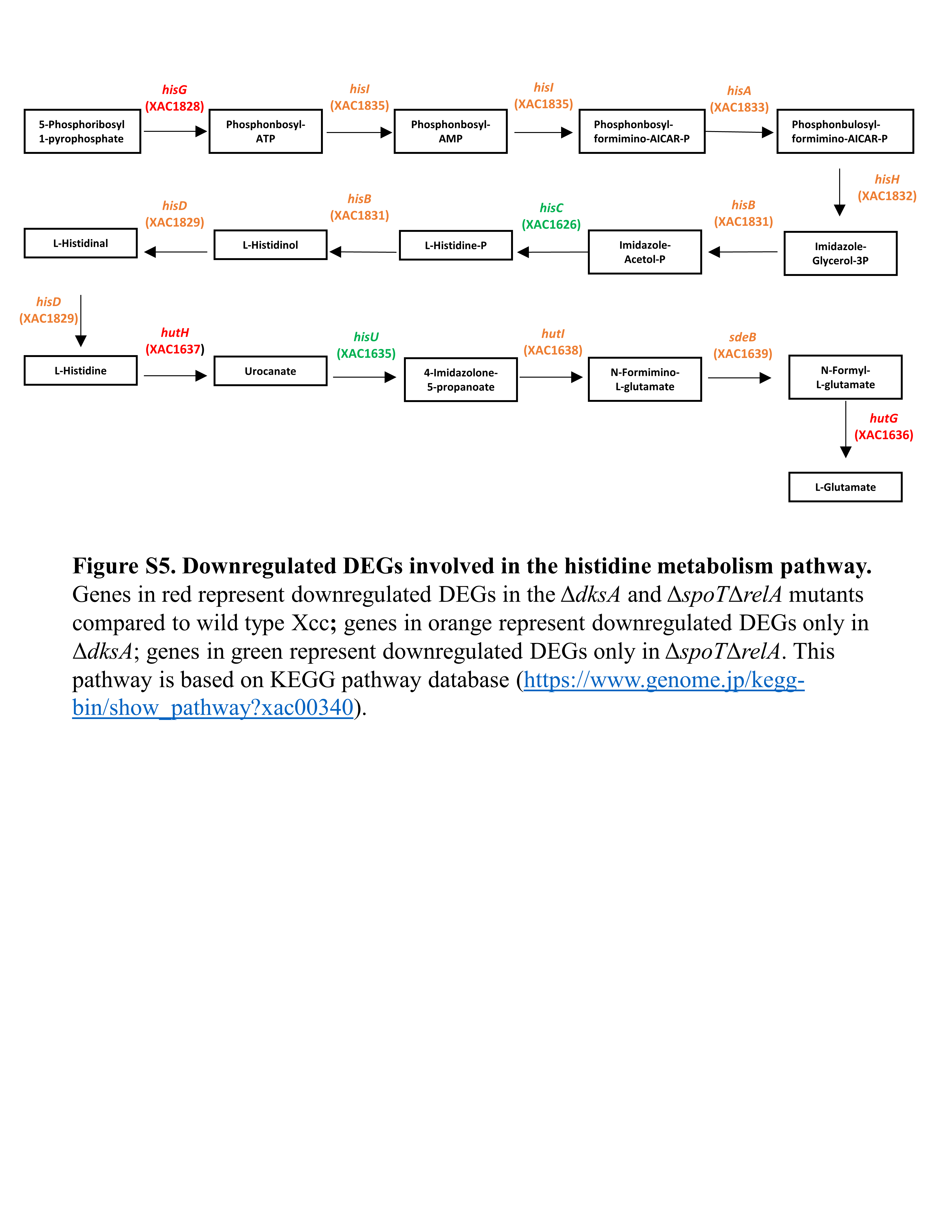

Supplement: Supplementary file 5 — Fig. S5 Down‐regulated DEGs involved in the histidine metabolism pathway. Genes in red represent down‐regulated DEGs in the ΔdksA and ΔspoTΔrelA mutants compared to wild‐type Xcc, genes in orange represent down‐regulated DEGs only in ΔdksA, genes in green represent down‐regulated DEGs only in ΔspoTΔrelA. This pathway is based on the KEGG pathway database (https://www.genome.jp/kegg‐bin/show_pathway?xac00340). [file MPP-20-1550-s005.TIF]

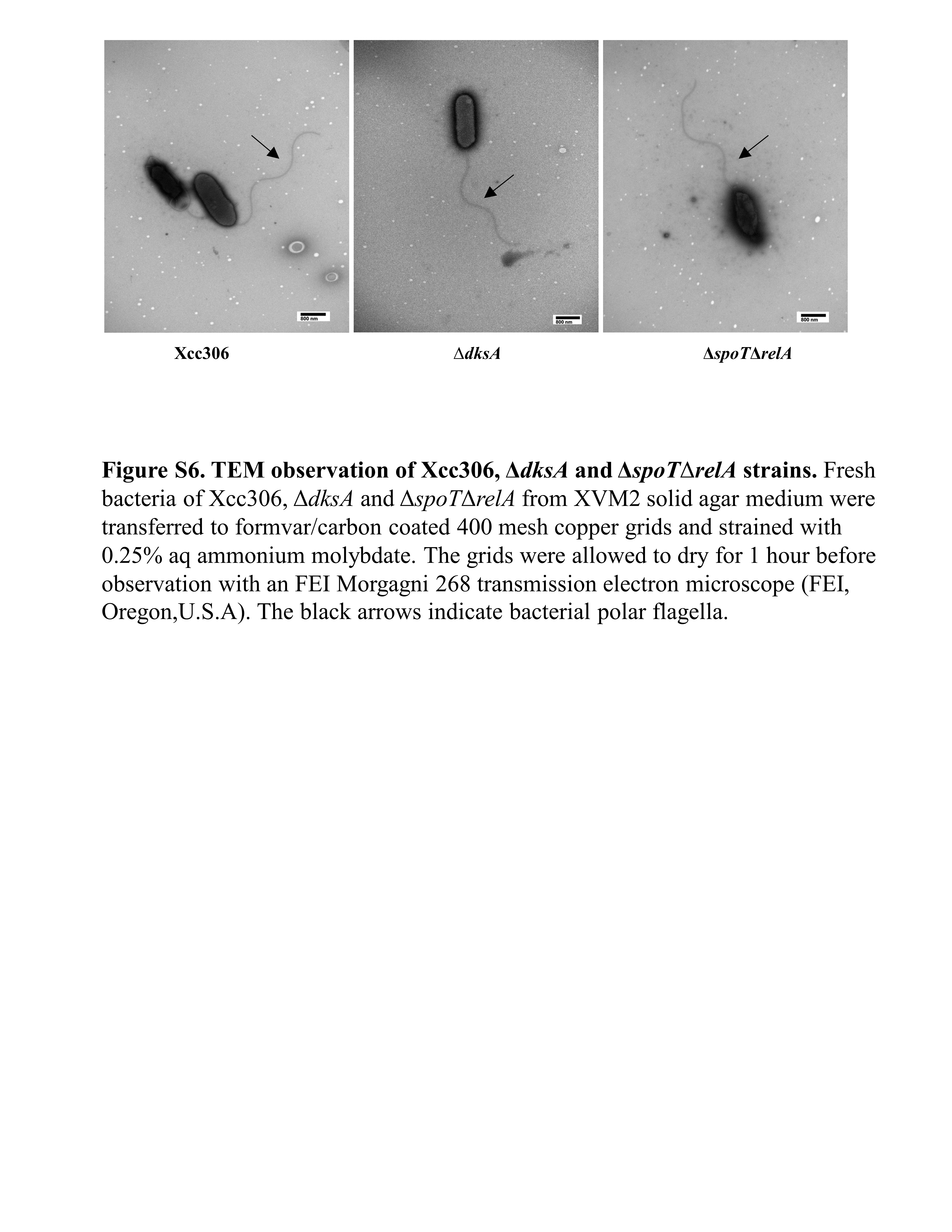

Supplement: Supplementary file 6 — Fig. S6 Transmission electron microscopic observation of Xcc306, ΔdksA and ΔspoTΔrelA strains. Fresh bacteria of Xcc306, ΔdksA and ΔspoTΔrelA from XVM2 solid agar medium were transferred to formvar/carbon‐coated 400‐mesh copper grids and stained with 0.25% aqueous ammonium molybdate. The grids were allowed to dry for 1 h before observation with an FEI Morgagni 268 transmission electron microscope (FEI, OR, USA). The black arrows indicate bacterial polar flagella. [file MPP-20-1550-s006.TIF]

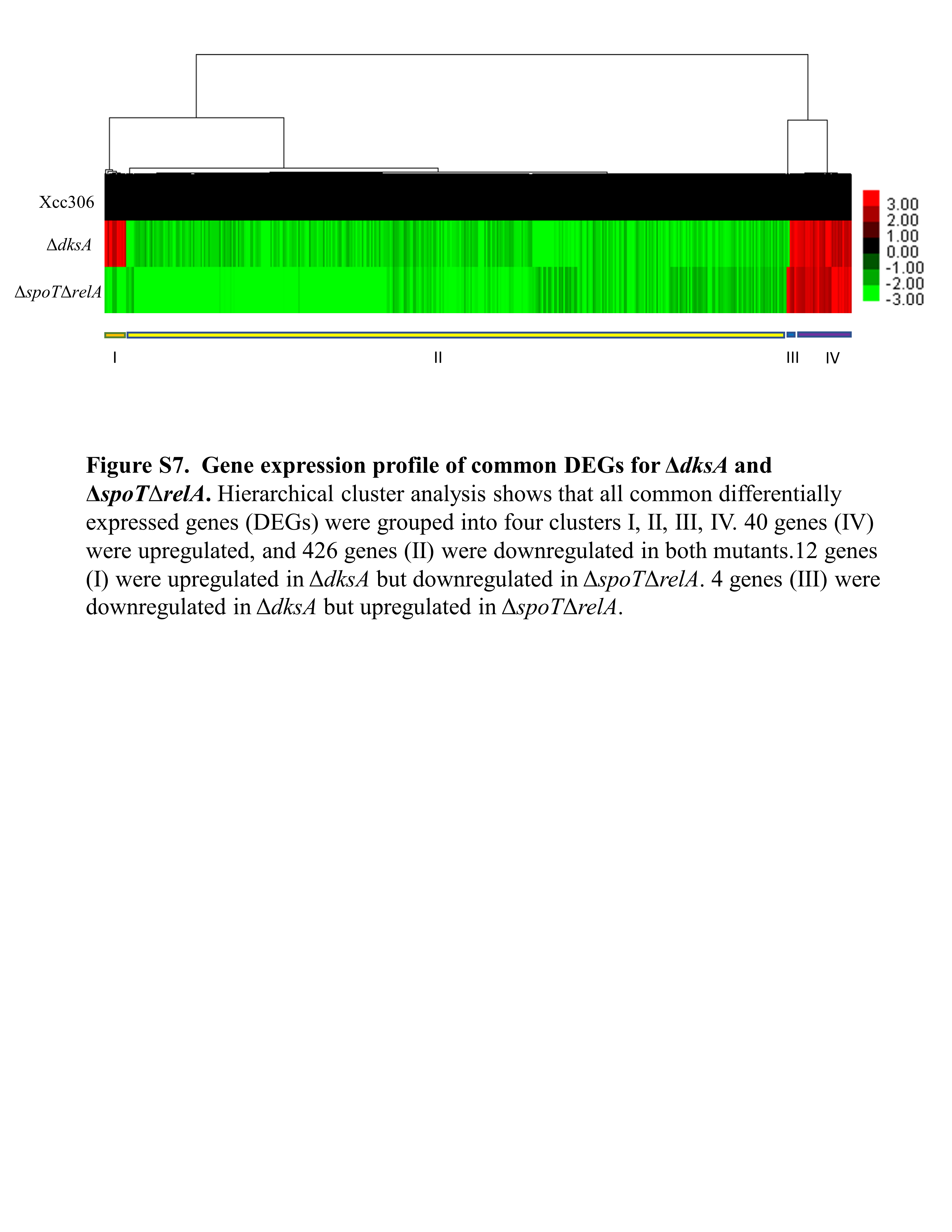

Supplement: Supplementary file 7 — Fig. S7 Gene expression profile of common DEGs for ΔdksA and ΔspoTΔrelA. Hierarchical cluster analysis shows that all common DEGs were grouped into four clusters: I, II, III and IV. Forty genes (IV) were up‐regulated, and 426 genes (II) were down‐regulated in both mutants. Twelve genes (I) were up‐regulated in ΔdksA but down‐regulated in ΔspoTΔrelA. Four genes (III) were down‐regulated in ΔdksA but up‐regulated in ΔspoTΔrelA. [file MPP-20-1550-s007.TIF]
