# Supplementary material for: Stringent response regulators (p)ppGpp and DksA positively regulate virulence and host adaptation of Xanthomonas citri
Source: Mol Plant Pathol. 2019 Oct 17;20(11):1550–65. doi: 10.1111/mpp.12865 (PMC6804348; doi:10.1111/mpp.12865)
Supplement: Supplementary file 8 — Table S1 Data quality summary. [file MPP-20-1550-s008.docx]

| **Sample** | **Raw reads** | **Clean reads** | **Raw base(G)** | **Clean base(G)** | **Effective rate (%)** | **Error rate (%)** | **Q20(%)** | **Q30(%)** | **GC content (%)** |
| --- | --- | --- | --- | --- | --- | --- | --- | --- | --- |
| w1 | 12248613 | 11887650 | 3.67 | 3.57 | 97.05 | 0.03 | 93.74 | 86.02 | 64.67 |
| w2 | 12975533 | 12513451 | 3.89 | 3.75 | 96.44 | 0.03 | 93.88 | 86.09 | 63.7 |
| w3 | 13026903 | 12399670 | 3.91 | 3.72 | 95.19 | 0.03 | 94.05 | 86.47 | 64.72 |
| A1 | 12855657 | 12429821 | 3.86 | 3.73 | 96.69 | 0.03 | 94.29 | 86.75 | 63.31 |
| A2 | 12355784 | 11942394 | 3.71 | 3.58 | 96.65 | 0.03 | 94.5 | 87.17 | 62.86 |
| A3 | 11238656 | 10926600 | 3.37 | 3.28 | 97.22 | 0.03 | 93.98 | 86.26 | 63.41 |
| AA1 | 12757861 | 12452748 | 3.83 | 3.74 | 97.61 | 0.03 | 94.12 | 86.61 | 63.57 |
| AA2 | 13331484 | 12997228 | 4 | 3.9 | 97.49 | 0.03 | 93.77 | 85.94 | 63.53 |
| AA3 | 12881446 | 12505742 | 3.86 | 3.75 | 97.08 | 0.03 | 93.84 | 86.09 | 63.35 |

**Table S1.** Data Quality Summary

w: wild-type Xcc306 A: ∆*dksA* AA: ∆*spoT/relA*; 1,2,3 means the biological repeats for each strain

Raw reads: total amount of reads of raw data.

Clean reads: total amount of reads of clean data.

Raw bases: (Raw reads) * (sequence length), calculated in G.

Clean bases: (Clean reads) * (sequence length), calculated in G.

Effective Rate (%): (Clean reads/Raw reads) *100%

Error rate: base error rate

Q20, Q30: (Base count of Phred value > 20 or 30) / (Total base count)

GC content: (G & C base count) / (Total base count)
