# Supplementary material for: Stringent response regulators (p)ppGpp and DksA positively regulate virulence and host adaptation of Xanthomonas citri
Source: Mol Plant Pathol. 2019 Oct 17;20(11):1550–65. doi: 10.1111/mpp.12865 (PMC6804348; doi:10.1111/mpp.12865)
Supplement: Supplementary file 9 — Table S2 Differentially regulated genes in the ΔspoTΔrelA mutant compared to the wild‐type Xcc. [file MPP-20-1550-s009.docx]

Table S2. Differentially regulated genes in the ΔspoTΔrelA mutant compared to the wild-type Xcc

| Name | Synonym | Product | log2 Fold change(∆spoT∆relA/WT) |
| --- | --- | --- | --- |
| - | XAC0035 | hypothetical protein | -2.72 |
| - | XAC0036 | hypothetical protein | -2.51 |
| - | XAC0071 | hypothetical protein | -2.70 |
| - | XAC0073 | hypothetical protein | -3.25 |
| cirA | XAC0074 | TonB-dependent receptor | -3.17 |
| xylR | XAC0075 | xylose repressor-like protein | -2.64 |
| avrBs2 | XAC0076 | avirulence protein | -2.72 |
| - | XAC0088 | NAD(P)H oxidoreductase | -2.04 |
| abiR | XAC0089 | abortive infection phage resistance protein | -2.19 |
| - | XAC0103 | hypothetical protein | -2.27 |
| - | XAC0107 | hypothetical protein | -2.97 |
| atsE | XAC0108 | AtsE protein | -4.00 |
| trxA | XAC0109 | thioredoxin | -3.70 |
| - | XAC0114 | hypothetical protein | -3.06 |
| - | XAC0115 | hypothetical protein | -3.50 |
| - | XAC0116 | hypothetical protein | -2.90 |
| - | XAC0131 | hypothetical protein | -3.15 |
| - | XAC0132 | hypothetical protein | -3.83 |
| - | XAC0137 | hypothetical protein | -3.49 |
| - | XAC0139 | hypothetical protein | -2.01 |
| - | XAC0145 | hypothetical protein | -2.32 |
| - | XAC0146 | hypothetical protein | -3.20 |
| - | XAC0149 | hypothetical protein | -6.07 |
| - | XAC0150 | hypothetical protein | -2.00 |
| estA1 | XAC0159 | carboxylesterase type B | -2.81 |
| xynB | XAC0160 | xylanase | -3.20 |
| dctP | XAC0162 | C4-dicarboxylate transport system | -2.42 |
| dctQ | XAC0163 | C4-dicarboxylate membrane transport protein | -2.22 |
| ygiK | XAC0164 | C4-dicarboxylate transport protein | -2.43 |
| - | XAC0165 | arabinosidase | -2.72 |
| - | XAC0171 | rhamnogalacturonan acetylesterase | -2.12 |
| - | XAC0172 | hypothetical protein | -3.70 |
| - | XAC0193 | hypothetical protein | -2.37 |
| glnA | XAC0204 | glutamine synthetase | -2.27 |
| sodC2 | XAC0210 | superoxide dismutase | -2.46 |
| lgtB | XAC0217 | glycosyltransferase | -2.70 |
| - | XAC0262 | dipeptidyl anminopeptidase | -2.15 |
| - | XAC0275 | hypothetical protein | -4.30 |
| - | XAC0276 | nitrile hydratase activator | -2.64 |
| hrpB | XAC0293 | ATP-dependent RNA helicase | -2.20 |
| - | XAC0295 | hypothetical protein | -2.83 |
| - | XAC0296 | monoxygenase | -2.56 |
| - | XAC0297 | hypothetical protein | -2.65 |
| - | XAC0298 | hypothetical protein | -2.30 |
| - | XAC0299 | hypothetical protein | -2.21 |
| - | XAC0300 | serine-pyruvate aminotransferase | -2.44 |
| amaB | XAC0301 | allantoate amidohydrolase | -4.00 |
| gatA | XAC0306 | amidase | -2.05 |
| yieG | XAC0309 | xanthine/uracil permease | -2.08 |
| vanB | XAC0310 | vanillate O-demethylase | -4.49 |
| vanA | XAC0311 | vanillate O-demethylase oxygenase | -4.21 |
| - | XAC0314 | hypothetical protein | -2.77 |
| - | XAC0315 | hypothetical protein | -2.32 |
| smeS | XAC0326 | two-component system sensor protein | -2.00 |
| smeA | XAC0327 | acriflavin resistance protein | -2.77 |
| smeB | XAC0328 | multidrug efflux transporter | -2.54 |
| smeC | XAC0329 | hypothetical protein | -2.05 |
| cmfA | XAC0330 | conditioned medium factor | -2.66 |
| sflA | XAC0334 | NADH-dependent FMN reductase | -5.07 |
| - | XAC0335 | hypothetical protein | -6.08 |
| metE | XAC0336 | 5-methyltetrahydropteroyltriglutamate--homocysteine S-methyltransferase | -5.27 |
| kdgT | XAC0337 | 2-keto-3-deoxygluconate permease | -4.20 |
| - | XAC0338 | hypothetical protein | -2.87 |
| int | XAC0344 | phage-related integrase | -3.64 |
| vanK | XAC0349 | MFS transporter | -2.18 |
| pobR | XAC0355 | PobR regulator | -2.19 |
| pobA | XAC0356 | 4-hydroxybenzoate 3-monooxygenase | -3.10 |
| pobB | XAC0362 | phenoxybenzoate dioxygenase subunit beta | -2.79 |
| gctA | XAC0364 | glutaconate CoA transferase subunit A | -2.36 |
| gctB | XAC0365 | glutaconate CoA transferase subunit B | -3.14 |
| pcaF | XAC0366 | beta-ketoadipyl CoA thiolase | -3.63 |
| pcaH | XAC0367 | protocatechuate 3,4-dioxygenase subunit beta | -2.10 |
| pcaG | XAC0368 | protocatechuate 3,4-dioxygenase subunit alpha | -2.58 |
| comF | XAC0389 | competence protein F | -3.17 |
| - | XAC0392 | hypothetical protein | -2.00 |
| hpaB | XAC0396 | HpaB protein | -2.17 |
| hrpE | XAC0397 | HrpE protein | -2.21 |
| hrpD5 | XAC0399 | HrpD5 protein | -2.42 |
| hpaA | XAC0400 | HpaA protein | -2.40 |
| hrcS | XAC0401 | HrcS protein | -4.98 |
| hrcR | XAC0402 | type III secretion system protein | -3.58 |
| hrcQ | XAC0403 | HrcQ protein | -2.66 |
| hpaP | XAC0404 | HpaP protein | -3.26 |
| hrcU | XAC0406 | type III secretion system protein HrcU | -4.61 |
| hrcJ | XAC0409 | HrcJ protein | -2.44 |
| hrpB4 | XAC0410 | HrpB4 protein | -2.24 |
| hrpB5 | XAC0411 | type III secretion system protein HrpB | -4.20 |
| hrcN | XAC0412 | type III secretion system ATPase | -2.95 |
| hrpB7 | XAC0413 | HrpB7 protein | -3.04 |
| hrcT | XAC0414 | HrcT protein | -3.17 |
| hpa1 | XAC0416 | Hpa1 protein | -4.38 |
| hpa2 | XAC0417 | Hpa2 protein | -2.58 |
| - | XAC0434 | hypothetical protein | -2.92 |
| oprN | XAC0436 | hypothetical protein | -2.55 |
| - | XAC0439 | cation efflux system protein | -2.77 |
| - | XAC0440 | short chain dehydrogenase | -2.74 |
| - | XAC0456 | hypothetical protein | -2.13 |
| - | XAC0469 | hypothetical protein | -4.25 |
| - | XAC0473 | hypothetical protein | -3.91 |
| - | XAC0500 | hypothetical protein | -2.81 |
| - | XAC0508 | LysR family transcriptional regulator | -2.21 |
| - | XAC0509 | MFS transporter | -4.46 |
| - | XAC0515 | hypothetical protein | -2.17 |
| - | XAC0516 | hypothetical protein | -3.25 |
| - | XAC0517 | hypothetical protein | -2.77 |
| - | XAC0518 | hypothetical protein | -2.66 |
| pgsA | XAC0519 | CDP-diacylglycerol--glycerol-3-phosphate 3-phosphatidyltransferase | -2.38 |
| - | XAC0525 | hypothetical protein | -3.44 |
| - | XAC0527 | hypothetical protein | -3.00 |
| - | XAC0529 | hypothetical protein | -2.27 |
| - | XAC0538 | hypothetical protein | -2.62 |
| aroG | XAC0545 | phospho-2-dehydro-3-deoxyheptonate aldolase | -2.31 |
| GNL | XAC0548 | gluconolactonase | -3.60 |
| - | XAC0549 | hypothetical protein | -3.30 |
| - | XAC0551 | hypothetical protein | -2.32 |
| appA | XAC0557 | 6-phytase | -4.09 |
| mdcH | XAC0566 | ACP S-malonyltransferase | -2.62 |
| matC | XAC0567 | dicarboxylate carrier protein | -2.89 |
| - | XAC0574 | hypothetical protein | -2.22 |
| - | XAC0587 | hypothetical protein | -2.10 |
| - | XAC0599 | hypothetical protein | -2.91 |
| - | XAC0605 | hypothetical protein | -2.77 |
| - | XAC0616 | hypothetical protein | -2.32 |
| - | XAC0617 | hypothetical protein | -3.04 |
| - | XAC0624 | hypothetical protein | -3.57 |
| moxJ | XAC0649 | MoxJ protein | -2.00 |
| - | XAC0650 | hypothetical protein | -3.10 |
| - | XAC0651 | surface antigen gene | -2.28 |
| adhC | XAC0652 | alcohol dehydrogenase | -3.77 |
| fepA | XAC0653 | TonB-dependent receptor | -2.54 |
| acoR | XAC0654 | transcriptional regulator AcoR | -3.25 |
| mreC | XAC0657 | rod shape-determining protein MreC | -2.18 |
| - | XAC0665 | hypothetical protein | -2.16 |
| - | XAC0682 | hypothetical protein | -2.82 |
| - | XAC0692 | hypothetical protein | -5.49 |
| fecA | XAC0693 | TonB-dependent receptor | -3.25 |
| xcsC | XAC0694 | type II secretion system protein C | -3.91 |
| xcsD | XAC0695 | type II secretion system protein D | -2.58 |
| xcsE | XAC0696 | type II secretion system protein E | -2.19 |
| xcsF | XAC0697 | type II secretion system protein F | -4.39 |
| xcsI | XAC0700 | type II secretion system protein I | -3.00 |
| xcsJ | XAC0701 | type II secretion system protein J | -2.84 |
| xcsL | XAC0703 | type II secretion system protein L | -2.36 |
| lacZ | XAC0707 | hypothetical protein | -2.25 |
| lacZ | XAC0708 | hypothetical protein | -3.62 |
| - | XAC0710 | hypothetical protein | -2.42 |
| betB | XAC0719 | betaine aldehyde dehydrogenase | -2.25 |
| - | XAC0737 | transcriptional regulator | -2.18 |
| - | XAC0738 | oxidoreductase | -2.58 |
| - | XAC0739 | hypothetical protein | -3.27 |
| - | XAC0740 | hypothetical protein | -3.09 |
| - | XAC0753 | hypothetical protein | -2.12 |
| - | XAC0754 | hypothetical protein | -3.04 |
| kdpB | XAC0757 | potassium-transporting ATPase subunit B | -2.42 |
| kdpC | XAC0758 | potassium-transporting ATPase subunit C | -2.52 |
| - | XAC0793 | hypothetical protein | -3.09 |
| - | XAC0802 | sulfotransferase | -3.22 |
| - | XAC0803 | methyltransferase | -3.51 |
| - | XAC0805 | hypothetical protein | -2.18 |
| - | XAC0810 | hypothetical protein | -2.27 |
| - | XAC0820 | hypothetical protein | -3.17 |
| - | XAC0824 | hypothetical protein | -2.97 |
| - | XAC0825 | hypothetical protein | -3.39 |
| nrtB | XAC0827 | permease | -2.87 |
| nrtCD | XAC0828 | ABC transporter ATP-binding protein | -2.22 |
| flbD | XAC0845 | transcriptional regulator | -3.64 |
| msuC | XAC0846 | FMNH2-dependent monooxygenase | -2.86 |
| ssuB | XAC0847 | ABC transporter ATP-binding protein | -2.70 |
| ssuC | XAC0848 | ABC transporter permease | -3.84 |
| ssuA | XAC0849 | sulfonate-binding protein | -2.91 |
| ssuD | XAC0850 | alkanesulfonate monooxygenase | -3.36 |
| slfA | XAC0851 | NADH-dependent FMN reductase | -2.86 |
| - | XAC0852 | TonB-dependent receptor | -3.39 |
| - | XAC0853 | hypothetical protein | -3.49 |
| - | XAC0854 | hypothetical protein | -4.25 |
| - | XAC0855 | monooxygenase | -2.96 |
| oppA | XAC0856 | ABC transporter oligopeptide-binding protein | -3.25 |
| oppB | XAC0857 | ABC transporter permease | -3.26 |
| oppC | XAC0858 | ABC transporter permease | -4.29 |
| - | XAC0859 | hypothetical protein | -3.26 |
| oppD | XAC0860 | ABC transporter ATP-binding protein | -4.22 |
| - | XAC0876 | hypothetical protein | -2.81 |
| - | XAC0881 | acyl-CoA synthetase | -3.22 |
| gst | XAC0894 | glutathione S-transferase | -2.63 |
| - | XAC0895 | hypothetical protein | -3.14 |
| - | XAC0916 | hydrolase | -2.60 |
| - | XAC0929 | extracellular protease | -2.72 |
| - | XAC0933 | hypothetical protein | -2.25 |
| - | XAC0934 | hypothetical protein | -3.00 |
| - | XAC1015 | hypothetical protein | -3.08 |
| fecA | XAC1023 | TonB-dependent receptor | -2.58 |
| - | XAC1024 | non-hemolytic phospholipase C | -2.81 |
| - | XAC1025 | hypothetical protein | -5.25 |
| - | XAC1026 | hypothetical protein | -4.25 |
| - | XAC1027 | hypothetical protein | -3.46 |
| - | XAC1037 | hypothetical protein | -2.45 |
| - | XAC1058 | hypothetical protein | -2.00 |
| gp19 | XAC1064 | phage-related DNA maturase | -3.25 |
| stf | XAC1068 | phage-related tail protein | -2.28 |
| pol | XAC1072 | phage-related DNA-directed RNA polymerase | -3.17 |
| opsX | XAC1094 | saccharide biosynthesis regulatory protein | -2.19 |
| - | XAC1105 | hypothetical protein | -2.00 |
| - | XAC1106 | hypothetical protein | -2.58 |
| prpR | XAC1136 | propionate catabolism regulatory protein | -2.18 |
| prpB | XAC1137 | 2-methylisocitrate lyase | -2.86 |
| - | XAC1149 | bacterioferritin | -2.12 |
| hspA | XAC1151 | low molecular weight heat shock protein | -3.64 |
| - | XAC1164 | hypothetical protein | -2.10 |
| - | XAC1165 | hypothetical protein | -2.62 |
| - | XAC1175 | hypothetical protein | -2.39 |
| - | XAC1180 | short chain dehydrogenase | -2.56 |
| - | XAC1193 | hypothetical protein | -3.39 |
| - | XAC1203 | hypothetical protein | -2.00 |
| hrpXct | XAC1266 | HrpX protein | -2.98 |
| - | XAC1304 | hypothetical protein | -2.43 |
| fadE9 | XAC1313 | acyl-CoA dehydrogenase | -2.37 |
| paaF | XAC1314 | enoyl-CoA hydratase | -2.40 |
| - | XAC1338 | oxidoreductase | -2.04 |
| - | XAC1346 | hypothetical protein | -2.50 |
| - | XAC1364 | hypothetical protein | -2.45 |
| acyII | XAC1436 | penicillin acylase II | -2.32 |
| cspA | XAC1465 | cold-shock protein | -3.53 |
| - | XAC1481 | dehydrogenase | -3.00 |
| mexE | XAC1482 | RND multidrug efflux membrane fusion protein | -3.13 |
| - | XAC1487 | hypothetical protein | -2.17 |
| int | XAC1498 | integrase | -2.81 |
| - | XAC1500 | hypothetical protein | -2.64 |
| - | XAC1512 | serine peptidase | -2.12 |
| hrcA | XAC1520 | heat-inducible transcription repressor | -2.66 |
| grpE | XAC1521 | heat shock protein GrpE | -2.46 |
| metH | XAC1559 | 5-methyltetrahydrofolate--homocysteine methyltransferase | -2.21 |
| - | XAC1563 | hypothetical protein | -3.70 |
| - | XAC1572 | hypothetical protein | -2.77 |
| pstB | XAC1574 | phosphate transporter ATP-binding protein | -2.64 |
| pstA | XAC1575 | ABC transporter phosphate permease | -2.47 |
| pstC | XAC1576 | ABC transporter phosphate permease | -2.97 |
| pstS | XAC1577 | phosphate ABC transporter substrate-binding protein | -2.34 |
| - | XAC1583 | hypothetical protein | -2.05 |
| hisC | XAC1626 | aminotransferase | -2.92 |
| hutU | XAC1635 | urocanate hydratase | -2.93 |
| hutG | XAC1636 | formylglutamate amidohydrolase | -2.98 |
| hutH | XAC1637 | histidine ammonia-lyase | -2.61 |
| repA | XAC1662 | hypothetical protein | -2.29 |
| - | XAC1663 | hypothetical protein | -3.25 |
| intA | XAC1664 | phage-related integrase | -2.31 |
| - | XAC1673 | hypothetical protein | -2.63 |
| cycL | XAC1674 | C-type cytochrome biogenesis protein | -2.58 |
| cycK | XAC1676 | C-type cytochrome biogenesis membrane protein | -2.66 |
| cycJ | XAC1677 | cytochrome c-type biogenesis protein CcmE | -2.26 |
| - | XAC1678 | hypothetical protein | -3.02 |
| ccmC | XAC1679 | cytochrome C-type biogenesis protein | -2.50 |
| - | XAC1680 | serine protease | -3.12 |
| - | XAC1681 | hypothetical protein | -3.46 |
| rpoE | XAC1682 | RNA polymerase sigma-E factor | -2.52 |
| - | XAC1683 | hypothetical protein | -3.17 |
| - | XAC1685 | cytochrome C | -3.32 |
| - | XAC1689 | hypothetical protein | -2.21 |
| - | XAC1690 | hypothetical protein | -3.17 |
| - | XAC1691 | aminotransferase | -3.12 |
| - | XAC1692 | lipopolysaccharide biosynthesis protein | -2.15 |
| - | XAC1693 | glycosyl transferase | -3.32 |
| - | XAC1694 | hypothetical protein | -2.94 |
| - | XAC1695 | hypothetical protein | -3.32 |
| - | XAC1696 | methyltransferase | -5.17 |
| - | XAC1697 | hypothetical protein | -2.91 |
| - | XAC1699 | glycosyltransferase | -2.64 |
| - | XAC1700 | hexosyltransferase | -4.17 |
| - | XAC1703 | hypothetical protein | -2.38 |
| - | XAC1704 | ABC transporter ATP-binding protein | -2.20 |
| celA | XAC1770 | cellulase | -2.09 |
| amiC | XAC1780 | N-acetylmuramoyl-L-alanine amidase | -3.75 |
| fhaC | XAC1814 | outer membrane hemolysin activator protein | -2.65 |
| fhaB | XAC1815 | filamentous hemagglutinin | -2.70 |
| tspO | XAC1819 | tryptophan-rich sensory protein | -3.74 |
| - | XAC1827 | hypothetical protein | -2.65 |
| hisG | XAC1828 | ATP phosphoribosyltransferase | -2.02 |
| - | XAC1873 | hypothetical protein | -3.17 |
| tsr | XAC1895 | chemotaxis protein | -2.87 |
| - | XAC1914 | hypothetical protein | -5.52 |
| - | XAC1918 | hemolysin-like protein | -2.65 |
| - | XAC1919 | hypothetical protein | -4.39 |
| - | XAC1924 | transposase | -3.81 |
| - | XAC1926 | hypothetical protein | -3.38 |
| - | XAC1928 | hypothetical protein | -3.09 |
| - | XAC1956 | hypothetical protein | -2.66 |
| - | XAC2009 | hypothetical protein | -2.58 |
| cirA | XAC2024 | TonB-dependent receptor | -3.09 |
| - | XAC2026 | hypothetical protein | -2.06 |
| - | XAC2029 | hypothetical protein | -2.27 |
| - | XAC2051 | oxidoreductase | -2.26 |
| syrE1 | XAC2097 | ATP-dependent serine activating enzyme | -2.00 |
| syrE2 | XAC2098 | ATP-dependent serine activating enzyme | -2.19 |
| - | XAC2113 | hypothetical protein | -2.52 |
| - | XAC2133 | oxidoreductase | -2.86 |
| - | XAC2140 | D-Ala-D-Ala carboxypeptidase | -2.50 |
| lytS | XAC2142 | two-component system sensor protein | -2.00 |
| czcA | XAC2147 | cation efflux system protein | -2.87 |
| yapH | XAC2151 | YapH protein | -4.04 |
| - | XAC2152 | hypothetical protein | -4.11 |
| cysG | XAC2157 | uroporphyrin-III C-methyltransferase | -2.07 |
| - | XAC2158 | histidine kinase-response regulator hybrid protein | -3.74 |
| - | XAC2160 | hypothetical protein | -2.58 |
| - | XAC2164 | hypothetical protein | -2.12 |
| - | XAC2172 | NADH dehydrogenase | -3.32 |
| - | XAC2173 | hypothetical protein | -2.62 |
| - | XAC2178 | hypothetical protein | -2.00 |
| - | XAC2190 | hypothetical protein | -2.66 |
| cirA | XAC2193 | TonB-dependent receptor | -2.17 |
| - | XAC2194 | hypothetical protein | -2.28 |
| - | XAC2197 | hemolysin-type calcium-binding protein | -3.23 |
| - | XAC2198 | hemolysin-type calcium-binding protein | -2.91 |
| - | XAC2199 | hypothetical protein | -4.86 |
| - | XAC2200 | hypothetical protein | -4.78 |
| hlyB | XAC2202 | hemolysin secretion protein B | -2.89 |
| - | XAC2208 | hypothetical protein | -5.04 |
| - | XAC2210 | hypothetical protein | -3.39 |
| ssb | XAC2211 | single-stranded DNA-binding protein | -3.86 |
| - | XAC2213 | cytosine-specific DNA methyltransferase | -3.54 |
| - | XAC2219 | hypothetical protein | -2.58 |
| - | XAC2220 | hypothetical protein | -3.58 |
| - | XAC2221 | hypothetical protein | -3.70 |
| int | XAC2222 | phage-related integrase | -2.20 |
| orf10 | XAC2242 | plasmid-like protein | -2.89 |
| orf8 | XAC2243 | plasmid-like protein | -2.39 |
| - | XAC2244 | hypothetical protein | -2.17 |
| - | XAC2248 | hypothetical protein | -4.17 |
| - | XAC2249 | hypothetical protein | -3.49 |
| - | XAC2250 | hypothetical protein | -2.44 |
| pilL | XAC2253 | PilL protein | -3.58 |
| - | XAC2254 | hypothetical protein | -4.25 |
| - | XAC2255 | hypothetical protein | -2.91 |
| - | XAC2256 | hypothetical protein | -3.94 |
| - | XAC2257 | hypothetical protein | -4.61 |
| - | XAC2258 | hypothetical protein | -5.17 |
| - | XAC2259 | hypothetical protein | -3.46 |
| - | XAC2260 | hypothetical protein | -4.00 |
| - | XAC2268 | hypothetical protein | -3.66 |
| - | XAC2269 | hypothetical protein | -5.36 |
| - | XAC2270 | hypothetical protein | -3.12 |
| - | XAC2271 | hypothetical protein | -3.87 |
| - | XAC2272 | hypothetical protein | -5.21 |
| - | XAC2274 | hypothetical protein | -3.13 |
| - | XAC2275 | hypothetical protein | -3.17 |
| nocR | XAC2276 | transcriptional regulator | -2.32 |
| - | XAC2277 | 3-hydroxyisobutyrate dehydrogenase | -4.09 |
| - | XAC2278 | hypothetical protein | -2.00 |
| - | XAC2279 | hypothetical protein | -2.68 |
| - | XAC2280 | hypothetical protein | -3.42 |
| - | XAC2281 | RadC family protein | -2.58 |
| - | XAC2282 | hypothetical protein | -4.25 |
| - | XAC2283 | hypothetical protein | -2.14 |
| - | XAC2284 | hypothetical protein | -3.46 |
| orf84 | XAC2285 | hypothetical protein | -3.39 |
| cydB | XAC2337 | cytochrome D ubiquinol oxidase subunit II | -3.44 |
| - | XAC2338 | hypothetical protein | -3.30 |
| - | XAC2339 | Pro tRNA | -5.00 |
| - | XAC2357 | hypothetical protein | -2.73 |
| eutC | XAC2366 | ethanolamine ammonia-lyase small subunit | -2.15 |
| - | XAC2371 | IS1479 transposase | -3.25 |
| - | XAC2402 | hypothetical protein | -2.10 |
| lig3 | XAC2414 | ATP-dependent DNA ligase | -3.48 |
| - | XAC2415 | hypothetical protein | -3.97 |
| kfrA | XAC2422 | plasmid-like protein | -2.51 |
| - | XAC2425 | hypothetical protein | -2.17 |
| mobB | XAC2440 | plasmid mobilization protein | -2.09 |
| repA | XAC2441 | replication protein | -2.15 |
| - | XAC2442 | hypothetical protein | -2.95 |
| gstA | XAC2460 | glutathione S-transferase | -2.26 |
| - | XAC2493 | two-component system regulatory protein | -2.05 |
| - | XAC2505 | hypothetical protein | -2.24 |
| - | XAC2506 | hypothetical protein | -2.43 |
| - | XAC2515 | AsnC family transcriptional regulator | -2.55 |
| - | XAC2517 | hypothetical protein | -4.09 |
| - | XAC2519 | hypothetical protein | -2.15 |
| cirA | XAC2520 | TonB-dependent receptor | -2.43 |
| btuB | XAC2531 | TonB-dependent receptor | -2.32 |
| xsa | XAC2533 | arabinosidase | -2.58 |
| - | XAC2534 | hypothetical protein | -3.75 |
| btuB | XAC2535 | TonB-dependent receptor | -2.46 |
| - | XAC2541 | peptidase | -2.51 |
| yveA | XAC2542 | amino acid permease | -2.77 |
| - | XAC2546 | ketoglutarate semialdehyde dehydrogenase | -2.08 |
| - | XAC2548 | oxidoreductase | -3.81 |
| - | XAC2549 | D-amino acid oxidase | -2.75 |
| - | XAC2550 | hypothetical protein | -2.58 |
| - | XAC2556 | hypothetical protein | -2.95 |
| cgt | XAC2596 | cyclomaltodextrin glucanotransferase | -2.58 |
| suc1 | XAC2597 | transporter | -2.42 |
| - | XAC2598 | hypothetical protein | -2.95 |
| aglA | XAC2599 | alpha-glucosidase | -4.42 |
| btuB | XAC2600 | TonB-dependent receptor | -2.98 |
| - | XAC2601 | hypothetical protein | -3.68 |
| aglA | XAC2602 | alpha-glucosidase | -4.48 |
| - | XAC2604 | ISxac4 transposase | -3.39 |
| - | XAC2629 | hypothetical protein | -3.54 |
| - | XAC2630 | hypothetical protein | -2.38 |
| - | XAC2631 | hypothetical protein | -2.09 |
| - | XAC2632 | hypothetical protein | -3.94 |
| - | XAC2637 | hypothetical protein | -5.36 |
| - | XAC2638 | hypothetical protein | -2.86 |
| - | XAC2639 | site-specific DNA-methyltransferase | -3.39 |
| Q | XAC2641 | phage-related capsid packaging protein | -2.81 |
| N | XAC2644 | phage-related major capsid protein | -3.58 |
| M | XAC2645 | phage-related terminase | -4.46 |
| L | XAC2646 | phage-related capsid completion protein | -4.00 |
| lys | XAC2650 | phage-related lytic protein | -4.52 |
| - | XAC2651 | hypothetical protein | -4.17 |
| R | XAC2652 | phage-related tail protein | -2.32 |
| S | XAC2653 | phage-related tail protein | -4.64 |
| - | XAC2654 | hypothetical protein | -3.32 |
| J | XAC2655 | phage-related baseplate assembly protein | -2.09 |
| I | XAC2656 | phage-related tail protein | -3.58 |
| - | XAC2657 | hypothetical protein | -2.74 |
| - | XAC2658 | hypothetical protein | -2.52 |
| - | XAC2659 | hypothetical protein | -2.27 |
| pilY1 | XAC2665 | PilY1 protein | -2.24 |
| - | XAC2733 | hypothetical protein | -2.84 |
| btuB | XAC2742 | TonB-dependent receptor | -2.25 |
| oar | XAC2743 | Oar protein | -2.83 |
| - | XAC2746 | metallopeptidase | -2.00 |
| - | XAC2763 | extracellular protease | -2.98 |
| - | XAC2774 | TonB-like protein | -3.32 |
| - | XAC2776 | hypothetical protein | -3.54 |
| - | XAC2785 | hypothetical protein | -4.04 |
| - | XAC2786 | hypothetical protein | -2.54 |
| - | XAC2794 | hypothetical protein | -2.06 |
| acr | XAC2799 | acriflavin resistance protein | -2.13 |
| mexC | XAC2800 | RND efflux membrane fusion protein | -3.55 |
| - | XAC2801 | hypothetical protein | -3.22 |
| ttgF | XAC2802 | outer membrane channel protein | -3.12 |
| - | XAC2815 | hypothetical protein | -2.44 |
| fhuA | XAC2830 | TonB-dependent receptor | -3.52 |
| - | XAC2831 | serine protease | -2.22 |
| - | XAC2832 | hypothetical protein | -2.81 |
| mocA | XAC2835 | oxidoreductase | -2.28 |
| - | XAC2836 | MFS transporter | -3.36 |
| araJ | XAC2837 | MFS transporter | -2.56 |
| - | XAC2841 | transcriptional regulator | -2.44 |
| mexB | XAC2843 | multidrug efflux transporter | -2.50 |
| - | XAC2851 | hypothetical protein | -2.00 |
| - | XAC2853 | cysteine protease | -4.82 |
| - | XAC2859 | hypothetical protein | -4.17 |
| - | XAC2860 | hypothetical protein | -2.32 |
| mcp | XAC2866 | chemotaxis protein | -2.22 |
| cheW | XAC2867 | chemotaxis protein | -3.58 |
| vieA | XAC2868 | response regulator | -2.72 |
| nfi | XAC2875 | endonuclease V | -2.15 |
| - | XAC2876 | hypothetical protein | -2.46 |
| - | XAC2920 | hypothetical protein | -2.02 |
| hrpW | XAC2922 | HrpW protein | -2.10 |
| proC | XAC2926 | pyrroline-5-carboxylate reductase | -2.03 |
| pfpI | XAC2932 | protease | -2.39 |
| - | XAC2944 | hypothetical protein | -2.26 |
| - | XAC2945 | hypothetical protein | -2.90 |
| - | XAC2946 | hypothetical protein | -2.81 |
| apbE | XAC2947 | thiamine biosynthesis lipoprotein ApbE | -2.58 |
| - | XAC2948 | sulfite reductase | -2.91 |
| qxtB | XAC2982 | quinol oxidase subunit II | -2.12 |
| - | XAC2984 | peptidase | -2.87 |
| - | XAC2992 | endoproteinase ArgC | -2.98 |
| - | XAC2996 | hypothetical protein | -2.58 |
| - | XAC2997 | hypothetical protein | -2.39 |
| fecA | XAC2998 | TonB-dependent receptor | -2.74 |
| soxR | XAC3000 | SoxR family transcriptional regulator | -2.51 |
| ptr | XAC3001 | MFS transporter | -4.58 |
| - | XAC3019 | hypothetical protein | -2.12 |
| - | XAC3021 | hypothetical protein | -2.81 |
| - | XAC3022 | hypothetical protein | -3.43 |
| - | XAC3023 | hypothetical protein | -3.32 |
| - | XAC3024 | hypothetical protein | -3.22 |
| - | XAC3025 | hypothetical protein | -2.35 |
| - | XAC3026 | transcriptional regulator | -2.96 |
| - | XAC3030 | hypothetical protein | -3.06 |
| - | XAC3038 | homoserine dehydrogenase | -2.86 |
| metB | XAC3039 | cystathionine gamma-synthase | -2.77 |
| metA | XAC3040 | homoserine O-acetyltransferase | -2.03 |
| fadE | XAC3054 | acyl-CoA dehydrogenase | -2.04 |
| iroN | XAC3071 | TonB-dependent receptor | -2.10 |
| fucA1 | XAC3072 | alpha-L-fucosidase | -3.46 |
| nahA | XAC3074 | beta-hexosaminidase | -2.00 |
| cirA | XAC3077 | TonB-dependent receptor | -3.17 |
| bga | XAC3078 | beta-galactosidase | -3.75 |
| yhfM | XAC3079 | cationic amino acid transporter | -3.25 |
| rbsK | XAC3080 | ribokinase | -5.49 |
| celF | XAC3081 | 6-phospho-beta-glucosidase | -4.13 |
| - | XAC3082 | hypothetical protein | -2.02 |
| - | XAC3085 | hypothetical protein | -3.43 |
| - | XAC3093 | hypothetical protein | -3.89 |
| - | XAC3094 | hypothetical protein | -2.32 |
| - | XAC3131 | hypothetical protein | -2.00 |
| - | XAC3152 | hypothetical protein | -2.91 |
| - | XAC3156 | hypothetical protein | -2.24 |
| fhuA | XAC3158 | TonB-dependent receptor | -2.50 |
| plcN | XAC3159 | phospholipase C | -3.17 |
| plcN | XAC3160 | phospholipase C | -3.09 |
| bioI | XAC3170 | cytochrome P-450 hydroxylase | -2.32 |
| mphE | XAC3175 | 4-hydroxy-2-oxovalerate aldolase | -3.00 |
| fecA | XAC3176 | citrate-dependent iron transporter | -2.81 |
| - | XAC3177 | hypothetical protein | -2.63 |
| - | XAC3178 | hypothetical protein | -5.39 |
| yceE | XAC3179 | transporter | -4.58 |
| iucA | XAC3180 | iron transporter | -3.51 |
| lysA | XAC3181 | diaminopimelate decarboxylase | -3.39 |
| clpB | XAC3195 | ATP-dependent Clp protease subunit | -3.10 |
| - | XAC3200 | nitrilotriacetate monooxygenase component A | -3.39 |
| fyuA | XAC3201 | TonB-dependent receptor | -3.22 |
| bfeA | XAC3207 | ferric enterobactin receptor | -3.19 |
| - | XAC3208 | hypothetical protein | -3.10 |
| orfS | XAC3227 | cointegrate resolution protein S | -3.25 |
| orfS | XAC3228 | cointegrate resolution protein S | -2.12 |
| - | XAC3233 | transposase | -2.70 |
| - | XAC3261 | hypothetical protein | -3.81 |
| - | XAC3275 | hypothetical protein | -2.22 |
| - | XAC3276 | hypothetical protein | -4.32 |
| - | XAC3282 | integrase | -2.32 |
| - | XAC3285 | hypothetical protein | -2.91 |
| - | XAC3289 | hypothetical protein | -2.64 |
| - | XAC3290 | hypothetical protein | -4.32 |
| - | XAC3291 | hypothetical protein | -4.73 |
| iroN | XAC3311 | TonB-dependent receptor | -2.20 |
| - | XAC3312 | glycosyl hydrolase | -3.27 |
| susB | XAC3313 | alpha-glucosidase | -2.00 |
| cysJ | XAC3330 | NADPH-sulfite reductase flavoprotein subunit | -2.94 |
| - | XAC3337 | hypothetical protein | -2.53 |
| cysG | XAC3340 | siroheme synthase | -4.52 |
| - | XAC3348 | hypothetical protein | -2.54 |
| - | XAC3349 | hypothetical protein | -2.58 |
| - | XAC3367 | hypothetical protein | -3.11 |
| - | XAC3368 | hypothetical protein | -3.63 |
| oar | XAC3418 | Oar protein | -2.87 |
| - | XAC3419 | Oar protein | -3.74 |
| hisC | XAC3423 | aminotransferase | -2.14 |
| leuB | XAC3456 | 3-isopropylmalate dehydrogenase | -2.07 |
| leuD | XAC3457 | isopropylmalate isomerase small subunit | -2.02 |
| - | XAC3460 | hypothetical protein | -2.97 |
| dctA | XAC3471 | C4-dicarboxylate transporter DctA | -2.32 |
| - | XAC3478 | hypothetical protein | -3.03 |
| - | XAC3479 | hypothetical protein | -2.77 |
| oprO | XAC3484 | porin | -4.58 |
| citM | XAC3485 | Mg++/citrate complex transporter | -3.13 |
| fabG | XAC3486 | 3-ketoacyl-ACP reductase | -3.58 |
| celS | XAC3507 | hypothetical protein | -2.97 |
| - | XAC3508 | hypothetical protein | -2.25 |
| - | XAC3509 | hypothetical protein | -3.32 |
| - | XAC3513 | hypothetical protein | -3.49 |
| - | XAC3514 | serine protease | -2.84 |
| bcsC | XAC3515 | cellulose synthase subunit C | -2.89 |
| - | XAC3516 | endo-1,4-D-glucanase | -2.83 |
| - | XAC3527 | hypothetical protein | -3.42 |
| - | XAC3545 | protease | -3.20 |
| xadA | XAC3546 | hypothetical protein | -2.93 |
| xadA | XAC3548 | hypothetical protein | -3.14 |
| - | XAC3552 | hypothetical protein | -2.29 |
| btuB | XAC3560 | TonB-dependent receptor | -2.83 |
| bioD | XAC3616 | dithiobiotin synthetase | -2.58 |
| ybjY | XAC3638 | ABC transporter permease | -4.52 |
| ycfV | XAC3639 | ABC transporter ATP-binding protein | -2.07 |
| ybjZ | XAC3640 | ABC transporter ATP-binding protein | -2.24 |
| - | XAC3676 | oxidoreductase | -2.28 |
| - | XAC3680 | hypothetical protein | -2.62 |
| - | XAC3685 | hypothetical protein | -2.09 |
| - | XAC3686 | hypothetical protein | -2.45 |
| - | XAC3690 | hypothetical protein | -2.73 |
| - | XAC3691 | methionine sulfoxide reductase B | -3.18 |
| - | XAC3692 | hypothetical protein | -3.15 |
| - | XAC3702 | hypothetical protein | -3.78 |
| - | XAC3706 | hypothetical protein | -3.09 |
| - | XAC3707 | hypothetical protein | -2.05 |
| wrbA | XAC3709 | tryptophan repressor-binding protein | -2.57 |
| - | XAC3715 | hypothetical protein | -3.23 |
| - | XAC3722 | hypothetical protein | -2.95 |
| - | XAC3724 | hypothetical protein | -2.17 |
| - | XAC3740 | UDP-glucose 4-epimerase | -2.06 |
| - | XAC3745 | hypothetical protein | -2.46 |
| - | XAC3746 | hypothetical protein | -2.41 |
| ybdR | XAC3747 | Zn-dependent alcohol dehydrogenase | -2.77 |
| - | XAC3757 | hypothetical protein | -2.84 |
| - | XAC3758 | hypothetical protein | -2.52 |
| - | XAC3760 | hypothetical protein | -3.00 |
| - | XAC3773 | acyl carrier phosphodiesterase | -2.32 |
| - | XAC3778 | hypothetical protein | -3.22 |
| - | XAC3866 | hypothetical protein | -2.80 |
| yliI | XAC3868 | dehydrogenase | -2.17 |
| ctaC | XAC3888 | cytochrome C oxidase subunit II | -2.38 |
| radC | XAC3915 | DNA repair protein RadC | -3.52 |
| ugt | XAC3921 | glucosyltransferase | -3.00 |
| entF | XAC3922 | ATP-dependent serine activating enzyme | -2.17 |
| - | XAC3938 | ISxac3 transposase | -2.22 |
| blc | XAC3956 | outer membrane lipoprotein Blc | -2.50 |
| - | XAC3966 | hypothetical protein | -3.30 |
| - | XAC3971 | hypothetical protein | -2.23 |
| - | XAC3976 | hypothetical protein | -3.13 |
| - | XAC3984 | hypothetical protein | -2.00 |
| - | XAC3999 | hypothetical protein | -2.44 |
| trpS | XAC4006 | tryptophanyl-tRNA synthetase | -2.16 |
| - | XAC4007 | hypothetical protein | -3.14 |
| - | XAC4027 | hypothetical protein | -4.07 |
| ankB | XAC4028 | ankyrin-like protein | -4.70 |
| - | XAC4037 | endonuclease | -3.58 |
| - | XAC4039 | hypothetical protein | -5.61 |
| - | XAC4043 | hypothetical protein | -2.27 |
| - | XAC4061 | hypothetical protein | -2.25 |
| fhuA | XAC4062 | TonB-dependent receptor | -2.58 |
| - | XAC4063 | hypothetical protein | -2.24 |
| ecaA | XAC4079 | a-type carbonic anhydrase | -2.58 |
| - | XAC4091 | hypothetical protein | -2.27 |
| - | XAC4105 | AMP-ligase | -2.58 |
| - | XAC4112 | hypothetical protein | -5.75 |
| yapH | XAC4113 | YapH protein | -2.58 |
| shlB | XAC4114 | hemolysin activator protein | -4.29 |
| - | XAC4115 | hypothetical protein | -4.36 |
| - | XAC4116 | serine/threonine kinase | -5.67 |
| - | XAC4118 | hypothetical protein | -6.15 |
| - | XAC4119 | hypothetical protein | -4.32 |
| - | XAC4120 | hypothetical protein | -4.83 |
| - | XAC4121 | hypothetical protein | -2.72 |
| - | XAC4122 | hypothetical protein | -4.25 |
| - | XAC4123 | hypothetical protein | -3.70 |
| - | XAC4125 | hypothetical protein | -3.32 |
| - | XAC4126 | hypothetical protein | -2.41 |
| pknB | XAC4127 | serine/threonine kinase | -2.08 |
| rpoE | XAC4129 | ECF sigma factor | -5.00 |
| - | XAC4130 | transmembrane sensor | -3.20 |
| - | XAC4131 | hypothetical protein | -4.58 |
| - | XAC4133 | hypothetical protein | -2.72 |
| - | XAC4134 | hypothetical protein | -2.95 |
| - | XAC4139 | hypothetical protein | -2.93 |
| clpB | XAC4140 | chaperone ClpB | -4.21 |
| - | XAC4141 | hypothetical protein | -5.46 |
| - | XAC4142 | hypothetical protein | -4.32 |
| - | XAC4143 | hypothetical protein | -3.91 |
| - | XAC4144 | hypothetical protein | -2.86 |
| fldZ | XAC4155 | hypothetical protein | -2.65 |
| fldA | XAC4156 | FldA protein | -4.64 |
| fldW | XAC4157 | 4-oxalomesaconate hydratase | -2.00 |
| czcA | XAC4160 | cation efflux system protein | -2.16 |
| czcB | XAC4161 | cation efflux system protein | -3.10 |
| czcC | XAC4162 | cation efflux system protein | -2.12 |
| phoD | XAC4166 | alkaline phosphatase | -3.29 |
| - | XAC4167 | hypothetical protein | -2.62 |
| - | XAC4173 | hypothetical protein | -3.61 |
| - | XAC4174 | peptidyl-prolyl cis-trans isomerase | -3.17 |
| - | XAC4175 | hypothetical protein | -2.58 |
| actP | XAC4176 | acetate permease | -2.10 |
| - | XAC4182 | cytochrome C biogenesis protein | -3.18 |
| - | XAC4183 | xylosidase | -3.10 |
| - | XAC4184 | oxidoreductase | -4.35 |
| - | XAC4185 | hypothetical protein | -5.88 |
| ucpA | XAC4186 | oxidoreductase | -3.00 |
| - | XAC4188 | RTS beta protein | -3.10 |
| - | XAC4189 | hypothetical protein | -2.06 |
| kdgR | XAC4191 | transcriptional regulator KdgR | -2.32 |
| ndvB | XAC4195 | NdvB protein | -2.43 |
| ynaJ | XAC4196 | cation symporter | -2.20 |
| - | XAC4205 | hypothetical protein | -3.97 |
| - | XAC4206 | hypothetical protein | -5.32 |
| aguA | XAC4227 | alpha-glucuronidase | -3.13 |
| - | XAC4228 | sialic acid-specific 9-O-acetylesterase | -4.06 |
| rspA | XAC4229 | starvation sensing protein | -2.91 |
| xylB | XAC4230 | arabinosidase | -3.46 |
| - | XAC4231 | glucan 1,4-beta-glucosidase | -3.39 |
| - | XAC4235 | hypothetical protein | -3.14 |
| alkH | XAC4238 | aldehyde dehydrogenase | -3.26 |
| - | XAC4239 | transmembrane protein | -3.94 |
| - | XAC4240 | hypothetical protein | -4.17 |
| hemL | XAC4241 | glutamate-1-semialdehyde aminotransferase | -2.95 |
| hemL | XAC4242 | glutamate-1-semialdehyde 2,1-aminomutase | -2.70 |
| fucA | XAC4243 | L-fuculose phosphate aldolase | -4.04 |
| xylB | XAC4244 | xylulose kinase | -3.17 |
| - | XAC4245 | hypothetical protein | -5.25 |
| - | XAC4246 | hypothetical protein | -5.49 |
| gnl | XAC4248 | gluconolactonase | -3.00 |
| exuT | XAC4255 | hexuranate transporter | -5.36 |
| cirA | XAC4256 | TonB-dependent receptor | -4.25 |
| xsa | XAC4258 | arabinosidase | -2.12 |
| blc | XAC4259 | lipocalin | -2.19 |
| - | XAC4261 | hypothetical protein | -2.42 |
| - | XAC4263 | hypothetical protein | -2.38 |
| - | XAC4273 | OmpA-like protein | -2.57 |
| benE | XAC4292 | benzoate transporter | -3.14 |
| - | XAC4296 | epimerase | -2.11 |
| - | XAC4298 | hypothetical protein | -3.32 |
| - | XAC4309 | phosphotransferase | -3.10 |
| cls | XAC4310 | cardiolipin synthetase | -2.42 |
| - | XAC4311 | hypothetical protein | -2.07 |
| - | XAC4321 | hypothetical protein | -3.37 |
| - | XAC4324 | hypothetical protein | -2.96 |
| - | XAC4329 | hypothetical protein | -3.22 |
| - | XAC4334 | hypothetical protein | -2.96 |
| - | XAC4338 | hypothetical protein | -2.10 |
| - | XAC4349 | alginate lyase | -3.94 |
| - | XAC4359 | sugar diacide regulator | -2.83 |
| glxK | XAC4360 | glycerate kinase | -3.87 |
| fecA | XAC4368 | TonB-dependent receptor | -2.58 |
| dnaA | XAC0001 | chromosome replication initiator DnaA | 2.61 |
| - | XAC0157 | hypothetical protein | 2.39 |
| - | XAC0318 | MerR family transcriptional regulator | 2.78 |
| glpK | XAC0358 | glycerol kinase | 2.49 |
| glpF | XAC0359 | glycerol uptake facilitator protein | 3.22 |
| glpD | XAC0360 | glycerol-3-phosphate dehydrogenase | 3.03 |
| mdoB | XAC0421 | phosphoglycerol transferase I | 2.71 |
| - | XAC0787 | peptidase | 2.11 |
| rplY | XAC0951 | 50S ribosomal protein L25 | 2 |
| - | XAC0958 | Trp tRNA | 2.58 |
| nusG | XAC0960 | transcription antitermination protein NusG | 2.26 |
| rplA | XAC0962 | 50S ribosomal protein L1 | 2 |
| rpsG | XAC0968 | 30S ribosomal protein S7 | 2.03 |
| rplC | XAC0972 | 50S ribosomal protein L3 | 2.55 |
| rplD | XAC0973 | 50S ribosomal protein L4 | 2.61 |
| rplB | XAC0975 | 50S ribosomal protein L2 | 2.14 |
| rpsS | XAC0976 | 30S ribosomal protein S19 | 2.38 |
| rplV | XAC0977 | 50S ribosomal protein L22 | 2.53 |
| rpsC | XAC0978 | 30S ribosomal protein S3 | 2.36 |
| rpsQ | XAC0981 | 30S ribosomal protein S17 | 2.48 |
| rplN | XAC0982 | 50S ribosomal protein L14 | 2.19 |
| rplX | XAC0983 | 50S ribosomal protein L24 | 2.16 |
| rpsN | XAC0985 | 30S ribosomal protein S14 | 2.84 |
| rplR | XAC0988 | 50S ribosomal protein L18 | 2.55 |
| rpsE | XAC0989 | 30S ribosomal protein S5 | 2.5 |
| rpmD | XAC0990 | 50S ribosomal protein L30 | 2.34 |
| rplO | XAC0991 | 50S ribosomal protein L15 | 2.57 |
| secY | XAC0992 | preprotein translocase subunit SecY | 2.8 |
| rpsD | XAC0995 | 30S ribosomal protein S4 | 2.15 |
| rpoA | XAC0996 | DNA-directed RNA polymerase subunit alpha | 2.33 |
| rplQ | XAC0997 | 50S ribosomal protein L17 | 2.18 |
| - | XAC1003 | hypothetical protein | 3.14 |
| typA | XAC1004 | GTP-binding elongation factor protein | 3.39 |
| tdh | XAC1022 | L-threonine 3-dehydrogenase | 2.08 |
| - | XAC1049 | Arg tRNA | 2.14 |
| - | XAC1050 | His tRNA | 2.53 |
| - | XAC1073 | Lys tRNA | 2.69 |
| - | XAC1082 | Val tRNA | 2.03 |
| - | XAC1083 | Asp tRNA | 2.95 |
| moaC | XAC1098 | molybdenum cofactor biosynthesis protein MoaC | 2.1 |
| lspA | XAC1255 | lipoprotein signal peptidase | 2.31 |
| rpsP | XAC1292 | 30S ribosomal protein S16 | 2.58 |
| rimM | XAC1293 | 16S rRNA-processing protein RimM | 2.46 |
| trmD | XAC1294 | tRNA (guanine-N(1)-)-methyltransferase | 2.23 |
| rplS | XAC1295 | 50S ribosomal protein L19 | 2.19 |
| yybA | XAC1443 | MarR family transcriptional regulator | 3.16 |
| yjcP | XAC1444 | outer membrane efflux protein | 2.46 |
| pmrA | XAC1445 | multidrug resistance efflux pump | 2.19 |
| pmrB | XAC1446 | multidrug resistance membrane translocase | 2.11 |
| - | XAC1452 | hypothetical protein | 2.02 |
| - | XAC1493 | transcriptional regulator | 2.74 |
| mobL | XAC1507 | plasmid mobilization protein | 2.56 |
| - | XAC1544 | hypothetical protein | 2.37 |
| - | XAC1545 | hypothetical protein | 3.3 |
| - | XAC1546 | hypothetical protein | 3.41 |
| nodI | XAC1547 | ABC transporter ATP-binding protein | 3.63 |
| - | XAC1548 | GntR family transcriptional regulator | 3.74 |
| rnt | XAC1571 | ribonuclease T | 2 |
| rpsF | XAC1620 | 30S ribosomal protein S6 | 2.37 |
| rpsR | XAC1621 | 30S ribosomal protein S18 | 2.26 |
| rplI | XAC1622 | 50S ribosomal protein L9 | 3.19 |
| - | XAC1634 | hypothetical protein | 2.34 |
| - | XAC1655 | transcriptional regulator | 2.01 |
| - | XAC1656 | Ser tRNA | 2.03 |
| lexA | XAC1739 | LexA repressor | 2.52 |
| - | XAC1745 | hypothetical protein | 2 |
| cheR | XAC1890 | chemotaxis protein methyltransferase | 2.02 |
| - | XAC1901 | hypothetical protein | 2.03 |
| cheY | XAC1904 | chemotaxis response regulator | 2.36 |
| - | XAC1905 | hypothetical protein | 2.6 |
| motB | XAC1908 | flagellar motor protein MotD | 2.18 |
| cheA | XAC1930 | chemotaxis protein | 3 |
| cheZ | XAC1931 | chemotaxis protein | 3.17 |
| cheY | XAC1932 | chemotaxis protein | 3.35 |
| fliA | XAC1933 | RNA polymerase sigma factor | 3 |
| fleN | XAC1934 | flagellar biosynthesis switch protein | 3.65 |
| flhA | XAC1936 | flagellar biosynthesis protein FlhA | 2.56 |
| flhB | XAC1937 | flagellar biosynthesis protein FlhB | 2.63 |
| - | XAC1940 | diguanylate cyclase | 2.33 |
| fliR | XAC1941 | flagellar biosynthetic protein | 3.09 |
| fliQ | XAC1942 | flagellar biosynthesis | 4.53 |
| fliP | XAC1944 | flagellar biosynthesis protein FliP | 3.34 |
| fliO | XAC1945 | flagellar protein | 2.84 |
| fliN | XAC1946 | flagellar protein | 3.85 |
| fliM | XAC1947 | flagellar motor switch protein FliM | 3.8 |
| fliL | XAC1948 | flagellar protein | 3.36 |
| fliJ | XAC1950 | flagellar FliJ protein | 2.21 |
| fliI | XAC1951 | flagellar protein | 2.18 |
| fliH | XAC1952 | flagellar protein | 2.33 |
| fliG | XAC1953 | flagellar protein | 2.44 |
| fliF | XAC1954 | flagellar MS-ring protein | 2.73 |
| fliE | XAC1955 | flagellar protein | 3.27 |
| fliS | XAC1973 | flagellar protein | 2.07 |
| fliD | XAC1974 | flagellar protein | 2.57 |
| fliC | XAC1975 | flagellin | 2.27 |
| flgL | XAC1976 | flagellar hook-associated protein FlgL | 3.72 |
| flgK | XAC1977 | flagellar hook-associated protein FlgK | 3.97 |
| flgJ | XAC1978 | flagellar rod assembly protein/muramidase FlgJ | 3.81 |
| flgI | XAC1979 | flagellar basal body P-ring biosynthesis protein FlgA | 3.82 |
| flgH | XAC1980 | flagellar basal body L-ring protein | 3.29 |
| flgG | XAC1981 | flagellar basal body rod protein FlgG | 3.66 |
| flgF | XAC1982 | flagellar basal body rod protein FlgF | 3.77 |
| flgE | XAC1983 | flagellar hook protein FlgE | 3.86 |
| flgD | XAC1984 | flagellar basal body rod modification protein | 3.95 |
| flgC | XAC1985 | flagellar basal body rod protein FlgC | 3.26 |
| flgB | XAC1986 | flagellar basal-body rod protein FlgB | 3.46 |
| flgA | XAC1988 | flagellar basal body P-ring biosynthesis protein FlgA | 2.75 |
| flgM | XAC1989 | flagellar protein | 2.56 |
| - | XAC1990 | hypothetical protein | 2.94 |
| - | XAC1993 | hypothetical protein | 2.61 |
| smf2 | XAC2036 | manganese transport protein MntH | 2.7 |
| pgsA | XAC2093 | CDP-diacylglycerol--glycerol-3-phosphate 3-phosphatidyltransferase | 2.03 |
| rne | XAC2111 | ribonuclease E | 2.43 |
| - | XAC2227 | hypothetical protein | 2.12 |
| ihfB | XAC2297 | integration host factor subunit beta | 2.34 |
| rpmJ | XAC2300 | 50S ribosomal protein L36 | 2.26 |
| - | XAC2316 | hypothetical protein | 2.01 |
| - | XAC2318 | pseudouridylate synthase | 2.06 |
| suhB | XAC2385 | extragenic supressor protein SuhB | 2.37 |
| cspA | XAC2395 | cold-shock protein | 2.75 |
| yacA | XAC2435 | plasmid-like protein | 2.15 |
| - | XAC2452 | hypothetical protein | 2.16 |
| - | XAC2467 | hypothetical protein | 2.32 |
| rrpX | XAC2482 | transcriptional regulator | 2.46 |
| pnp | XAC2683 | polynucleotide phosphorylase | 2.07 |
| rbfA | XAC2686 | ribosome-binding factor A | 2.71 |
| nusA | XAC2688 | transcription elongation factor NusA | 2.61 |
| - | XAC2689 | hypothetical protein | 3.1 |
| - | XAC2690 | Met tRNA | 3.09 |
| nuoA | XAC2704 | NADH dehydrogenase subunit A | 2.55 |
| - | XAC2705 | Leu tRNA | 2.27 |
| - | XAC2755 | hypothetical protein | 2.06 |
| - | XAC2812 | hypothetical protein | 2.04 |
| deaD | XAC2813 | ATP-dependent RNA helicase | 2.24 |
| - | XAC2827 | hypothetical protein | 2.12 |
| blaI | XAC3363 | BlaI family transcriptional regulator | 2.15 |
| - | XAC3503 | ISxac4 transposase | 2.1 |
| rhlE | XAC3610 | ATP-dependent RNA helicase | 2.98 |
| dadA | XAC3688 | D-amino acid dehydrogenase small subunit | 2.49 |
| - | XAC3732 | hypothetical protein | 2.26 |
| - | XAC3733 | NtrC family transcriptional regulator | 2.61 |
| - | XAC3749 | hypothetical protein | 3.84 |
| - | XAC3750 | hypothetical protein | 3.93 |
| - | XAC3783 | hypothetical protein | 3.08 |
| rho | XAC3831 | transcription termination factor Rho | 2.9 |
| - | XAC3963 | Ala tRNA | 6.65 |
| - | XAC3964 | hypothetical protein | 2.36 |
| - | XAC3965 | hypothetical protein | 2.07 |
| natB | XAC4053 | ABC transporter sodium permease | 3.08 |
| natA | XAC4054 | sodium ABC transporter ATP-binding protein | 3.23 |
| - | XAC4055 | cysteine proteinase | 3.38 |
| - | XAC4056 | transcriptional regulator | 4.19 |
| - | XAC4057 | hypothetical protein | 3.89 |
| trxA | XAC4072 | thioredoxin | 2.17 |
| - | XAC4073 | flavodoxin | 2.16 |
| nrdF | XAC4074 | ribonucleotide-diphosphate reductase subunit beta | 2.33 |
| - | XAC4268 | hypothetical protein | 2.54 |
| rnpA | XAC4373 | ribonuclease P | 2.05 |
| - | XACa0007 | hypothetical protein | 2.61 |
| parC | XACa0020 | partition protein C | 3.84 |
| repA | XACa0021 | replication protein A | 3.33 |
| tnpA | XACa0034 | Tn5045 transposase | 2.1 |
| tnpA | XACa0035 | Tn5045 transposase | 3.01 |
| - | XACa0036 | hypothetical protein | 3.04 |
| - | XACa0037 | hypothetical protein | 2.48 |
| - | XACb0010 | cointegrate resolution protein T | 2.05 |
| repA | XACb0016 | replication protein A | 3.33 |
| parC | XACb0017 | partition protein C | 3.84 |
| - | XACb0028 | hypothetical protein | 2.43 |
| - | XACb0034 | hypothetical protein | 2 |
| - | XACb0050 | ISxac2 transposase | 2.04 |
| - | XACb0051 | ISxac2 transposase | 2.87 |
| - | XACb0052 | partition gene repressor | 3.24 |
| parB | XACb0053 | partition protein B | 3.35 |
| parA | XACb0054 | partition protein A | 3.72 |
| tnpA | XACb0067 | Tn5045 transposase | 2.11 |
| tnpA | XACb0068 | Tn5045 transposase | 3.01 |
| - | XACb0069 | hypothetical protein | 3.04 |
| - | XACb0070 | hypothetical protein | 2.48 |
