# Supplementary material for: Stringent response regulators (p)ppGpp and DksA positively regulate virulence and host adaptation of Xanthomonas citri
Source: Mol Plant Pathol. 2019 Oct 17;20(11):1550–65. doi: 10.1111/mpp.12865 (PMC6804348; doi:10.1111/mpp.12865)
Supplement: Supplementary file 10 — Table S3 Differentially regulated genes in the ΔdksA mutant compared to wild‐type (WT) Xcc. [file MPP-20-1550-s010.docx]

Table S3. Differentially regulated genes in the ∆dksA mutant compared to wild type (WT) Xcc

| *Name* | Synonym | Product | log2 Foldchange (∆dksA/WT) |
| --- | --- | --- | --- |
| *-* | XAC0027 | hypothetical protein | -2.51 |
| *-* | XAC0035 | hypothetical protein | -2.14 |
| *-* | XAC0037 | penicillin acylase | -2.32 |
| *-* | XAC0056 | polysaccharide export protein | -2.18 |
| *-* | XAC0085 | hypothetical protein | -2.58 |
| *-* | XAC0093 | ISxac1 transposase | -2.06 |
| *-* | XAC0099 | hypothetical protein | -2.17 |
| *-* | XAC0103 | hypothetical protein | -2.44 |
| *-* | XAC0104 | metalloprotease | -2.23 |
| *atsE* | XAC0108 | AtsE protein | -4.43 |
| *trxA* | XAC0109 | thioredoxin | -4.28 |
| *-* | XAC0114 | hypothetical protein | -2.14 |
| *-* | XAC0115 | hypothetical protein | -2.30 |
| *-* | XAC0116 | hypothetical protein | -2.36 |
| *-* | XAC0131 | hypothetical protein | -2.43 |
| *-* | XAC0132 | hypothetical protein | -2.69 |
| *-* | XAC0145 | hypothetical protein | -2.06 |
| *-* | XAC0148 | ISxac1 transposase | -2.06 |
| *-* | XAC0149 | hypothetical protein | -2.90 |
| *-* | XAC0154 | alpha-amylase | -2.13 |
| *estA1* | XAC0159 | carboxylesterase type B | -2.58 |
| *xynB* | XAC0160 | xylanase | -2.46 |
| *dctP* | XAC0162 | C4-dicarboxylate transport system | -2.42 |
| *ygiK* | XAC0164 | C4-dicarboxylate transport protein | -2.43 |
| *-* | XAC0165 | arabinosidase | -2.24 |
| *-* | XAC0172 | hypothetical protein | -2.24 |
| *glnA* | XAC0204 | glutamine synthetase | -2.41 |
| *rbcR* | XAC0255 | transcriptional regulator | -2.22 |
| *mls* | XAC0256 | malate synthase | -2.43 |
| *aceA* | XAC0257 | isocitrate lyase | -2.75 |
| *-* | XAC0275 | hypothetical protein | -2.72 |
| *avrXacE1* | XAC0286 | avirulence protein | -2.66 |
| *-* | XAC0296 | monoxygenase | -2.22 |
| *-* | XAC0297 | hypothetical protein | -2.79 |
| *-* | XAC0298 | hypothetical protein | -2.43 |
| *-* | XAC0299 | hypothetical protein | -2.47 |
| *-* | XAC0300 | serine-pyruvate aminotransferase | -2.34 |
| *amaB* | XAC0301 | allantoate amidohydrolase | -2.68 |
| *opdE* | XAC0303 | transcriptional regulator | -2.43 |
| *vanB* | XAC0310 | vanillate O-demethylase | -2.91 |
| *vanA* | XAC0311 | vanillate O-demethylase oxygenase | -3.21 |
| *-* | XAC0314 | hypothetical protein | -2.36 |
| *ynfM* | XAC0317 | MFS transporter | -2.14 |
| *-* | XAC0319 | chloroperoxidase | -2.66 |
| *smeA* | XAC0327 | acriflavin resistance protein | -2.77 |
| *smeB* | XAC0328 | multidrug efflux transporter | -2.81 |
| *smeC* | XAC0329 | hypothetical protein | -2.54 |
| *kdgT* | XAC0337 | 2-keto-3-deoxygluconate permease | -2.78 |
| *-* | XAC0338 | hypothetical protein | -2.35 |
| *int* | XAC0344 | phage-related integrase | -2.64 |
| *vanK* | XAC0349 | MFS transporter | -2.71 |
| *pobA* | XAC0356 | 4-hydroxybenzoate 3-monooxygenase | -2.43 |
| *-* | XAC0357 | hypothetical protein | -2.42 |
| *catD* | XAC0370 | b-ketoadipate enol-lactone hydrolase | -2.26 |
| *pcaC* | XAC0371 | 4-carboxymuconolactone decarboxylase | -2.09 |
| *hrpF* | XAC0394 | HrpF protein | -2.58 |
| *-* | XAC0395 | hypothetical protein | -2.91 |
| *hpaB* | XAC0396 | HpaB protein | -2.49 |
| *hrpD5* | XAC0399 | HrpD5 protein | -2.42 |
| *hpaA* | XAC0400 | HpaA protein | -2.40 |
| *hrcS* | XAC0401 | HrcS protein | -3.17 |
| *hrcR* | XAC0402 | type III secretion system protein | -3.00 |
| *hrcQ* | XAC0403 | HrcQ protein | -2.66 |
| *hpaP* | XAC0404 | HpaP protein | -2.78 |
| *hrcV* | XAC0405 | HrcV protein | -2.91 |
| *hrcU* | XAC0406 | type III secretion system protein HrcU | -3.03 |
| *hrpB1* | XAC0407 | HrpB1 protein | -2.45 |
| *hrcJ* | XAC0409 | HrcJ protein | -2.66 |
| *hrpB4* | XAC0410 | HrpB4 protein | -2.69 |
| *hrpB5* | XAC0411 | type III secretion system protein HrpB | -2.78 |
| *hrcN* | XAC0412 | type III secretion system ATPase | -3.17 |
| *hrpB7* | XAC0413 | HrpB7 protein | -3.04 |
| *hrcT* | XAC0414 | HrcT protein | -3.17 |
| *hrcC* | XAC0415 | HrcC protein | -2.74 |
| *hpa1* | XAC0416 | Hpa1 protein | -3.21 |
| *hpa2* | XAC0417 | Hpa2 protein | -3.58 |
| *-* | XAC0418 | hypothetical protein | -3.00 |
| *-* | XAC0419 | hypothetical protein | -2.46 |
| *virK* | XAC0435 | VirK protein | -2.00 |
| *mexE* | XAC0438 | component of multidrug efflux system | -2.25 |
| *-* | XAC0439 | cation efflux system protein | -2.77 |
| *-* | XAC0440 | short chain dehydrogenase | -2.74 |
| *-* | XAC0456 | hypothetical protein | -2.32 |
| *-* | XAC0469 | hypothetical protein | -2.66 |
| *-* | XAC0473 | hypothetical protein | -2.58 |
| *-* | XAC0500 | hypothetical protein | -2.39 |
| *-* | XAC0502 | ISxac1 transposase | -2.03 |
| *-* | XAC0509 | MFS transporter | -2.58 |
| *-* | XAC0515 | hypothetical protein | -2.17 |
| *-* | XAC0516 | hypothetical protein | -2.66 |
| *-* | XAC0517 | hypothetical protein | -2.50 |
| *-* | XAC0518 | hypothetical protein | -2.39 |
| *pgsA* | XAC0519 | CDP-diacylglycerol--glycerol-3-phosphate 3-phosphatidyltransferase | -2.49 |
| *-* | XAC0525 | hypothetical protein | -2.85 |
| *-* | XAC0543 | hypothetical protein | -2.47 |
| *GNL* | XAC0548 | gluconolactonase | -3.22 |
| *-* | XAC0549 | hypothetical protein | -2.91 |
| *appA* | XAC0557 | 6-phytase | -2.50 |
| *-* | XAC0599 | hypothetical protein | -3.32 |
| *-* | XAC0606 | endonuclease | -2.56 |
| *-* | XAC0607 | hypothetical protein | -3.65 |
| *-* | XAC0616 | hypothetical protein | -2.51 |
| *-* | XAC0617 | hypothetical protein | -2.72 |
| *-* | XAC0624 | hypothetical protein | -2.67 |
| *-* | XAC0629 | hypothetical protein | -2.32 |
| *ptrB* | XAC0631 | oligopeptidase B | -2.56 |
| *hslV* | XAC0637 | ATP-dependent protease peptidase subunit | -5.17 |
| *hslU* | XAC0638 | ATP-dependent protease ATP-binding subunit HslU | -5.26 |
| *mxaF* | XAC0648 | methanol dehydrogenase heavy chain | -2.48 |
| *moxJ* | XAC0649 | MoxJ protein | -2.85 |
| *-* | XAC0650 | hypothetical protein | -2.32 |
| *-* | XAC0651 | surface antigen gene | -2.09 |
| *adhC* | XAC0652 | alcohol dehydrogenase | -2.55 |
| *acoR* | XAC0654 | transcriptional regulator AcoR | -2.08 |
| *prc* | XAC0669 | tail-specific protease | -2.77 |
| *-* | XAC0670 | hypothetical protein | -2.70 |
| *-* | XAC0679 | hypothetical protein | -2.29 |
| *gndA* | XAC0680 | 6-phosphogluconate dehydrogenase | -2.15 |
| *-* | XAC0692 | hypothetical protein | -2.91 |
| *fecA* | XAC0693 | TonB-dependent receptor | -3.25 |
| *xcsC* | XAC0694 | type II secretion system protein C | -2.58 |
| *xcsD* | XAC0695 | type II secretion system protein D | -2.91 |
| *xcsE* | XAC0696 | type II secretion system protein E | -3.00 |
| *xcsF* | XAC0697 | type II secretion system protein F | -3.07 |
| *xcsG* | XAC0698 | type II secretion system protein G | -2.94 |
| *xcsH* | XAC0699 | type II secretion system protein H | -3.25 |
| *xcsI* | XAC0700 | type II secretion system protein I | -3.19 |
| *xcsJ* | XAC0701 | type II secretion system protein J | -3.10 |
| *xcsK* | XAC0702 | type II secretion system protein K | -2.89 |
| *xcsL* | XAC0703 | type II secretion system protein L | -2.85 |
| *xcsN* | XAC0705 | type II secretion system protein N | -2.39 |
| *lacZ* | XAC0707 | hypothetical protein | -2.66 |
| *lacZ* | XAC0708 | hypothetical protein | -2.89 |
| *-* | XAC0709 | N-acetylglucosaminidase | -2.58 |
| *-* | XAC0710 | hypothetical protein | -3.00 |
| *-* | XAC0732 | hypothetical protein | -2.02 |
| *-* | XAC0737 | transcriptional regulator | -2.56 |
| *-* | XAC0738 | oxidoreductase | -2.58 |
| *-* | XAC0739 | hypothetical protein | -3.27 |
| *-* | XAC0742 | hypothetical protein | -2.02 |
| *-* | XAC0753 | hypothetical protein | -2.48 |
| *-* | XAC0754 | hypothetical protein | -2.77 |
| *-* | XAC0786 | hypothetical protein | -2.36 |
| *-* | XAC0792 | hypothetical protein | -2.20 |
| *-* | XAC0793 | hypothetical protein | -2.50 |
| *-* | XAC0796 | hypothetical protein | -2.62 |
| *-* | XAC0803 | methyltransferase | -2.51 |
| *-* | XAC0810 | hypothetical protein | -2.27 |
| *cirA* | XAC0811 | TonB-dependent receptor | -2.81 |
| *appA* | XAC0812 | phosphoanhydride phosphohydrolase | -2.48 |
| *-* | XAC0820 | hypothetical protein | -3.17 |
| *-* | XAC0831 | oxidoreductase | -2.61 |
| *ftsE* | XAC0832 | ABC transporter ATP-binding protein | -2.79 |
| *msuC* | XAC0846 | FMNH2-dependent monooxygenase | -2.05 |
| *-* | XAC0852 | TonB-dependent receptor | -2.81 |
| *-* | XAC0853 | hypothetical protein | -2.64 |
| *-* | XAC0854 | hypothetical protein | -2.37 |
| *-* | XAC0855 | monooxygenase | -2.12 |
| *oppA* | XAC0856 | ABC transporter oligopeptide-binding protein | -2.44 |
| *oppB* | XAC0857 | ABC transporter permease | -2.58 |
| *oppC* | XAC0858 | ABC transporter permease | -2.58 |
| *-* | XAC0859 | hypothetical protein | -2.58 |
| *oppD* | XAC0860 | ABC transporter ATP-binding protein | -2.81 |
| *-* | XAC0874 | hydrolase | -2.22 |
| *-* | XAC0875 | hypothetical protein | -2.12 |
| *-* | XAC0876 | hypothetical protein | -3.22 |
| *-* | XAC0899 | hypothetical protein | -2.39 |
| *pms* | XAC0900 | methionine sulfoxide reductase A | -4.27 |
| *talB* | XAC0902 | transaldolase B | -2.22 |
| *rnk* | XAC0903 | regulator of nucleoside diphosphate kinase | -3.25 |
| *-* | XAC0904 | hypothetical protein | -3.02 |
| *-* | XAC0916 | hydrolase | -2.19 |
| *-* | XAC0929 | extracellular protease | -2.24 |
| *-* | XAC0934 | hypothetical protein | -2.42 |
| *cirA* | XAC0999 | colicin I receptor | -2.46 |
| *fecA* | XAC1023 | TonB-dependent receptor | -2.58 |
| *-* | XAC1024 | non-hemolytic phospholipase C | -2.49 |
| *-* | XAC1025 | hypothetical protein | -3.25 |
| *-* | XAC1026 | hypothetical protein | -3.25 |
| *-* | XAC1027 | hypothetical protein | -3.04 |
| *-* | XAC1037 | hypothetical protein | -3.10 |
| *gtrB* | XAC1038 | glycosyl transferase | -2.42 |
| *-* | XAC1043 | hypothetical protein | -2.15 |
| *gp19* | XAC1064 | phage-related DNA maturase | -3.25 |
| *stf* | XAC1068 | phage-related tail protein | -2.09 |
| *pol* | XAC1072 | phage-related DNA-directed RNA polymerase | -2.17 |
| *-* | XAC1125 | hypothetical protein | -2.42 |
| *fecA* | XAC1146 | TonB-dependent receptor | -2.70 |
| *hspA* | XAC1151 | low molecular weight heat shock protein | -4.15 |
| *-* | XAC1157 | hypothetical protein | -2.22 |
| *-* | XAC1169 | hypothetical protein | -2.08 |
| *stkXac1* | XAC1171 | serine/threonine kinase | -2.32 |
| *-* | XAC1172 | hypothetical protein | -2.48 |
| *-* | XAC1177 | hypothetical protein | -4.12 |
| *-* | XAC1178 | oxidoreductase | -4.20 |
| *-* | XAC1180 | short chain dehydrogenase | -3.74 |
| *-* | XAC1181 | hypothetical protein | -3.37 |
| *-* | XAC1187 | hydroxylase large subunit | -3.06 |
| *-* | XAC1188 | hydroxylase molybdopterin-containing subunit | -3.23 |
| *-* | XAC1189 | ferredoxin | -2.85 |
| *tcmJ* | XAC1192 | tetracenomycin polyketide synthesis protein | -2.16 |
| *-* | XAC1198 | hypothetical protein | -2.07 |
| *-* | XAC1208 | hypothetical protein | -2.27 |
| *-* | XAC1209 | hypothetical protein | -2.81 |
| *-* | XAC1210 | hypothetical protein | -4.11 |
| *katE* | XAC1211 | catalase | -3.08 |
| *yjdB* | XAC1218 | hypothetical protein | -4.37 |
| *-* | XAC1219 | hypothetical protein | -5.02 |
| *pthX* | XAC1242 | pathogenicity-like protein | -3.56 |
| *hslR* | XAC1300 | heat shock protein 15-like protein | -3.01 |
| *-* | XAC1304 | hypothetical protein | -2.43 |
| *algU* | XAC1319 | RNA polymerase sigma factor RpoE | -3.80 |
| *-* | XAC1320 | regulatory protein | -3.57 |
| *mucD* | XAC1321 | periplasmic protease | -3.38 |
| *-* | XAC1338 | oxidoreductase | -2.46 |
| *lhr2* | XAC1340 | helicase-like protein | -2.29 |
| *lig2* | XAC1341 | ATP-dependent DNA ligase | -2.51 |
| *-* | XAC1343 | hypothetical protein | -2.16 |
| *-* | XAC1355 | hypothetical protein | -2.12 |
| *-* | XAC1361 | hypothetical protein | -2.54 |
| *nerA* | XAC1362 | GTN reductase | -3.09 |
| *-* | XAC1364 | hypothetical protein | -4.05 |
| *-* | XAC1382 | hypothetical protein | -2.81 |
| *-* | XAC1387 | hypothetical protein | -2.52 |
| *-* | XAC1388 | hypothetical protein | -2.00 |
| *-* | XAC1491 | hypothetical protein | -3.06 |
| *grpE* | XAC1521 | heat shock protein GrpE | -2.99 |
| *dnaK* | XAC1522 | molecular chaperone DnaK | -3.18 |
| *dnaJ* | XAC1523 | molecular chaperone DnaJ | -2.23 |
| *-* | XAC1563 | hypothetical protein | -3.12 |
| *-* | XAC1572 | hypothetical protein | -3.09 |
| *pstB* | XAC1574 | phosphate transporter ATP-binding protein | -2.18 |
| *pstA* | XAC1575 | ABC transporter phosphate permease | -2.32 |
| *pstC* | XAC1576 | ABC transporter phosphate permease | -2.38 |
| *-* | XAC1586 | MutT-nudix family protein | -2.04 |
| *-* | XAC1593 | ABC transporter ATP-binding protein | -2.10 |
| *-* | XAC1602 | hypothetical protein | -2.03 |
| *-* | XAC1605 | hypothetical protein | -3.25 |
| *-* | XAC1606 | hypothetical protein | -2.58 |
| *-* | XAC1607 | hypothetical protein | -2.50 |
| *hutG* | XAC1636 | formylglutamate amidohydrolase | -2.57 |
| *hutH* | XAC1637 | histidine ammonia-lyase | -2.31 |
| *hutI* | XAC1638 | imidazolonepropionase | -2.45 |
| *sdeB* | XAC1639 | N-formimino-L-glutamate deiminase | -2.81 |
| *-* | XAC1651 | TonB-like protein | -2.86 |
| *-* | XAC1658 | hypothetical protein | -2.64 |
| *-* | XAC1659 | hypothetical protein | -2.19 |
| *repA* | XAC1662 | hypothetical protein | -2.65 |
| *-* | XAC1663 | hypothetical protein | -2.93 |
| *-* | XAC1673 | hypothetical protein | -2.95 |
| *cycL* | XAC1674 | C-type cytochrome biogenesis protein | -2.58 |
| *cycK* | XAC1676 | C-type cytochrome biogenesis membrane protein | -2.93 |
| *-* | XAC1678 | hypothetical protein | -2.19 |
| *-* | XAC1685 | cytochrome C | -3.32 |
| *-* | XAC1686 | cytochrome like B561 | -3.17 |
| *-* | XAC1688 | hypothetical protein | -3.17 |
| *-* | XAC1689 | hypothetical protein | -2.89 |
| *-* | XAC1690 | hypothetical protein | -3.75 |
| *-* | XAC1691 | aminotransferase | -3.12 |
| *-* | XAC1692 | lipopolysaccharide biosynthesis protein | -3.37 |
| *-* | XAC1693 | glycosyl transferase | -3.32 |
| *-* | XAC1694 | hypothetical protein | -4.52 |
| *-* | XAC1695 | hypothetical protein | -2.74 |
| *-* | XAC1696 | methyltransferase | -3.17 |
| *-* | XAC1697 | hypothetical protein | -2.91 |
| *bioC* | XAC1698 | biotin synthesis protein | -2.58 |
| *-* | XAC1699 | glycosyltransferase | -2.64 |
| *-* | XAC1700 | hexosyltransferase | -3.17 |
| *-* | XAC1701 | hypothetical protein | -2.72 |
| *-* | XAC1702 | Mg-protoporphyrin IX monomethyl ester oxidative cyclase | -2.38 |
| *-* | XAC1703 | hypothetical protein | -2.70 |
| *-* | XAC1704 | ABC transporter ATP-binding protein | -2.94 |
| *-* | XAC1705 | MFS transporter | -2.42 |
| *-* | XAC1706 | hypothetical protein | -3.68 |
| *-* | XAC1748 | transcriptional regulator | -2.21 |
| *fhuA* | XAC1768 | TonB-dependent receptor | -2.00 |
| *celA* | XAC1770 | cellulase | -2.50 |
| *-* | XAC1778 | sensor kinase | -2.12 |
| *amiC* | XAC1780 | N-acetylmuramoyl-L-alanine amidase | -3.17 |
| *phoX* | XAC1792 | alkaline phosphatase | -2.54 |
| *regR* | XAC1797 | two-component system regulatory protein | -2.05 |
| *regS* | XAC1798 | two-component system sensor protein | -2.42 |
| *-* | XAC1810 | hypothetical protein | -2.32 |
| *hmsR* | XAC1811 | N-glycosyltransferase | -2.94 |
| *hmsF* | XAC1812 | HmsF protein | -3.58 |
| *hmsH* | XAC1813 | HmsH protein | -2.74 |
| *-* | XAC1816 | hemagglutinin/hemolysin-like protein | -2.32 |
| *-* | XAC1818 | hemagglutinin | -2.52 |
| *tspO* | XAC1819 | tryptophan-rich sensory protein | -2.95 |
| *thrB* | XAC1821 | homoserine kinase | -2.09 |
| *-* | XAC1827 | hypothetical protein | -2.58 |
| *hisG* | XAC1828 | ATP phosphoribosyltransferase | -2.77 |
| *hisD* | XAC1829 | histidinol dehydrogenase | -2.28 |
| *hisC* | XAC1830 | histidinol-phosphate aminotransferase | -2.55 |
| *hisB* | XAC1831 | imidazole glycerol-phosphate dehydratase/histidinol phosphatase | -2.66 |
| *hisH* | XAC1832 | imidazole glycerol phosphate synthase subunit HisH | -2.66 |
| *hisA* | XAC1833 | 1-(5-phosphoribosyl)-5-[(5-phosphoribosylamino)methylideneamino] imidazole-4-carboxamide isomerase | -2.28 |
| *hisF* | XAC1834 | imidazole glycerol phosphate synthase subunit HisF | -2.44 |
| *hisI* | XAC1835 | bifunctional phosphoribosyl-AMP cyclohydrolase/phosphoribosyl-ATP pyrophosphatase | -2.13 |
| *-* | XAC1873 | hypothetical protein | -2.43 |
| *-* | XAC1881 | hypothetical protein | -2.23 |
| *pcaD* | XAC1886 | beta-ketoadipate enol-lactone hydrolase | -4.75 |
| *-* | XAC1914 | hypothetical protein | -2.20 |
| *-* | XAC1916 | ISxac1 transposase | -2.08 |
| *-* | XAC1917 | hypothetical protein | -2.32 |
| *-* | XAC1918 | hemolysin-like protein | -2.65 |
| *-* | XAC1919 | hypothetical protein | -3.39 |
| *-* | XAC1924 | transposase | -2.81 |
| *-* | XAC1926 | hypothetical protein | -2.73 |
| *-* | XAC1928 | hypothetical protein | -2.77 |
| *-* | XAC1929 | ISxac1 transposase | -2.09 |
| *-* | XAC1943 | hypothetical protein | -2.81 |
| *-* | XAC1956 | hypothetical protein | -2.66 |
| *-* | XAC2009 | hypothetical protein | -2.26 |
| *cirA* | XAC2024 | TonB-dependent receptor | -2.77 |
| *-* | XAC2026 | hypothetical protein | -3.85 |
| *-* | XAC2027 | hypothetical protein | -3.91 |
| *fdh* | XAC2028 | glutathione-dependent formaldehyde dehydrogenase | -3.54 |
| *czcB* | XAC2064 | cation efflux system protein | -2.03 |
| *acrD* | XAC2065 | transporter | -2.29 |
| *syrE1* | XAC2097 | ATP-dependent serine activating enzyme | -2.58 |
| *syrE2* | XAC2098 | ATP-dependent serine activating enzyme | -2.00 |
| *-* | XAC2113 | hypothetical protein | -2.32 |
| *-* | XAC2122 | dehydrogenase | -2.16 |
| *-* | XAC2123 | hypothetical protein | -2.77 |
| *-* | XAC2124 | hypothetical protein | -2.56 |
| *gtrB* | XAC2125 | glycosyl transferase-like protein | -2.37 |
| *-* | XAC2127 | hypothetical protein | -2.28 |
| *-* | XAC2128 | 2-keto-gluconate dehydrogenase | -2.54 |
| *fabG* | XAC2129 | 3-ketoacyl-ACP reductase | -2.43 |
| *-* | XAC2133 | oxidoreductase | -3.27 |
| *-* | XAC2134 | hypothetical protein | -2.50 |
| *-* | XAC2140 | D-Ala-D-Ala carboxypeptidase | -3.09 |
| *lytS* | XAC2142 | two-component system sensor protein | -2.17 |
| *czcB* | XAC2145 | cation efflux system protein | -2.66 |
| *-* | XAC2146 | hypothetical protein | -3.64 |
| *czcA* | XAC2147 | cation efflux system protein | -2.87 |
| *ttgC* | XAC2148 | outer membrane efflux protein | -2.32 |
| *yapH* | XAC2151 | YapH protein | -2.46 |
| *-* | XAC2152 | hypothetical protein | -2.33 |
| *-* | XAC2155 | hypothetical protein | -3.99 |
| *cysG* | XAC2157 | uroporphyrin-III C-methyltransferase | -3.07 |
| *-* | XAC2158 | histidine kinase-response regulator hybrid protein | -3.32 |
| *-* | XAC2160 | hypothetical protein | -2.36 |
| *tetV* | XAC2161 | MFS transporter | -2.93 |
| *-* | XAC2162 | hypothetical protein | -2.94 |
| *-* | XAC2163 | hypothetical protein | -2.89 |
| *-* | XAC2164 | hypothetical protein | -3.29 |
| *-* | XAC2165 | hydrolase | -3.30 |
| *-* | XAC2166 | transcriptional regulator | -2.81 |
| *-* | XAC2172 | NADH dehydrogenase | -2.32 |
| *-* | XAC2178 | hypothetical protein | -2.42 |
| *cirA* | XAC2193 | TonB-dependent receptor | -2.00 |
| *-* | XAC2196 | hypothetical protein | -2.10 |
| *-* | XAC2197 | hemolysin-type calcium-binding protein | -2.55 |
| *-* | XAC2198 | hemolysin-type calcium-binding protein | -2.58 |
| *-* | XAC2199 | hypothetical protein | -2.86 |
| *-* | XAC2200 | hypothetical protein | -2.78 |
| *hlyD* | XAC2201 | hemolysin secretion protein D | -2.00 |
| *hlyB* | XAC2202 | hemolysin secretion protein B | -2.40 |
| *-* | XAC2203 | hypothetical protein | -3.17 |
| *-* | XAC2204 | hypothetical protein | -2.39 |
| *soj* | XAC2205 | chromosome partitioning-like protein | -3.50 |
| *-* | XAC2206 | hypothetical protein | -2.91 |
| *-* | XAC2207 | hypothetical protein | -3.17 |
| *-* | XAC2208 | hypothetical protein | -3.04 |
| *-* | XAC2209 | hypothetical protein | -3.17 |
| *-* | XAC2210 | hypothetical protein | -2.81 |
| *ssb* | XAC2211 | single-stranded DNA-binding protein | -2.27 |
| *topB* | XAC2212 | DNA topoisomerase III | -2.39 |
| *-* | XAC2213 | cytosine-specific DNA methyltransferase | -2.32 |
| *-* | XAC2217 | hypothetical protein | -2.58 |
| *-* | XAC2219 | hypothetical protein | -3.58 |
| *-* | XAC2220 | hypothetical protein | -3.58 |
| *-* | XAC2221 | hypothetical protein | -2.70 |
| *cynX* | XAC2234 | MFS transporter | -2.12 |
| *orf8* | XAC2243 | plasmid-like protein | -2.81 |
| *-* | XAC2248 | hypothetical protein | -3.17 |
| *-* | XAC2249 | hypothetical protein | -2.68 |
| *pilL* | XAC2253 | PilL protein | -3.00 |
| *-* | XAC2254 | hypothetical protein | -2.93 |
| *-* | XAC2255 | hypothetical protein | -2.58 |
| *-* | XAC2256 | hypothetical protein | -2.94 |
| *-* | XAC2257 | hypothetical protein | -2.81 |
| *-* | XAC2258 | hypothetical protein | -2.36 |
| *-* | XAC2259 | hypothetical protein | -2.87 |
| *-* | XAC2260 | hypothetical protein | -2.68 |
| *-* | XAC2268 | hypothetical protein | -2.66 |
| *-* | XAC2269 | hypothetical protein | -2.77 |
| *-* | XAC2270 | hypothetical protein | -2.70 |
| *-* | XAC2271 | hypothetical protein | -2.65 |
| *-* | XAC2272 | hypothetical protein | -2.62 |
| *-* | XAC2273 | hypothetical protein | -2.40 |
| *-* | XAC2274 | hypothetical protein | -2.32 |
| *nocR* | XAC2276 | transcriptional regulator | -2.32 |
| *-* | XAC2277 | 3-hydroxyisobutyrate dehydrogenase | -2.77 |
| *-* | XAC2279 | hypothetical protein | -2.68 |
| *-* | XAC2280 | hypothetical protein | -3.00 |
| *-* | XAC2282 | hypothetical protein | -2.66 |
| *-* | XAC2283 | hypothetical protein | -2.14 |
| *-* | XAC2284 | hypothetical protein | -2.72 |
| *orf84* | XAC2285 | hypothetical protein | -2.39 |
| *-* | XAC2357 | hypothetical protein | -2.56 |
| *-* | XAC2367 | hypothetical protein | -3.71 |
| *-* | XAC2368 | hypothetical protein | -3.88 |
| *-* | XAC2369 | general stress protein | -3.38 |
| *-* | XAC2371 | IS1479 transposase | -3.25 |
| *-* | XAC2372 | IS1479 transposase | -2.07 |
| *pel* | XAC2373 | degenerated pectate lyase | -2.66 |
| *htpX* | XAC2399 | heat shock protein HtpX | -4.29 |
| *lig3* | XAC2414 | ATP-dependent DNA ligase | -3.54 |
| *-* | XAC2415 | hypothetical protein | -3.50 |
| *-* | XAC2425 | hypothetical protein | -2.17 |
| *repA* | XAC2441 | replication protein | -2.37 |
| *lhr1* | XAC2450 | ATP-dependent DNA helicase | -2.32 |
| *gstA* | XAC2460 | glutathione S-transferase | -2.47 |
| *bioA* | XAC2477 | aminotransferase | -2.01 |
| *-* | XAC2517 | hypothetical protein | -4.09 |
| *-* | XAC2519 | hypothetical protein | -2.15 |
| *cirA* | XAC2520 | TonB-dependent receptor | -2.75 |
| *htpG* | XAC2528 | heat shock protein 90 | -4.37 |
| *rhsD* | XAC2529 | RhsD protein | -2.22 |
| *-* | XAC2530 | hypothetical protein | -2.25 |
| *btuB* | XAC2531 | TonB-dependent receptor | -2.91 |
| *xsa* | XAC2533 | arabinosidase | -2.26 |
| *-* | XAC2534 | hypothetical protein | -2.75 |
| *btuB* | XAC2535 | TonB-dependent receptor | -2.87 |
| *-* | XAC2536 | hypothetical protein | -2.66 |
| *-* | XAC2541 | peptidase | -2.15 |
| *yveA* | XAC2542 | amino acid permease | -2.28 |
| *pepQ* | XAC2545 | proline dipeptidase | -2.00 |
| *-* | XAC2548 | oxidoreductase | -2.58 |
| *-* | XAC2549 | D-amino acid oxidase | -2.38 |
| *-* | XAC2550 | hypothetical protein | -2.58 |
| *-* | XAC2556 | hypothetical protein | -2.95 |
| *-* | XAC2557 | hypothetical protein | -2.61 |
| *-* | XAC2598 | hypothetical protein | -2.05 |
| *aglA* | XAC2599 | alpha-glucosidase | -2.35 |
| *btuB* | XAC2600 | TonB-dependent receptor | -2.30 |
| *-* | XAC2601 | hypothetical protein | -2.68 |
| *aglA* | XAC2602 | alpha-glucosidase | -2.48 |
| *-* | XAC2604 | ISxac4 transposase | -2.81 |
| *-* | XAC2629 | hypothetical protein | -2.81 |
| *-* | XAC2630 | hypothetical protein | -2.38 |
| *-* | XAC2631 | hypothetical protein | -2.09 |
| *-* | XAC2632 | hypothetical protein | -2.94 |
| *-* | XAC2637 | hypothetical protein | -3.04 |
| *-* | XAC2638 | hypothetical protein | -2.54 |
| *-* | XAC2639 | site-specific DNA-methyltransferase | -3.39 |
| *-* | XAC2640 | hypothetical protein | -2.81 |
| *Q* | XAC2641 | phage-related capsid packaging protein | -2.81 |
| *P* | XAC2642 | phage-related terminase | -3.32 |
| *O* | XAC2643 | phage-related capsid scaffold protein | -3.91 |
| *N* | XAC2644 | phage-related major capsid protein | -2.58 |
| *M* | XAC2645 | phage-related terminase | -3.46 |
| *L* | XAC2646 | phage-related capsid completion protein | -3.00 |
| *X* | XAC2647 | phage-related tail protein | -3.46 |
| *orf89* | XAC2648 | hypothetical protein | -4.17 |
| *orf90* | XAC2649 | hypothetical protein | -3.09 |
| *lys* | XAC2650 | phage-related lytic protein | -2.94 |
| *-* | XAC2651 | hypothetical protein | -3.17 |
| *R* | XAC2652 | phage-related tail protein | -3.32 |
| *S* | XAC2653 | phage-related tail protein | -3.06 |
| *I* | XAC2656 | phage-related tail protein | -2.58 |
| *-* | XAC2733 | hypothetical protein | -2.62 |
| *-* | XAC2746 | metallopeptidase | -2.32 |
| *tldD* | XAC2767 | TldD protein | -2.25 |
| *bp26* | XAC2772 | hypothetical protein | -2.14 |
| *-* | XAC2776 | hypothetical protein | -2.32 |
| *trx* | XAC2783 | thioredoxin | -3.32 |
| *-* | XAC2786 | hypothetical protein | -2.86 |
| *-* | XAC2787 | hypothetical protein | -3.06 |
| *-* | XAC2788 | hypothetical protein | -2.78 |
| *-* | XAC2791 | transcriptional regulator | -2.03 |
| *-* | XAC2792 | hypothetical protein | -2.32 |
| *acr* | XAC2799 | acriflavin resistance protein | -2.54 |
| *mexC* | XAC2800 | RND efflux membrane fusion protein | -2.55 |
| *ttgF* | XAC2802 | outer membrane channel protein | -2.38 |
| *-* | XAC2821 | hypothetical protein | -2.13 |
| *-* | XAC2833 | serine protease | -2.17 |
| *mocA* | XAC2835 | oxidoreductase | -2.28 |
| *-* | XAC2838 | LysR family transcriptional regulator | -2.46 |
| *-* | XAC2841 | transcriptional regulator | -2.25 |
| *mexB* | XAC2843 | multidrug efflux transporter | -2.77 |
| *mexA* | XAC2844 | multidrug resistance protein | -2.27 |
| *nthA* | XAC2849 | nitrilase | -2.17 |
| *-* | XAC2851 | hypothetical protein | -2.49 |
| *creD* | XAC2852 | hypothetical protein | -2.49 |
| *-* | XAC2853 | cysteine protease | -3.24 |
| *-* | XAC2859 | hypothetical protein | -3.17 |
| *-* | XAC2886 | hypothetical protein | -2.06 |
| *-* | XAC2891 | hypothetical protein | -2.05 |
| *yagR* | XAC2893 | oxidoreductase | -2.35 |
| *yagS* | XAC2894 | oxidoreductase | -2.37 |
| *-* | XAC2920 | hypothetical protein | -2.63 |
| *pfpI* | XAC2932 | protease | -4.55 |
| *qxtB* | XAC2982 | quinol oxidase subunit II | -2.81 |
| *-* | XAC2983 | quinol oxidase subunit I | -3.22 |
| *-* | XAC2987 | proline imino-peptidase | -2.94 |
| *-* | XAC2989 | amino acid transporter | -2.17 |
| *-* | XAC2990 | hypothetical protein | -2.66 |
| *-* | XAC2991 | hypothetical protein | -3.09 |
| *prnA* | XAC2995 | tryptophan halogenase | -2.00 |
| *fecA* | XAC2998 | TonB-dependent receptor | -2.74 |
| *ptr* | XAC3001 | MFS transporter | -2.26 |
| *-* | XAC3025 | hypothetical protein | -2.22 |
| *-* | XAC3026 | transcriptional regulator | -2.96 |
| *-* | XAC3030 | hypothetical protein | -3.64 |
| *-* | XAC3031 | histidine kinase-response regulator hybrid protein | -2.43 |
| *-* | XAC3038 | homoserine dehydrogenase | -2.69 |
| *metB* | XAC3039 | cystathionine gamma-synthase | -2.50 |
| *metA* | XAC3040 | homoserine O-acetyltransferase | -2.03 |
| *-* | XAC3048 | heat shock protein | -2.96 |
| *-* | XAC3053 | hypothetical protein | -2.63 |
| *fadE* | XAC3054 | acyl-CoA dehydrogenase | -2.17 |
| *-* | XAC3062 | hypothetical protein | -2.58 |
| *-* | XAC3063 | hypothetical protein | -2.39 |
| *iroN* | XAC3071 | TonB-dependent receptor | -2.38 |
| *fucA1* | XAC3072 | alpha-L-fucosidase | -2.46 |
| *cirA* | XAC3077 | TonB-dependent receptor | -3.17 |
| *bga* | XAC3078 | beta-galactosidase | -2.75 |
| *yhfM* | XAC3079 | cationic amino acid transporter | -3.25 |
| *rbsK* | XAC3080 | ribokinase | -2.68 |
| *celF* | XAC3081 | 6-phospho-beta-glucosidase | -2.54 |
| *-* | XAC3085 | hypothetical protein | -2.26 |
| *-* | XAC3093 | hypothetical protein | -2.75 |
| *pqqG* | XAC3114 | pyrroloquinoline quinone biosynthesis protein PqqB | -4.26 |
| *pqqC* | XAC3115 | pyrroloquinoline quinone biosynthesis protein PqqC | -4.19 |
| *pqqC/D* | XAC3116 | PqqC/D protein | -4.29 |
| *pqqE* | XAC3117 | pyrroloquinoline quinone biosynthesis protein PqqE | -3.79 |
| *-* | XAC3131 | hypothetical protein | -2.81 |
| *fhuA* | XAC3158 | TonB-dependent receptor | -2.50 |
| *-* | XAC3161 | hypothetical protein | -2.17 |
| *bla* | XAC3162 | beta lactamase | -2.24 |
| *bioI* | XAC3170 | cytochrome P-450 hydroxylase | -2.64 |
| *clpB* | XAC3195 | ATP-dependent Clp protease subunit | -4.03 |
| *-* | XAC3203 | glutathione transferase | -2.39 |
| *-* | XAC3204 | hypothetical protein | -3.25 |
| *orfS* | XAC3227 | cointegrate resolution protein S | -2.66 |
| *-* | XAC3233 | transposase | -2.70 |
| *glgX* | XAC3254 | glycogen debranching protein | -2.95 |
| *-* | XAC3261 | hypothetical protein | -3.22 |
| *-* | XAC3265 | hypothetical protein | -2.32 |
| *-* | XAC3269 | RadC family protein | -4.00 |
| *-* | XAC3275 | hypothetical protein | -2.54 |
| *-* | XAC3276 | hypothetical protein | -2.58 |
| *-* | XAC3282 | integrase | -3.91 |
| *-* | XAC3285 | hypothetical protein | -2.91 |
| *-* | XAC3291 | hypothetical protein | -2.56 |
| *-* | XAC3295 | hypothetical protein | -2.49 |
| *iroN* | XAC3311 | TonB-dependent receptor | -2.52 |
| *-* | XAC3312 | glycosyl hydrolase | -2.05 |
| *susB* | XAC3313 | alpha-glucosidase | -2.81 |
| *-* | XAC3333 | hypothetical protein | -2.72 |
| *fecA* | XAC3334 | TonB-dependent receptor | -2.22 |
| *-* | XAC3353 | hypothetical protein | -2.42 |
| *-* | XAC3374 | hypothetical protein | -3.42 |
| *-* | XAC3375 | hypothetical protein | -3.55 |
| *-* | XAC3376 | hypothetical protein | -3.56 |
| *-* | XAC3377 | hypothetical protein | -3.50 |
| *-* | XAC3378 | hypothetical protein | -3.49 |
| *moxR* | XAC3379 | methanol dehydrogenase regulatory protein | -3.51 |
| *btuB* | XAC3448 | TonB-dependent receptor | -2.09 |
| *dctA* | XAC3471 | C4-dicarboxylate transporter DctA | -2.58 |
| *cit1* | XAC3474 | citrate carrier protein | -2.36 |
| *-* | XAC3475 | hypothetical protein | -2.32 |
| *mocA* | XAC3477 | rhizopine catabolism protein MocA | -2.32 |
| *-* | XAC3478 | hypothetical protein | -2.81 |
| *-* | XAC3479 | hypothetical protein | -2.28 |
| *oprO* | XAC3484 | porin | -3.00 |
| *citM* | XAC3485 | Mg++/citrate complex transporter | -3.13 |
| *fabG* | XAC3486 | 3-ketoacyl-ACP reductase | -2.58 |
| *suc1* | XAC3488 | sugar transporter | -2.36 |
| *fyuA* | XAC3489 | TonB-dependent receptor | -2.20 |
| *-* | XAC3490 | amylosucrase or alpha amylase | -2.26 |
| *-* | XAC3497 | hypothetical protein | -3.32 |
| *-* | XAC3504 | ISxac4 transposase | -3.00 |
| *bcsC* | XAC3515 | cellulose synthase subunit C | -2.04 |
| *-* | XAC3516 | endo-1,4-D-glucanase | -2.37 |
| *-* | XAC3517 | cellulose synthase regulator protein | -2.32 |
| *bcsA* | XAC3518 | celullose synthase | -2.77 |
| *-* | XAC3527 | hypothetical protein | -2.68 |
| *xpsI* | XAC3540 | general secretion pathway protein I | -2.00 |
| *-* | XAC3547 | serine protease | -2.91 |
| *pel* | XAC3562 | pectate lyase | -2.64 |
| *ybjY* | XAC3638 | ABC transporter permease | -2.35 |
| *ybjZ* | XAC3640 | ABC transporter ATP-binding protein | -2.04 |
| *-* | XAC3680 | hypothetical protein | -4.49 |
| *-* | XAC3684 | hypothetical protein | -2.14 |
| *-* | XAC3685 | hypothetical protein | -2.21 |
| *-* | XAC3686 | hypothetical protein | -3.97 |
| *-* | XAC3690 | hypothetical protein | -4.44 |
| *-* | XAC3691 | methionine sulfoxide reductase B | -4.46 |
| *-* | XAC3692 | hypothetical protein | -4.15 |
| *-* | XAC3702 | hypothetical protein | -2.97 |
| *-* | XAC3703 | hypothetical protein | -2.65 |
| *-* | XAC3707 | hypothetical protein | -2.27 |
| *-* | XAC3711 | hypothetical protein | -2.30 |
| *-* | XAC3712 | metallopeptidase | -2.13 |
| *-* | XAC3713 | peptidase | -2.05 |
| *-* | XAC3716 | hypothetical protein | -2.32 |
| *-* | XAC3720 | hypothetical protein | -2.00 |
| *-* | XAC3722 | hypothetical protein | -2.63 |
| *-* | XAC3724 | hypothetical protein | -2.17 |
| *-* | XAC3725 | hypothetical protein | -4.66 |
| *-* | XAC3726 | hypothetical protein | -5.76 |
| *-* | XAC3727 | hypothetical protein | -4.55 |
| *-* | XAC3729 | hypothetical protein | -2.74 |
| *exsF* | XAC3731 | regulatory protein | -2.71 |
| *-* | XAC3734 | hypothetical protein | -2.96 |
| *cioA* | XAC3735 | cyanide insensitive terminal oxidase | -2.67 |
| *-* | XAC3737 | hypothetical protein | -3.99 |
| *-* | XAC3738 | oxidoreductase | -5.05 |
| *-* | XAC3739 | hypothetical protein | -2.32 |
| *-* | XAC3740 | UDP-glucose 4-epimerase | -3.20 |
| *-* | XAC3741 | hypothetical protein | -2.91 |
| *rfbD* | XAC3742 | UDP-galactopyranose mutase | -2.04 |
| *-* | XAC3743 | hypothetical protein | -2.00 |
| *msbA* | XAC3744 | ATP-binding transporter 1 | -2.55 |
| *-* | XAC3745 | hypothetical protein | -6.07 |
| *-* | XAC3746 | hypothetical protein | -5.87 |
| *ybdR* | XAC3747 | Zn-dependent alcohol dehydrogenase | -5.46 |
| *-* | XAC3748 | hypothetical protein | -2.76 |
| *-* | XAC3749 | hypothetical protein | -2.97 |
| *-* | XAC3750 | hypothetical protein | -2.64 |
| *-* | XAC3756 | ferrichrome-iron receptor 3 | -2.04 |
| *-* | XAC3757 | hypothetical protein | -2.84 |
| *-* | XAC3758 | hypothetical protein | -2.72 |
| *-* | XAC3760 | hypothetical protein | -2.19 |
| *-* | XAC3778 | hypothetical protein | -2.22 |
| *-* | XAC3779 | hypothetical protein | -2.87 |
| *-* | XAC3780 | chloride channel | -2.38 |
| *-* | XAC3844 | hypothetical protein | -5.08 |
| *-* | XAC3845 | hypothetical protein | -4.48 |
| *-* | XAC3856 | hypothetical protein | -4.60 |
| *-* | XAC3865 | hypothetical protein | -3.94 |
| *-* | XAC3866 | hypothetical protein | -3.49 |
| *-* | XAC3892 | SSU 5S ribosomal RNA | -3.70 |
| *-* | XAC3938 | ISxac3 transposase | -2.81 |
| *-* | XAC3942 | hypothetical protein | -2.44 |
| *-* | XAC3966 | hypothetical protein | -3.77 |
| *-* | XAC3967 | hydrolase | -2.58 |
| *-* | XAC3968 | hypothetical protein | -2.91 |
| *-* | XAC3969 | hypothetical protein | -2.73 |
| *-* | XAC3976 | hypothetical protein | -2.13 |
| *-* | XAC3977 | hypothetical protein | -3.00 |
| *-* | XAC3984 | hypothetical protein | -3.32 |
| *prtI* | XAC3989 | ECF sigma factor | -2.02 |
| *-* | XAC3999 | hypothetical protein | -2.25 |
| *trpS* | XAC4006 | tryptophanyl-tRNA synthetase | -2.33 |
| *-* | XAC4007 | hypothetical protein | -4.89 |
| *-* | XAC4020 | hypothetical protein | -2.82 |
| *-* | XAC4021 | hypothetical protein | -3.24 |
| *-* | XAC4027 | hypothetical protein | -2.90 |
| *ankB* | XAC4028 | ankyrin-like protein | -2.70 |
| *-* | XAC4037 | endonuclease | -2.58 |
| *-* | XAC4039 | hypothetical protein | -2.61 |
| *-* | XAC4043 | hypothetical protein | -2.27 |
| *-* | XAC4061 | hypothetical protein | -2.44 |
| *fhuA* | XAC4062 | TonB-dependent receptor | -2.10 |
| *-* | XAC4063 | hypothetical protein | -2.46 |
| *ftrA* | XAC4064 | transcriptional activator FtrA | -2.22 |
| *-* | XAC4108 | hypothetical protein | -2.20 |
| *-* | XAC4112 | hypothetical protein | -2.30 |
| *yapH* | XAC4113 | YapH protein | -2.36 |
| *shlB* | XAC4114 | hemolysin activator protein | -2.29 |
| *-* | XAC4115 | hypothetical protein | -2.36 |
| *-* | XAC4116 | serine/threonine kinase | -2.50 |
| *ptc1* | XAC4117 | phosphoprotein phosphatase | -2.51 |
| *-* | XAC4118 | hypothetical protein | -2.56 |
| *-* | XAC4119 | hypothetical protein | -2.74 |
| *-* | XAC4120 | hypothetical protein | -3.03 |
| *-* | XAC4121 | hypothetical protein | -2.52 |
| *-* | XAC4122 | hypothetical protein | -2.44 |
| *-* | XAC4123 | hypothetical protein | -2.70 |
| *-* | XAC4124 | hypothetical protein | -2.17 |
| *-* | XAC4125 | hypothetical protein | -2.51 |
| *-* | XAC4126 | hypothetical protein | -2.56 |
| *rpoE* | XAC4129 | ECF sigma factor | -2.68 |
| *-* | XAC4130 | transmembrane sensor | -2.52 |
| *-* | XAC4131 | hypothetical protein | -2.58 |
| *appA* | XAC4132 | 6-phytase | -2.51 |
| *-* | XAC4133 | hypothetical protein | -2.72 |
| *-* | XAC4134 | hypothetical protein | -2.95 |
| *-* | XAC4137 | ISxac1 transposase | -2.05 |
| *-* | XAC4138 | transposase | -2.52 |
| *-* | XAC4139 | hypothetical protein | -2.44 |
| *clpB* | XAC4140 | chaperone ClpB | -2.89 |
| *-* | XAC4141 | hypothetical protein | -2.46 |
| *-* | XAC4142 | hypothetical protein | -2.74 |
| *-* | XAC4143 | hypothetical protein | -3.32 |
| *-* | XAC4144 | hypothetical protein | -2.54 |
| *-* | XAC4145 | hypothetical protein | -2.46 |
| *-* | XAC4146 | hypothetical protein | -2.43 |
| *-* | XAC4147 | hypothetical protein | -2.20 |
| *feaR* | XAC4148 | transcriptional regulator | -3.06 |
| *fldZ* | XAC4155 | hypothetical protein | -2.46 |
| *fldA* | XAC4156 | FldA protein | -2.47 |
| *fldW* | XAC4157 | 4-oxalomesaconate hydratase | -2.42 |
| *czcA* | XAC4160 | cation efflux system protein | -2.40 |
| *czcB* | XAC4161 | cation efflux system protein | -2.74 |
| *czcC* | XAC4162 | cation efflux system protein | -2.23 |
| *phoD* | XAC4166 | alkaline phosphatase | -2.44 |
| *-* | XAC4167 | hypothetical protein | -2.21 |
| *-* | XAC4172 | transcriptional regulator | -2.05 |
| *-* | XAC4173 | hypothetical protein | -2.44 |
| *-* | XAC4174 | peptidyl-prolyl cis-trans isomerase | -2.43 |
| *-* | XAC4175 | hypothetical protein | -2.58 |
| *-* | XAC4184 | oxidoreductase | -2.02 |
| *-* | XAC4185 | hypothetical protein | -2.30 |
| *-* | XAC4188 | RTS beta protein | -2.26 |
| *-* | XAC4189 | hypothetical protein | -2.56 |
| *fucP* | XAC4190 | fucose permease | -2.62 |
| *-* | XAC4194 | hypothetical protein | -2.32 |
| *-* | XAC4199 | polyvinylalcohol dehydrogenase | -3.61 |
| *-* | XAC4205 | hypothetical protein | -2.97 |
| *-* | XAC4206 | hypothetical protein | -3.32 |
| *aguA* | XAC4227 | alpha-glucuronidase | -2.54 |
| *-* | XAC4228 | sialic acid-specific 9-O-acetylesterase | -2.47 |
| *xylB* | XAC4230 | arabinosidase | -2.24 |
| *-* | XAC4231 | glucan 1,4-beta-glucosidase | -2.39 |
| *-* | XAC4235 | hypothetical protein | -2.87 |
| *alkH* | XAC4238 | aldehyde dehydrogenase | -2.16 |
| *-* | XAC4239 | transmembrane protein | -2.62 |
| *-* | XAC4240 | hypothetical protein | -2.58 |
| *hemL* | XAC4241 | glutamate-1-semialdehyde aminotransferase | -2.43 |
| *hemL* | XAC4242 | glutamate-1-semialdehyde 2,1-aminomutase | -2.38 |
| *fucA* | XAC4243 | L-fuculose phosphate aldolase | -2.24 |
| *xylB* | XAC4244 | xylulose kinase | -2.32 |
| *-* | XAC4245 | hypothetical protein | -2.93 |
| *-* | XAC4246 | hypothetical protein | -2.68 |
| *-* | XAC4247 | hypothetical protein | -2.91 |
| *gnl* | XAC4248 | gluconolactonase | -2.68 |
| *xynA* | XAC4249 | endo-1,4-beta-xylanase A | -2.21 |
| *-* | XAC4250 | beta-galactosidase | -2.49 |
| *hrmI* | XAC4251 | glucuronate isomerase | -2.37 |
| *xynB* | XAC4252 | xylanase | -2.58 |
| *xynB* | XAC4254 | xylanase | -2.46 |
| *exuT* | XAC4255 | hexuranate transporter | -3.04 |
| *cirA* | XAC4256 | TonB-dependent receptor | -3.25 |
| *xylP* | XAC4257 | transporter | -2.58 |
| *xsa* | XAC4258 | arabinosidase | -2.70 |
| *-* | XAC4287 | SSU 5S ribosomal RNA | -3.58 |
| *-* | XAC4309 | phosphotransferase | -2.32 |
| *-* | XAC4319 | hypothetical protein | -2.42 |
| *-* | XAC4321 | hypothetical protein | -2.63 |
| *-* | XAC4324 | hypothetical protein | -2.70 |
| *-* | XAC4325 | ISxac1 transposase | -2.06 |
| *-* | XAC4328 | ISxac1 transposase | -2.07 |
| *-* | XAC4329 | hypothetical protein | -2.81 |
| *-* | XAC4333 | hypothetical protein | -2.09 |
| *-* | XAC4334 | hypothetical protein | -2.70 |
| *-* | XAC4359 | sugar diacide regulator | -2.37 |
| *glxK* | XAC4360 | glycerate kinase | -2.00 |
| *-* | XAC4366 | hypothetical protein | -2.16 |
| *fecA* | XAC4368 | TonB-dependent receptor | -2.58 |
| *phoC* | XAC4369 | phosphatase | -2.26 |
| *-* | XACa0012 | hypothetical protein | -2.15 |
| *-* | XACa0031 | transposase | -3.14 |
| *-* | XACa0033 | ISxac1 transposase | -2.06 |
| *fpvA* | XAC0176 | ferripyoverdine receptor | 2.93 |
| *-* | XAC0190 | hypothetical protein | 2.08 |
| *fabH* | XAC0233 | 3-oxoacyl-ACP synthase | 3.11 |
| *-* | XAC0260 | hypothetical protein | 3.441 |
| *-* | XAC0271 | hypothetical protein | 2.43 |
| *-* | XAC0272 | hypothetical protein | 3.37 |
| *oar* | XAC0291 | Oar protein | 2.06 |
| *-* | XAC0350 | hypothetical protein | 2.11 |
| *hemH* | XAC0470 | phosphoribosylaminoimidazole-succinocarboxamide synthase | 2.40 |
| *-* | XAC0490 | Met tRNA | 2.29 |
| *-* | XAC0492 | bacterioferritin-associated ferredoxin | 2.77 |
| *-* | XAC0506 | transmembrane protein | 2.678938649 |
| *fis* | XAC0522 | Fis family transcriptional regulator | 2.068641666 |
| *-* | XAC0655 | sugar kinase | 2.054207074 |
| *-* | XAC0747 | hypothetical protein | 3.0138058 |
| *-* | XAC0822 | hypothetical protein | 5.936031112 |
| *phuR* | XAC0823 | outer membrane hemin receptor | 4.311944006 |
| *-* | XAC0824 | hypothetical protein | 2.160464672 |
| *-* | XAC0825 | hypothetical protein | 2.761160099 |
| *nrtCD* | XAC0828 | ABC transporter ATP-binding protein | 2.185111405 |
| *-* | XAC0829 | ABC transporter substrate-binding protein | 3.459431619 |
| *tauD* | XAC0830 | taurine dioxygenase | 3.584962501 |
| *-* | XAC0949 | Gln tRNA | 2.327413634 |
| *prsA* | XAC0950 | ribose-phosphate pyrophosphokinase | 2.363216303 |
| *rplY* | XAC0951 | 50S ribosomal protein L25 | 2.107412942 |
| *-* | XAC0954 | Tyr tRNA | 2.740031897 |
| *-* | XAC0955 | Gly tRNA | 2.411299314 |
| *-* | XAC0956 | Thr tRNA | 2.171370588 |
| *tuf* | XAC0957 | elongation factor Tu | 2.325790735 |
| *-* | XAC0958 | Trp tRNA | 2.857980995 |
| *nusG* | XAC0960 | transcription antitermination protein NusG | 2.400122825 |
| *rpsH* | XAC0986 | 30S ribosomal protein S8 | 2.081703572 |
| *rplF* | XAC0987 | 50S ribosomal protein L6 | 2.123016259 |
| *rplR* | XAC0988 | 50S ribosomal protein L18 | 2.173505526 |
| *secY* | XAC0992 | preprotein translocase subunit SecY | 2.081724397 |
| *typA* | XAC1004 | GTP-binding elongation factor protein | 2.182415946 |
| *-* | XAC1008 | hypothetical protein | 5.596338864 |
| *-* | XAC1048 | Pro tRNA | 2.435675229 |
| *-* | XAC1049 | Arg tRNA | 2.572794205 |
| *-* | XAC1050 | His tRNA | 2.185866545 |
| *-* | XAC1073 | Lys tRNA | 2.256339753 |
| *-* | XAC1082 | Val tRNA | 2.042821283 |
| *-* | XAC1083 | Asp tRNA | 2.519420245 |
| *-* | XAC1092 | Ser tRNA | 2.723376529 |
| *fabD* | XAC1126 | ACP S-malonyltransferase | 2.975033084 |
| *fabG* | XAC1127 | 3-ketoacyl-ACP reductase | 3.34811995 |
| *fyuA* | XAC1143 | TonB-dependent receptor | 3.084256851 |
| *rpmA* | XAC1249 | 50S ribosomal protein L27 | 2.112067775 |
| *rpsT* | XAC1251 | 30S ribosomal protein S20 | 2.594726 |
| *ileS* | XAC1254 | isoleucyl-tRNA synthetase | 2.047305715 |
| *-* | XAC1264 | hypothetical protein | 3.357117523 |
| *-* | XAC1282 | two-component system sensor protein | 3.416757246 |
| *-* | XAC1284 | two-component system regulatory protein | 3.701496251 |
| *rpsP* | XAC1292 | 30S ribosomal protein S16 | 2.689762658 |
| *rplS* | XAC1295 | 50S ribosomal protein L19 | 2.627229552 |
| *scoF* | XAC1337 | cold shock protein | 2.447981598 |
| *pyrH* | XAC1419 | uridylate kinase | 2.101283336 |
| *tsf* | XAC1421 | elongation factor Ts | 2.130531563 |
| *fhuA* | XAC1435 | iron permease | 3.815467568 |
| *-* | XAC1452 | hypothetical protein | 2.838972342 |
| *fpr* | XAC1458 | ferredoxin-NADP reductase | 2.428186782 |
| *-* | XAC1502 | hypothetical protein | 2.028014376 |
| *rpsF* | XAC1620 | 30S ribosomal protein S6 | 2.482717728 |
| *rpsR* | XAC1621 | 30S ribosomal protein S18 | 2.393342428 |
| *rplI* | XAC1622 | 50S ribosomal protein L9 | 2.989871044 |
| *cheR* | XAC1890 | chemotaxis protein methyltransferase | 2.674088391 |
| *tsr* | XAC1896 | chemotaxis protein | 2.600392541 |
| *tsr* | XAC1900 | chemotaxis protein | 2.256339753 |
| *-* | XAC1901 | hypothetical protein | 3.500802053 |
| *cheA* | XAC1903 | chemotaxis protein | 2.310150927 |
| *cheY* | XAC1904 | chemotaxis response regulator | 2.750512126 |
| *-* | XAC1905 | hypothetical protein | 2.866248611 |
| *parA* | XAC1907 | chromosome partioning protein | 2.408805546 |
| *motB* | XAC1908 | flagellar motor protein MotD | 2.212993723 |
| *fleN* | XAC1934 | flagellar biosynthesis switch protein | 2.347350692 |
| *-* | XAC1972 | hypothetical protein | 2.209881783 |
| *fliS* | XAC1973 | flagellar protein | 2.589669503 |
| *fliD* | XAC1974 | flagellar protein | 2.982993575 |
| *fliC* | XAC1975 | flagellin | 3.797768873 |
| *cheV* | XAC1987 | chemotaxis protein | 2.365181293 |
| *flgM* | XAC1989 | flagellar protein | 2.804732634 |
| *-* | XAC1990 | hypothetical protein | 2.222392421 |
| *-* | XAC1993 | hypothetical protein | 2.305345211 |
| *mcp* | XAC1996 | chemotaxis protein | 3.074835233 |
| *infA* | XAC2002 | translation initiation factor IF-1 | 2.332547349 |
| *orn* | XAC2039 | oligoribonuclease | 3.021061616 |
| *-* | XAC2094 | Gly tRNA | 2.921191159 |
| *-* | XAC2095 | Cys tRNA | 2.95090545 |
| *-* | XAC2096 | Gly tRNA | 2.64385619 |
| *-* | XAC2156 | hypothetical protein | 3.594041795 |
| *-* | XAC2169 | methyl-accepting chemotaxis protein | 3.417085328 |
| *ea31* | XAC2214 | hypothetical protein | 2.108252891 |
| *ea59* | XAC2215 | hypothetical protein | 2.68182404 |
| *rpmJ* | XAC2300 | 50S ribosomal protein L36 | 3.592515895 |
| *-* | XAC2301 | hypothetical protein | 2.411924887 |
| *-* | XAC2312 | hypothetical protein | 2.10780329 |
| *-* | XAC2398 | hypothetical protein | 3.054989235 |
| *rrpX* | XAC2482 | transcriptional regulator | 2.584962501 |
| *-* | XAC2490 | hypothetical protein | 2.251783271 |
| *-* | XAC2560 | Phe tRNA | 2.527737632 |
| *ihfA* | XAC2588 | integration host factor subunit alpha | 2.052851882 |
| *rplT* | XAC2591 | 50S ribosomal protein L20 | 2.151057469 |
| *rpmI* | XAC2592 | 50S ribosomal protein L35 | 2.193479729 |
| *-* | XAC2622 | hypothetical protein | 2.256339753 |
| *-* | XAC2663 | transposase | 2.096861539 |
| *pilE* | XAC2664 | PilE protein | 2.554030641 |
| *pilY1* | XAC2665 | PilY1 protein | 2.132450296 |
| *pilX* | XAC2666 | PilX protein | 2.424805276 |
| *-* | XAC2667 | hypothetical protein | 3.098302074 |
| *pilV* | XAC2668 | pre-pilin leader sequence | 3.487665299 |
| *fimT* | XAC2669 | pre-pilin like leader sequence | 3.428499759 |
| *oar* | XAC2672 | Oar protein | 3.714245518 |
| *-* | XAC2676 | hypothetical protein | 2.075874867 |
| *nusA* | XAC2688 | transcription elongation factor NusA | 2.024434218 |
| *-* | XAC2689 | hypothetical protein | 2.463400521 |
| *-* | XAC2690 | Met tRNA | 3.297904866 |
| *nuoN* | XAC2691 | NADH dehydrogenase subunit N | 2.051301229 |
| *nuoL* | XAC2693 | NADH dehydrogenase subunit L | 2.275839862 |
| *nuoK* | XAC2694 | NADH dehydrogenase subunit K | 2.1016767 |
| *nuoJ* | XAC2695 | NADH dehydrogenase subunit J | 2.106199404 |
| *nuoF* | XAC2699 | NADH-ubiquinone oxidoreductase NQO1 subunit | 2.163666011 |
| *nuoE* | XAC2700 | NADH dehydrogenase subunit E | 2.075767508 |
| *nuoD* | XAC2701 | NADH dehydrogenase subunit D | 2.198719592 |
| *nuoC* | XAC2702 | NADH dehydrogenase subunit C | 2.052984456 |
| *nuoB* | XAC2703 | NADH dehydrogenase subunit B | 2.380586223 |
| *nuoA* | XAC2704 | NADH dehydrogenase subunit A | 2.690757165 |
| *-* | XAC2705 | Leu tRNA | 2.712243165 |
| *secG* | XAC2706 | preprotein translocase subunit SecG | 2.045176139 |
| *slyD* | XAC2754 | peptidyl-prolyl cis-trans isomerase | 2.436805314 |
| *-* | XAC2764 | hypothetical protein | 2.337248894 |
| *-* | XAC2827 | hypothetical protein | 2.360043467 |
| *-* | XAC2864 | hypothetical protein | 2.440572591 |
| *pilU* | XAC2923 | twitching motility protein | 2.200139614 |
| *pilT* | XAC2924 | twitching motility protein | 2.014950341 |
| *fhuA* | XAC2941 | TonB-dependent receptor | 3.230297619 |
| *-* | XAC2942 | hydroxylase | 2.584962501 |
| *btuB* | XAC3050 | TonB-dependent receptor | 2.995661064 |
| *-* | XAC3086 | hypothetical protein | 2.329920886 |
| *rebB* | XAC3087 | RebB protein | 2.584962501 |
| *-* | XAC3096 | hypothetical protein | 2.109465979 |
| *pilJ* | XAC3099 | pilus biogenesis protein | 2.675309003 |
| *pilI* | XAC3100 | pilus biogenesis protein | 2.541146268 |
| *pilH* | XAC3101 | PilH protein | 2.594300365 |
| *-* | XAC3156 | hypothetical protein | 2.126108769 |
| *bfeA* | XAC3166 | ferric enterobactin receptor | 2.297680549 |
| *mphE* | XAC3175 | 4-hydroxy-2-oxovalerate aldolase | 3.372554168 |
| *fecA* | XAC3176 | citrate-dependent iron transporter | 4.50371218 |
| *-* | XAC3177 | hypothetical protein | 4.957195677 |
| *-* | XAC3178 | hypothetical protein | 3.375866902 |
| *yceE* | XAC3179 | transporter | 2.094517599 |
| *iucA* | XAC3180 | iron transporter | 2.938599455 |
| *lysA* | XAC3181 | diaminopimelate decarboxylase | 2.817135943 |
| *btuB* | XAC3194 | vitamin B transport outer membrane protein | 2.442004547 |
| *fimA* | XAC3241 | fimbrillin | 3.193678048 |
| *nodQ* | XAC3328 | bifunctional sulfate adenylyltransferase subunit 1/adenylylsulfate kinase | 2.193603998 |
| *cysD* | XAC3329 | sulfate adenylyltransferase subunit 2 | 2.462000978 |
| *cysJ* | XAC3330 | NADPH-sulfite reductase flavoprotein subunit | 3.439334049 |
| *cysI* | XAC3331 | sulfite reductase subunit beta | 3.948600847 |
| *cysH* | XAC3332 | phosphoadenosine phosphosulfate reductase | 4.591276275 |
| *cysG* | XAC3340 | siroheme synthase | 3.338558769 |
| *cysK* | XAC3341 | cysteine synthase | 2.927649156 |
| *-* | XAC3351 | hypothetical protein | 2.63992896 |
| *fhuE* | XAC3370 | ferric iron uptake outer membrane protein | 4.904652123 |
| *pilO* | XAC3383 | fimbrial assembly membrane protein | 2.14839184 |
| *pilM* | XAC3385 | fimbrial assembly membrane protein | 2.747612838 |
| *rpmE2* | XAC3389 | 50S ribosomal protein L31 | 2.82871192 |
| *rpoZ* | XAC3394 | DNA-directed RNA polymerase subunit omega | 2.825845339 |
| *oprO* | XAC3472 | polyphosphate-selective porin O | 2.486184346 |
| *fhuE* | XAC3498 | ferric iron uptake outer membrane protein | 2.222392421 |
| *-* | XAC3597 | hypothetical protein | 2.034488376 |
| *btuB* | XAC3613 | TonB-dependent receptor | 2.134649527 |
| *-* | XAC3619 | hypothetical protein | 2.147898695 |
| *pfeA* | XAC3620 | outer membrane receptor FepA | 3.208271313 |
| *-* | XAC3635 | hypothetical protein | 2.357552005 |
| *atpA* | XAC3651 | ATP synthase F0F1 subunit alpha | 2.121990524 |
| *atpE* | XAC3654 | ATP synthase F0F1 subunit C | 2.177586703 |
| *atpB* | XAC3655 | ATP synthase F0F1 subunit A | 2.089027756 |
| *-* | XAC3698 | hypothetical protein | 2.885195525 |
| *-* | XAC3783 | hypothetical protein | 2.097610797 |
| *-* | XAC3802 | hypothetical protein | 2.622990402 |
| *rpsU* | XAC3872 | 30S ribosomal protein S21 | 3.391596111 |
| *-* | XAC3873 | hypothetical protein | 2.892017319 |
| *-* | XAC3894 | Ile tRNA | 2.513852955 |
| *-* | XAC3895 | Ala tRNA | 2.567883042 |
| *dpm1* | XAC3909 | dolichol-phosphate mannosyltransferase | 2.376148486 |
| *-* | XAC3910 | hypothetical protein | 2.124695747 |
| *-* | XAC3963 | Ala tRNA | 7.615004242 |
| *-* | XAC3964 | hypothetical protein | 2.964501319 |
| *-* | XAC4010 | hypothetical protein | 2.046293652 |
| *-* | XAC4026 | hypothetical protein | 3.136372442 |
| *rpmG* | XAC4158 | 50S ribosomal protein L33 | 2.548335193 |
| *rpmB* | XAC4159 | 50S ribosomal protein L28 | 2.658827299 |
| *-* | XAC4289 | Ile tRNA | 2.513852955 |
| *-* | XAC4290 | Ala tRNA | 2.567883042 |
| *-* | XAC4357 | hypothetical protein | 2.125530882 |
| *rpmH* | XAC4374 | 50S ribosomal protein L34 | 2.000727715 |
