# Supplementary material for: Stringent response regulators (p)ppGpp and DksA positively regulate virulence and host adaptation of Xanthomonas citri
Source: Mol Plant Pathol. 2019 Oct 17;20(11):1550–65. doi: 10.1111/mpp.12865 (PMC6804348; doi:10.1111/mpp.12865)
Supplement: Supplementary file 11 — Table S4 Gene expression of tRNA‐coding genes. [file MPP-20-1550-s011.docx]

**Table S4.** Gene expression of tRNA-coding genes

| **Locus tag** | **Product description** | **Log2FC (Δ*dksA*/WT)** | **Log2FC (Δ*spoT***Δ***relA*/WT)** |
| --- | --- | --- | --- |
| XAC0391 | tRNA-Arg | -1.06 | 1.69 |
| XAC0489 | tRNA-Gln | 1.50 | 1.63 |
| XAC0490 | tRNA-Met | 2.29 | 1.70 |
| XAC0949 | tRNA-Gln | 2.33 | 1.31 |
| XAC0954 | tRNA-Tyr | 2.74 | 1.40 |
| XAC0955 | tRNA-Gly | 2.41 | 0.91 |
| XAC0956 | tRNA-Thr | 2.17 | 0.76 |
| XAC0958 | tRNA-Trp | 2.86 | 2.58 |
| XAC1048 | tRNA-Pro | 2.44 | 1.94 |
| XAC1049 | tRNA-Arg | 2.57 | 2.14 |
| XAC1050 | tRNA-His | 2.19 | 2.53 |
| XAC1073 | tRNA-Lys | 2.26 | 2.69 |
| XAC1076 | tRNA-Leu | 0.31 | 0.06 |
| XAC1082 | tRNA-Val | 2.04 | 2.03 |
| XAC1083 | tRNA-Asp | 2.52 | 2.95 |
| XAC1084 | tRNA-Asp | 1.20 | 1.83 |
| XAC1092 | tRNA-Ser | 2.72 | 1.63 |
| XAC1108 | tRNA-Ser | -1.66 | -1.45 |
| XAC1134 | tRNA-Val | -0.23 | 0.55 |
| XAC1257 | tRNA-Thr | 0.59 | 1.84 |
| XAC1447 | tRNA-Leu | -0.33 | -0.46 |
| XAC1565 | tRNA-Leu | -0.03 | 1.01 |
| XAC1656 | tRNA-Ser | 1.65 | 2.03 |
| XAC1744 | tRNA-Ser | 0.40 | 1.76 |
| XAC1750 | tRNA-Arg | 1.61 | 1.32 |
| XAC1751 | tRNA-Arg | 1.87 | 1.09 |
| XAC1782 | tRNA-Glu | 0.85 | 0.84 |
| XAC1809 | tRNA-Arg | 1.06 | 0.82 |
| XAC2056 | tRNA-Leu | -1.14 | 1.30 |
| XAC2058 | tRNA-Glu | 0.96 | -0.17 |
| XAC2059 | tRNA-Ala | 1.77 | 0.46 |
| XAC2060 | tRNA-Glu | 1.39 | -0.46 |
| XAC2061 | tRNA-Ala | 1.78 | -0.04 |
| XAC2094 | tRNA-Gly | 2.92 | 0.72 |
| XAC2095 | tRNA-Cys | 2.95 | 0.46 |
| XAC2096 | tRNA-Gly | 2.64 | 0.81 |
| XAC2104 | tRNA-Leu | 0.34 | 0.66 |
| XAC2339 | tRNA-Pro | -1.00 | -5.00 |
| XAC2417 | tRNA-Ala | 0.52 | 0.74 |
| XAC2560 | tRNA-Phe | 2.53 | 1.16 |
| XAC2586 | tRNA-Pro | 0.10 | -0.42 |
| XAC2624 | tRNA-Val | 1.46 | 0.50 |
| XAC2627 | tRNA-Asn | -1.00 | -0.33 |
| XAC2690 | tRNA-Met | 3.30 | 3.09 |
| XAC2705 | tRNA-Leu | 2.71 | 2.27 |
| XAC3138 | tRNA-Lys | 1.93 | 1.16 |
| XAC3299 | tRNA-Gly | 0.97 | 0.67 |
| XAC3786 | tRNA-Met | -1.07 | 0.31 |
| XAC3894 | tRNA-Ile | 2.51 | 1.01 |
| XAC3895 | tRNA-Ala | 2.57 | 1.02 |
| XAC3963 | tRNA-Ala | 7.62 | 6.65 |
| XAC4015 | tRNA-Thr | 0.45 | 1.44 |
| XAC4289 | tRNA-Ile | 2.51 | 1.01 |
| XAC4290 | tRNA-Ala | 2.57 | 1.02 |
