# Supplementary material for: Stringent response regulators (p)ppGpp and DksA positively regulate virulence and host adaptation of Xanthomonas citri
Source: Mol Plant Pathol. 2019 Oct 17;20(11):1550–65. doi: 10.1111/mpp.12865 (PMC6804348; doi:10.1111/mpp.12865)
Supplement: Supplementary file 12 — Table S5 Gene expression of ribosome protein genes. [file MPP-20-1550-s012.docx]

**Table S5.** Gene expression of ribosome protein genes

| **Gene name** | **Locus tag** | **Product description** | **Log2FC**  **(Δ*dksA*/WT)** | **Log2FC (Δ*spoT*Δ*relA*/WT)** |
| --- | --- | --- | --- | --- |
| *rplM* | XAC0487 | 50S ribosomal protein L13 | 1.41 | 0.87 |
| *rpsI* | XAC0488 | 30S ribosomal protein S9 | 1.32 | 0.98 |
| *rplY* | XAC0951 | 50S ribosomal protein L25 | 2.11 | 2.00 |
| *rplK* | XAC0961 | 50S ribosomal protein L11 | 1.55 | 1.77 |
| *rplA* | XAC0962 | 50S ribosomal protein L1 | 1.77 | 2.00 |
| *rplJ* | XAC0963 | 50S ribosomal protein L10 | 0.92 | 1.72 |
| *rplL* | XAC0964 | 50S ribosomal protein L7/L12 | 1.13 | 1.76 |
| *rpsL* | XAC0967 | 30S ribosomal protein S12 | 1.10 | 1.76 |
| *rpsG* | XAC0968 | 30S ribosomal protein S7 | 1.28 | 2.03 |
| *rpsJ* | XAC0971 | 30S ribosomal protein S10 | 1.13 | 1.95 |
| *rplC* | XAC0972 | 50S ribosomal protein L3 | 1.63 | 2.55 |
| *rplD* | XAC0973 | 50S ribosomal protein L4 | 1.53 | 2.61 |
| *rplW* | XAC0974 | 50S ribosomal protein L23 | 0.97 | 1.86 |
| *rplB* | XAC0975 | 50S ribosomal protein L2 | 1.22 | 2.14 |
| *rpsS* | XAC0976 | 30S ribosomal protein S19 | 1.13 | 2.38 |
| *rplV* | XAC0977 | 50S ribosomal protein L22 | 1.26 | 2.53 |
| *rpsC* | XAC0978 | 30S ribosomal protein S3 | 1.05 | 2.36 |
| *rplP* | XAC0979 | 50S ribosomal protein L16 | 0.67 | 1.71 |
| *rpmC* | XAC0980 | 50S ribosomal protein L29 | 0.66 | 1.69 |
| *rpsQ* | XAC0981 | 30S ribosomal protein S17 | 1.25 | 2.48 |
| *rplN* | XAC0982 | 50S ribosomal protein L14 | 0.93 | 2.19 |
| *rplX* | XAC0983 | 50S ribosomal protein L24 | 0.98 | 2.16 |
| *rplE* | XAC0984 | 50S ribosomal protein L5 | 1.01 | 2.15 |
| *rpsN* | XAC0985 | 30S ribosomal protein S14 | 1.52 | 2.84 |
| *rpsH* | XAC0986 | 30S ribosomal protein S8 | 2.08 | 1.75 |
| *rplF* | XAC0987 | 50S ribosomal protein L6 | 2.12 | 1.99 |
| *rplR* | XAC0988 | 50S ribosomal protein L18 | 2.17 | 2.55 |
| *rpsE* | XAC0989 | 30S ribosomal protein S5 | 1.88 | 2.50 |
| *rpmD* | XAC0990 | 50S ribosomal protein L30 | 1.86 | 2.34 |
| *rplO* | XAC0991 | 50S ribosomal protein L15 | 1.96 | 2.57 |
| *rpsM* | XAC0993 | 30S ribosomal protein S13 | 1.64 | 1.90 |
| *rpsK* | XAC0994 | 30S ribosomal protein S11 | 1.46 | 1.58 |
| *rpsD* | XAC0995 | 30S ribosomal protein S4 | 1.75 | 2.15 |
| *rplQ* | XAC0997 | 50S ribosomal protein L17 | 1.05 | 2.18 |
| *rpmF* | XAC1122 | 50S ribosomal protein L32 | 1.41 | -0.35 |
| *rplU* | XAC1248 | 50S ribosomal protein L21 | 1.43 | 1.31 |
| *rpmA* | XAC1249 | 50S ribosomal protein L27 | 2.11 | 1.99 |
| *rpsT* | XAC1251 | 30S ribosomal protein S20 | 2.59 | 0.83 |
| *rpsP* | XAC1292 | 30S ribosomal protein S16 | 2.69 | 2.58 |
| *rplS* | XAC1295 | 50S ribosomal protein L19 | 2.63 | 2.19 |
| *rpsB* | XAC1422 | 30S ribosomal protein S2 | 1.80 | 0.60 |
| *rpsF* | XAC1620 | 30S ribosomal protein S6 | 2.48 | 2.37 |
| *rpsR* | XAC1621 | 30S ribosomal protein S18 | 2.39 | 2.26 |
| *rplI* | XAC1622 | 50S ribosomal protein L9 | 2.99 | 3.19 |
| *rpsA* | XAC2298 | 30S ribosomal protein S1 | 1.79 | 2.23 |
| *rpmJ* | XAC2300 | 50S ribosomal protein L36 | 3.59 | 2.26 |
| *rplT* | XAC2591 | 50S ribosomal protein L20 | 2.15 | 1.67 |
| *rpmI* | XAC2592 | 50S ribosomal protein L35 | 2.19 | 0.82 |
| *rpsO* | XAC2684 | 30S ribosomal protein S15 | 1.91 | 1.44 |
| *-* | XAC2827 | 30S ribosomal protein S31 | 2.36 | 2.12 |
| *rpmE* | XAC3389 | 50S ribosomal protein L31 | 2.83 | 1.00 |
| *rpsU* | XAC3872 | 30S ribosomal protein S21 | 3.39 | 0.53 |
| *rpmG* | XAC4158 | 50S ribosomal protein L33 | 2.55 | 0.93 |
| *rpmB* | XAC4159 | 50S ribosomal protein L28 | 2.66 | 0.96 |
| *rpmH* | XAC4374 | 50S ribosomal protein L34 | 2.00 | 1.51 |
