# Supplementary material for: Stringent response regulators (p)ppGpp and DksA positively regulate virulence and host adaptation of Xanthomonas citri
Source: Mol Plant Pathol. 2019 Oct 17;20(11):1550–65. doi: 10.1111/mpp.12865 (PMC6804348; doi:10.1111/mpp.12865)
Supplement: Supplementary file 13 — Table S6 Gene expression profile of T3SS‐ and T2SS‐related genes in Xcc. [file MPP-20-1550-s013.docx]

**Table S6.** Gene expression profile of T3SS- and T2SS-related genes in Xcc

|  | **Gene** | **Locus tag** | **Log2FC (Δ*dksA*/WT)** | **Log2FC (Δ*spoT***Δ***relA*/WT)** |
| --- | --- | --- | --- | --- |
|  |  |  |  |  |
| **Key regulatory genes** | *hrpG* | XAC1265 | -1.90 | -1.13 |
|  | *hrpX* | XAC1266 | -1.90 | -2.98 |
|  |  |  |  |  |
| **hrp/hrc** | *hrcS* | XAC0401 | -3.17 | -4.98 |
|  | *hrcU* | XAC0406 | -3.03 | -4.61 |
|  | *hpa1* | XAC0416 | -3.21 | -4.38 |
|  | *hrpB5* | XAC0411 | -2.78 | -4.2 |
|  | *hrcR* | XAC0402 | -3.00 | -3.58 |
|  | *hpaP* | XAC0404 | -2.78 | -3.26 |
|  | *hrcT* | XAC0414 | -3.17 | -3.17 |
|  | *hrpB7* | XAC0413 | -3.04 | -3.04 |
|  | *hrcN* | XAC0412 | -3.17 | -2.95 |
|  | *hrcQ* | XAC0403 | -2.66 | -2.66 |
|  | *hpa2* | XAC0417 | -3.58 | -2.58 |
|  | *hrcJ* | XAC0409 | -2.66 | -2.44 |
|  | *hrpD5* | XAC0399 | -2.42 | -2.42 |
|  | *hpaA* | XAC0400 | -2.40 | -2.4 |
|  | *hrpB4* | XAC0410 | -2.69 | -2.24 |
|  | *hrpE* | XAC0397 | -1.44 | -2.21 |
|  | *hpaB* | XAC0396 | -2.49 | -2.17 |
|  | *hrpD6* | XAC0398 | -1.86 | -1.93 |
|  | *hrpF* | XAC0394 | -2.58 | -1.58 |
|  | *hrcV* | XAC0405 | -2.91 | -1.58 |
|  | *hrpB2* | XAC0408 | -2.07 | -1.39 |
|  | *hrpB1* | XAC0407 | -2.45 | -1.31 |
|  | *hrcC* | XAC0415 | -2.74 | -0.51 |
|  | *hpaF* | XAC0393 | -1.50 | -0.50 |
|  |  |  |  |  |
| ***effector genes***  ***effector genes*** | *avrBs2* | XAC0076 | -1.72 | -2.72 |
|  | *xopR* | XAC0277 | -0.86 | -1.16 |
|  | *XopE1* | XAC0286 | -2.66 | -1.17 |
|  | *xopS* | XAC0315 | -1.84 | -2.32 |
|  | *xopM* | XAC0418 | -3.00 | -0.04 |
|  | *xopX* | XAC0543 | -2.47 | -1.93 |
|  | *xopV* | XAC0601 | -2.00 | 0.29 |
|  | *xopI* | XAC0754 | -2.77 | -3.04 |
|  | *xopAU* | XAC1171 | -2.32 | -0.94 |
|  | *xopAV* | XAC1172 | -2.48 | -1.07 |
|  | *xopP* | XAC1208 | -2.27 | -0.77 |
|  | *xopAZ* | XAC1358 | -0.57 | 1.22 |
|  | *xopZ* | XAC2009 | -2.26 | -2.58 |
|  | *xopF* | XAC2785 | -2.04 | -4.04 |
|  | *xopN* | XAC2786 | -2.86 | -2.54 |
|  | *xopAW* | XAC2949 | -0.57 | 0.39 |
|  | *xopAP* | XAC2990 | -2.66 | -1.08 |
|  | *xopK* | XAC3085 | -2.26 | -3.43 |
|  | *xopL* | XAC3090 | -2.50 | -1.08 |
|  | *XopE3* | XAC3224 | -1.28 | 0.08 |
|  | *xopAI* | XAC3230 | -1.49 | -1.22 |
|  | *xopAK* | XAC3666 | -1.71 | -1.17 |
|  | *xopAD* | XAC4213 | -1.93 | -1.66 |
|  | *xopQ* | XAC4333 | -2.09 | -1.50 |
|  | *pthA1* | XACa0022 | -0.82 | -0.79 |
|  | *pthA2* | XACa0039 | -0.96 | -0.76 |
|  | *XopE2* | XACb0011 | -0.61 | 0.33 |
|  | *pthA3* | XACb0015 | -0.79 | -0.57 |
|  | *pthA4* | XACb0065 | -0.73 | -0.71 |
|  |  |  |  |  |
| **T2SS (*xcs*)** | *xcsC* | XAC0694 | -2.58 | -3.91 |
|  | *xcsD* | XAC0695 | -2.91 | -2.58 |
|  | *xcsE* | XAC0696 | -3.00 | -2.19 |
|  | *xcsF* | XAC0697 | -3.07 | -4.39 |
|  | *xcsG* | XAC0698 | -2.94 | -1.72 |
|  | *xcsH* | XAC0699 | -3.25 | -1.25 |
|  | *xcsI* | XAC0700 | -3.19 | -3.00 |
|  | *xcsJ* | XAC0701 | -3.10 | -2.84 |
|  | *xcsK* | XAC0702 | -2.89 | -1.75 |
|  | *xcsL* | XAC0703 | -2.85 | -2.36 |
|  | *xcsM* | XAC0704 | -1.77 | 0.00 |
|  | *xcsN* | XAC0705 | -2.39 | -1.22 |
|  |  |  |  |  |
| **degradative enzymes** | *-* | XAC3547 | -2.91 | -1.58 |
|  | *-* | XAC0933 | -1.93 | -2.25 |
|  | *xynB* | XAC4252 | -2.58 | -1.58 |
|  | *xynB* | XAC4254 | -2.46 | -1.76 |
|  | *xynB* | XAC0160 | -2.46 | -3.20 |

Note: XAC0393, which belongs to the *hrp/hrc* cluster, is also considered to be an effector.
