# Supplementary material for: Stringent response regulators (p)ppGpp and DksA positively regulate virulence and host adaptation of Xanthomonas citri
Source: Mol Plant Pathol. 2019 Oct 17;20(11):1550–65. doi: 10.1111/mpp.12865 (PMC6804348; doi:10.1111/mpp.12865)
Supplement: Supplementary file 14 — Table S7 Gene expression profile for putative type 4 pilus biogenesis and regulation genes of Xcc. [file MPP-20-1550-s014.docx]

| **Gene** | **Locus tag** | **Product description** | **Log_2_ FC (Δ*dksA*/WT)** | **Log_2_ FC (Δ*spoT/relA*/WT)** |
| --- | --- | --- | --- | --- |
| *pilZ* | XAC1133 | type IV pilus regulatory protein | 1.83 | 0.21 |
| *pilF* | XAC2017 | fimbrial biogenesis protein | 1.61 | 0.88 |
| *fimX* | XAC2398 | type IV pilus regulatory protein | 8.31 | -0.14 |
| *pilE* | XAC2664 | prepilin-like protein | 5.87 | -1.97 |
| *pilY1* | XAC2665 | PilY1 protein | 4.38 | -2.24 |
| *pilX* | XAC2666 | prepilin-like protein | 5.37 | -1.52 |
| *PliW* | XAC2667 | prepilin-like protein | 8.56 | -0.43 |
| *pilV* | XAC2668 | prepilin-like protein | 11.22 | -0.03 |
| *fimT* | XAC2669 | prepilin-like protein | 10.77 | 0.55 |
| *pilU* | XAC2923 | pilus biogenesis protein | 4.60 | -0.49 |
| *pilT* | XAC2924 | pilus biogenesis protein | 4.04 | 0.46 |
| *pilL* | XAC3098 | PilL protein | 4.36 | -0.97 |
| *pilJ* | XAC3099 | pilus biogenesis protein | 6.39 | -0.73 |
| *pilI* | XAC3100 | pilus biogenesis protein | 5.82 | 0.05 |
| *pilH* | XAC3101 | pilus biogenesis protein | 6.04 | 0.63 |
| *pilG* | XAC3102 | pilus biogenesis protein | 3.75 | 0.73 |
| *pilS* | XAC3237 | two-component system regulatory protein | 0.63 | -0.57 |
| *pilR* | XAC3238 | two-component system regulatory protein | 0.86 | -1.15 |
| *pilB* | XAC3239 | pilus biogenesis protein | 1.15 | -0.71 |
| *fimA* | XAC3240 | prepilin | 1.79 | -1.02 |
| *fimA* | XAC3241 | prepilin | 9.15 | -0.16 |
| *pilC* | XAC3242 | pilus biogenesis protein | 2.94 | 0.01 |
| *pilD* | XAC3243 | prepilin leader peptidase | 2.34 | 0.80 |
| *pilQ* | XAC3381 | pilus biogenesis protein | 3.58 | -1.05 |
| *pilP* | XAC3382 | pilus biogenesis protein | 3.10 | -1.51 |
| *pilO* | XAC3383 | pilus biogenesis protein | 4.43 | -0.51 |
| *pilN* | XAC3384 | pilus biogenesis protein | 3.46 | -0.74 |
| *pilM* | XAC3385 | pilus biogenesis protein | 6.72 | -0.43 |

**Table S7.** Gene expression profile for putative type 4 pilus biogenesis and regulation genes of Xcc
