# Supplementary material for: Stringent response regulators (p)ppGpp and DksA positively regulate virulence and host adaptation of Xanthomonas citri
Source: Mol Plant Pathol. 2019 Oct 17;20(11):1550–65. doi: 10.1111/mpp.12865 (PMC6804348; doi:10.1111/mpp.12865)
Supplement: Supplementary file 15 — Table S8 Gene expression level of TBDT genes of Xcc. [file MPP-20-1550-s015.docx]

**Table S8.** Gene expression level of TonB-dependent transporter genes of Xcc

| **Locus tag** | **Gene name** | **Product description** | **log2FC (Δ*dksA*/WT)** | **log2FC (Δ*spoT***Δ***relA*/WT)** |
| --- | --- | --- | --- | --- |
| XAC0074 | *cirA* | TonB-dependent receptor | -1.91 | -3.17 |
| XAC0144 | *iroN* | TonB-dependent receptor | -1.04 | 0.00 |
| XAC0653 | *fepA* | TonB-dependent receptor | -1.40 | -2.54 |
| XAC0690 | *fecA* | TonB-dependent receptor | -1.85 | -0.71 |
| XAC0693 | *fecA* | TonB-dependent receptor | -3.25 | -3.25 |
| XAC0706 | *iroN* | TonB-dependent receptor | -1.91 | -0.45 |
| XAC0716 | *fyuA* | TonB-dependent receptor | -1.16 | -0.95 |
| XAC0811 | *cirA* | TonB-dependent receptor | -2.81 | -0.35 |
| XAC0852 | *-* | TonB-dependent receptor | -2.81 | -3.39 |
| XAC1023 | *fecA* | TonB-dependent receptor | -2.58 | -2.58 |
| XAC1143 | *fyuA* | TonB-dependent receptor | 3.08 | 0.92 |
| XAC1146 | *fecA* | TonB-dependent receptor | -2.70 | -1.38 |
| XAC1276 | *fyuA* | TonB-dependent receptor | -0.10 | -0.86 |
| XAC1310 | *btuB* | TonB-dependent receptor | -1.65 | -0.07 |
| XAC1768 | *fhuA* | TonB-dependent receptor | -2.00 | -1.74 |
| XAC1769 | *cirA* | TonB-dependent receptor | -1.91 | -1.32 |
| XAC1910 | *cirA* | TonB-dependent receptor | -0.30 | -1.69 |
| XAC2024 | *cirA* | TonB-dependent receptor | -2.77 | -3.09 |
| XAC2193 | *cirA* | TonB-dependent receptor | -2.00 | -2.17 |
| XAC2520 | *cirA* | TonB-dependent receptor | -2.75 | -2.43 |
| XAC2531 | *btuB* | TonB-dependent receptor | -2.91 | -2.32 |
| XAC2535 | *btuB* | TonB-dependent receptor | -2.87 | -2.46 |
| XAC2600 | *btuB* | TonB-dependent receptor | -2.30 | -2.98 |
| XAC2742 | *btuB* | TonB-dependent receptor | -0.93 | -2.25 |
| XAC2830 | *fhuA* | TonB-dependent receptor | -1.72 | -3.52 |
| XAC2941 | *fhuA* | TonB-dependent receptor | 3.23 | -0.29 |
| XAC2998 | *fecA* | TonB-dependent receptor | -2.74 | -2.74 |
| XAC3050 | *btuB* | TonB-dependent receptor | 3.00 | -1.24 |
| XAC3071 | *iroN* | TonB-dependent receptor | -2.38 | -2.10 |
| XAC3077 | *cirA* | TonB-dependent receptor | -3.17 | -3.17 |
| XAC3121 | *fepA* | TonB-dependent receptor | -0.21 | -1.14 |
| XAC3158 | *fhuA* | TonB-dependent receptor | -2.50 | -2.50 |
| XAC3201 | *fyuA* | TonB-dependent receptor | 1.42 | -3.22 |
| XAC3311 | *iroN* | TonB-dependent receptor | -2.52 | -2.20 |
| XAC3334 | *fecA* | TonB-dependent receptor | -2.22 | 0.42 |
| XAC3366 | *cirA* | TonB-dependent receptor | -1.74 | 0.26 |
| XAC3427 | *fhuA* | TonB-dependent receptor | -1.64 | -0.56 |
| XAC3444 | *btuB* | TonB-dependent receptor | -0.17 | 0.49 |
| XAC3448 | *btuB* | TonB-dependent receptor | -2.09 | -0.92 |
| XAC3489 | *fyuA* | TonB-dependent receptor | -2.20 | -1.94 |
| XAC3560 | *btuB* | TonB-dependent receptor | 0.68 | -2.83 |
| XAC3613 | *btuB* | TonB-dependent receptor | 2.13 | -0.94 |
| XAC4048 | *iroN* | TonB-dependent receptor | -0.78 | 0.18 |
| XAC4062 | *fhuA* | TonB-dependent receptor | -2.10 | -2.58 |
| XAC4256 | *cirA* | TonB-dependent receptor | -3.25 | -4.25 |
| XAC4368 | *fecA* | TonB-dependent receptor | -2.58 | -2.58 |
