# Supplementary material for: Stringent response regulators (p)ppGpp and DksA positively regulate virulence and host adaptation of Xanthomonas citri
Source: Mol Plant Pathol. 2019 Oct 17;20(11):1550–65. doi: 10.1111/mpp.12865 (PMC6804348; doi:10.1111/mpp.12865)
Supplement: Supplementary file 16 — Table S9 Differentially regulated genes by DksA and ppGpp. [file MPP-20-1550-s016.docx]

| Name | Locus tag | Product | Δ*dksA*† | Δ*spoT/relA*‡ |
| --- | --- | --- | --- | --- |
| *pilY1* | XAC2665 | PilY1 protein | 2.13 | -2.24 |
| *cysJ* | XAC3330 | NADPH-sulfite reductase flavoprotein subunit | 3.44 | -2.94 |
| *cysG* | XAC3340 | siroheme synthase | 3.34 | -4.52 |
|  | XAC3156 | hypothetical protein | 2.12 | -2.24 |
| *nrtCD* | XAC0828 | ABC transporter ATP-binding protein | 2.18 | -2.22 |
| *-* | XAC3750 | hypothetical protein | -2.63 | +3.92 |
| *-* | XAC0824 | hypothetical protein | 2.16 | -2.97 |
|  | XAC0825 | hypothetical protein | 2.76 | -3.39 |
| *mphE* | XAC3175 | 4-hydroxy-2-oxovalerate aldolase | 3.37 | -3.00 |
| *fecA* | XAC3176 | citrate-dependent iron transporter | 4.50 | -2.81 |
| *-* | XAC3177 | hypothetical protein | 4.96 | -2.63 |
| *-* | XAC3178 | hypothetical protein | 3.38 | -5.39 |
| *yceE* | XAC3179 | transporter | 2.09 | -4.58 |
| *iucA* | XAC3180 | iron transporter | 2.93 | -3.51 |
| *lysA* | XAC3181 | diaminopimelate decarboxylase | 2.82 | -3.39 |
|  | XAC3749 | hypothetical protein | -2.97 | 3.84 |

**Table S9.** Differentially regulated genes by DksA and ppGpp

† values represent log2FC(Δ*dksA*/WT); ‡ values represent log2FC(Δ*spoT*Δ*relA*/WT)
