# Supplementary material for: Stringent response regulators (p)ppGpp and DksA positively regulate virulence and host adaptation of Xanthomonas citri
Source: Mol Plant Pathol. 2019 Oct 17;20(11):1550–65. doi: 10.1111/mpp.12865 (PMC6804348; doi:10.1111/mpp.12865)
Supplement: Supplementary file 17 — Table S10 Strains and plasmids used in this study. [file MPP-20-1550-s017.docx]

| **Types** | **Relevant characteristic** | **Reference or source** |
| --- | --- | --- |
| **strains** |  |  |
| *Escherichia coli* |  |  |
| HST08 | F–, endA1, supE44, thi-1, recA1, relA1, gyrA96, phoA, Φ80d lacZΔ M15, Δ (lacZYA - argF) U169, Δ (mrr - hsdRMS - mcrBC), ΔmcrA, λ– | Clontech Laboratories Inc, Mountain View, CA |
|  |  |  |
| S-17-1 λpir | RK2 tra regulon, pir, host for pir-dependent plasmid pOK1 | (1) |
| *Xanthomonas citri* subsp. *citri* |  |  |
| Xcc306 | Syn. *X. axonopodis* pv. *citri* strain 306; wild type; Rif^r^ | (2) |
| Xcc306Δ*dksA* | *dksA* gene deletion strain in the background of Xcc306; Rif^r^ | This study |
| Xcc306Δ*relA* | *relA* gene deletion strain in the background of Xcc306; Rif^r^ | This study |
| Xcc306Δ*spoT/relA* | both *spoT* and *relA* gene deletion strain in the background of Xcc306; Rif^r^ | This study |
| **plasmids** |  |  |
| pBBR1MCS-2 | Broad host expression vector. Kn^R^ | (3) |
| pBBR1MCS-2:*hrpG* | pBBR1MCS-2 derivative for expression of HrpG fused to HA tag. Kn^R^ | (5) |
| pBBR1MCS-2:*dksA* | pBBR1MCS-2 derivative for expression of DksA (XAC2358). Kn^R^ | This study |
| pBBR1MCS-2:*spoT* | pBBR1MCS-2 derivative for expression of SpoT (XAC3393). Kn^R^ | This study |
| pBBR1MCS-5 | Broad host expression vector. Gn^R^ | (3) |
| pGUS | pBBR1MCS-5 derivative containing *gus* gene followed by T7 terminator cloned in reverse orientation of lac promoter. | (5) |
| pGUS *phrpG* | pGUS derivative. The 699 bp upstream region of XAC1265 was ckobed upstream to *gus* reporter. Gn^R^ |  |
| pGUS *pgyrA* | pGUS derivative. The 189 bp upstream region of XAC1631 was cloned upstream to gus. Gn^R^ | (5) |
| pGUS *phrpX* | pGUS derivative. The 474 bp upstream region of XAC1266 was cloned upstream to gus. Gn^R^ | (5) |
| pGUS *phrpF* | pGUS derivative. The 831 bp upstream region of XAC0394 was cloned upstream to gus. Gn^R^ | (5) |
| pGUS *pxopAU* | pGUS derivative. The 1000 bp upstream region of XAC1171 was cloned upstream to gus. Gn^R^ | (5) |
| pGUS *pmphE* | pGUS derivative. The 240 bp upstream region of XAC1171 was cloned upstream to gus. Gn^R^ | This study |
| pGUS *psuxA* | pGUS derivative. The 204 bp upstream region of XAC1171 was cloned upstream to gus. Gn^R^ | This study |
| pOK1 | sacB sacQ mobRK2 oriR6K, Suicide vector. Sp^R^ | (4) |
| pOK1:*dksA* | pOK1 derivative contacting the flanking regions of *dksA* (XAC2358). Sp^R^ | This study |
| pOK1:*relA* | pOK1 derivative contacting the flanking regions of *relA* (XAC3113). Sp^R^ | This study |
| pOK1:*spoT* | pOK1 derivative contacting the flanking regions of *spoT* (XAC3393). Sp^R^ | This study |

**Table S10.** Strains and plasmids used in this study

*Kn^R^, Gn^R^, Sp^R^ and Rif^R^ indicate resistance to kanamycin, gentamicin, rifampicin, respectively.

**Reference:**

(1) Simon R, Priefer U, Pühler A. 1983. A broad host range mobilization system for in vivo genetic engineering: Transposon mutagenesis in gram negative bacteria. Bio/Technology 1:784–791.

(2) Da Silva ACR, Ferro JA, Reinach FC, Farah CS, Furlan LR, Quaggio RB, Monteiro-Vitorello CB, Van Sluys MA, Almeida NF, Alves LMC, Do Amaral AM, Bertolini MC, Camargo LEA, Camarotte G, Cannavan F, Cardozo J, Chambergo F, Ciapina LP, Cicarelli RMB, Coutinho LL, Cursino-Santos JR, El-Dorry H, Faria JB, Ferreira AJS, Ferreira RCC, Ferro MIT, Formighieri EF, Franco MC, Greggio CC, Gruber A, Katsuyama AM, Kishi LT, Leite RP, Lemos EGM, Lemos MVF, Locali EC, Machado MA, Madeira AMBN, Martinez-Rossi NM, Martins EC, Meidanis J, Menck CFM, Miyaki CY, Moon DH, Moreira LM, Novo MTM, Okura VK, Oliveira MC, Oliveira VR, Pereira HA, Rossi A, Sena JAD, Silva C, De Souza RF, Spinola LAF, Takita MA, Tamura RE, Teixeira EC, Tezza RID, Trindade dos Santos M, Truffi D, Tsai SM, White FF, Setubal JC, Kitajima JP. 2002. Comparison of the genomes of two Xanthomonas pathogens with differing host specificities. Nature 417:459–463.

(3) Kovach ME, Elzer PH, Steven Hill D, Robertson GT, Farris MA, Roop RM, Peterson KM. 1995. Four new derivatives of the broad-host-range cloning vector pBBR1MCS, carrying different antibiotic-resistance cassettes. Gene 166:175–176.

(4) Huguet E, Hahn K, Wengelnik K, Bonas U. 1998. hpaA mutants of Xanthomonas campestris pv. vesicatoria are affected in pathogenicity but retain the ability to induce host-specific hypersensitive reaction. Mol Microbiol 29:1379–1390.

(5) Teper, D., Zhang, Y. and Wang, N. (2019) TfmR, a novel TetR‐family transcriptional regulator, modulates the virulence of Xanthomonas citri in response to fatty acids. Mol. Plant Pathol., mpp.12786.
