# Supplementary material for: Stringent response regulators (p)ppGpp and DksA positively regulate virulence and host adaptation of Xanthomonas citri
Source: Mol Plant Pathol. 2019 Oct 17;20(11):1550–65. doi: 10.1111/mpp.12865 (PMC6804348; doi:10.1111/mpp.12865)
Supplement: Supplementary file 18 — Table S11 Primer sequence used in this study. [file MPP-20-1550-s018.docx]

**Table S11.** Primer sequence used in this study

| **Primer name** | **Primer sequence** |
| --- | --- |
| for *hrp* genes |  |
| hrpF-RTF | AAGCAAGCAAAGAACTACACACAG |
| hrpF-RTR | GAAGGTATCCTGATTCAGATCGTC |
| hrpX-RTF | AGCGATCTCTGCGTTGTCCTAC |
| hrpX-RTR | ATACGCATCTTCGGCCTCTTCCTGA |
| hrpG-RTF | ATCGTGCTTGGACGTTTCGATTGC |
| hrpG-RTR | ATTGAAAGGCAGCGCAAGGACTTC |
| xopAU-RTF | GATCCCACTGACACTGTACGAC |
| xopAU-RTR | TGATCATTTGATACTCCTGGAAGA |
| xopN-RTF | AACAACAAGCTCAACCTCCTGTA |
| xopN-RTR | GTAGTTTGTCGGCCTTGATGTC |
| gyrA-RTF | GTCAAGGAAAAGAAGCTCGAAG |
| gyrA-RTR | GCTGATACAGGTTGTTGAGCAC |
| for deletion mutatant |  |
| FdskABamHI | AAA GGATCCCGATCTTCCGCAGCATCGC |
| RdskA | CGTGGCCGTGACGTCCAACGCCTGGCTCGCCGAAAGA |
| FdskA | TCTT TCGGCGAGCCAGGCGTTGGACGTCACG GCCACG |
| RdskAXbaI | CCCC TCTAGAAACAGCTTGGTGCAAAACGG |
| FrelABamHI | AAAGGATCCATCGAGAGCAGTCCGACGATG |
| RrelA | CAGGTAAGCGGCGTCCTCAG GGTAACGTGCGCGTCACCT |
| FrelA | AGGTGACGCGCACGTTACC CTGAGGACGCCGCTTACCTG |
| RrelAXbaI | AAATCTAGATGCGTTGGTAGAGCGGCAAC |
| FspoTApaI | AAA GGGCCCGGTAACTGGGGTGCACGA |
| RspoT | ATGAACCCAG GCCCCACTGG CAACAGTCGA GCAAATCCGC |
| FspoT | GCGGATTTGCTCGACTGTTGCCAGTGGGGCCTGGGTTCAT |
| RspoTXbaI | CCCC TCTAGA CGAGACCTTCGATACGGCG |
| for complementation |  |
| FdksA(CXbaI) | CCC TCTAGACATCGCGTAGTCCGAACAG |
| RdksA(CBamHI) | AAAA GGATCCCGCTAATTCGACGTATCAGG |
| FspoT(CXbaI) | CCC TCTAGATGGAAGTCGTGAACAACCGT |
| RspoT(CBamHI) | AAAA GGATCCAAAGGGTTGCCGCAGTTTAG |
| for GUS assay |  |
| mphE-gusf(XbaI) | AAAATCTAGACGATAAACGCCTTGTTTCTCA |
| mphE-gusr(EcoR1) | AAAAGAATTCCATGGAAGAGGCCGCCAAGGA |
| SuxA-gusf(XbaI) | AAAATCTAGAACTGTACCTTGCCGCCGTTG |
| SuxA-gusr(EcoR1) | AAAAGAATTCCATTGCGATTCTCTCTCTCAC |
| hrpG-gusf(BamHI) | CCCGGATCCTCACTCTGTCAAACATCCATGTGATG |
| hrpG-gusf(EcoRI) | AAAGAATTCGGGAGAGTGGTCGTTCATTTAGG |
| for validation of RNA-seq data |  |
| for Δ*dksA* |  |
| XAC0637F | AGAACCCCAACATCGTTCAC |
| XAC0637R | ATTGCCCTTCATGACCGTAT |
| XAC0823F | ATGCCAACCGTAACTTCGTC |
| XAC0823R | CGCTCAGATAATCCGAAGGA |
| XAC1008F | ACGACCGTCTATCTGGAAGC |
| XAC1008R | AGTGACCACCTGGCACATC |
| XAC1151F | CTGCAGAACGAGATCAAGCA |
| XAC1151R | GCGTACAGCACGAAGTGATT |
| XAC1211F | ATTCACCCGTTTTTCCACTG |
| XAC1211R | AGATCCCAGTTGCCTTCCTT |
| XAC1523F | GAGGAGCTGAAGAAGGCGTA |
| XAC1523R | TTCGTAGGCTTCCTTGCATT |
| XAC1735F | CGTGTCGGTGTATCTGGTCA |
| XAC1735R | GCGTGCTTGTACACCATCTG |
| XAC1904F | TACAAGTTCACCCCCATGCT |
| XAC1904R | GCGATCAGCTGTTCTGGATT |
| XAC2013F | CGGAAGTGGTCGAGATCCTA |
| XAC2013R | CAGATGGAAGGCGCTGTAGT |
| XAC2039F | GTCCACCATCAAGGAACTCG |
| XAC2039R | GCACCCATGAACTGACGATA |
| XAC2528F | GGAATACACCTCGCTGCTGT |
| XAC2528R | GCCTGGTCCATGATGAAAAC |
| XAC2668F | TTCAGTCGTACGCGATTCTG |
| XAC2668R | GATCCAATCTCGTCGGTCAT |
| XAC2924F | AAGGTGGCGCAGATGTATTC |
| XAC2924R | TCGAATATCCGCTTGTCCTT |
| XAC3042F | CAACCGTTGCTGGATTTCTT |
| XAC3042R | TTGGCCTGGATCTTGAAAAC |
| XAC3195F | CCTCCTGGCTATGTGGGTTA |
| XAC3195R | GGCATCGTCCTTTTCCTTCT |
| XAC3242F | CTGATGCTGTTCGCACTTGT |
| XAC3242R | GACCAATGATCGGCACTTTT |
| XAC3331F | CGCAAATTCAAGATCGGTTT |
| XAC3331R | GACGTTGTAGCCCAGCAACT |
| XAC3370F | CGACTACAAGCGTTCGTTCA |
| XAC3370R | GGTCTTTTCGTGGCTCACAT |
| XAC3535F | GCATCCGTTCTTCCTGTCC |
| XAC3535R | CAACGTCGCCATCTTGAAGT |
| XAC3543F | TGTTCCTGCTCGGTTATGTG |
| XAC3543R | GATCAGCACGATCCACCAGT |
| for Δ*spoT*Δ*relA* |  |
| XAC0001F | AAGGCGATGGACCAGTTCAA |
| XAC0001R | GCGTTGAAGGTGTGGAAGAA |
| XAC0108F | AGCACGAATACACGCATCTG |
| XAC0108R | CGGCAGGTTGGTTAGATCCT |
| XAC0334F | TTCGATTTCATCGATCAGGAC |
| XAC0334R | GTGCCTGGAAGAAGCTGAAA |
| XAC0952F | GGCATTGGACAGCAAGTTGT |
| XAC0952R | GCCGCTGAGATTCATGAAGG |
| XAC1521F | CAAGTTCGCCAACGAGAAG |
| XAC1521R | TTGCTTGTAGGTCATGTCCAG |
| XAC1976F | ATGATCAACACGCTGCAGTC |
| XAC1976R | GGGTGACTTCGTTGGATTCG |
| XAC1977F | AGCAAGCTGGTCGGTTTTAC |
| XAC1977R | ACGGTGGTGAGTTTGGAAGA |
| XAC2414F | GGCACCAAGGAAGACTTCAA |
| XAC2414R | ACGGTGCCTCACTAATGTCC |
| XAC2599F | CCGTACCTATCCCAACTGGA |
| XAC2599R | GGGTGAACACCAGGTTGACT |
| XAC2743F | TCGACCAGCAGGAAACCTAT |
| XAC2743R | AGTGACTTCGGTTCCCACAC |
| XAC3054F | TTCGACAAGCGCTACATCAC |
| XAC3054R | GTGATGCCGATGTCCTTCTT |
| XAC3195F | CCTCCTGGCTATGTGGGTTA |
| XAC3195R | TGCAGCAGGATGTTGAAGAC |
| XAC4055F | GTACTACACCACCAGCGAAG |
| XAC4055R | GGATAACGTGCGGCATAGTG |
| XAC4056F | CAAGGAGAGCTTGGCGAAC |
| XAC4056R | GCTCGATCGATTCCCCAAAC |
| XAC4074F | CCTACGAAGACTTCATGCGC |
| XAC4074R | CGCAAAGCCGGAATAGAAGT |
| XACb0054F | TCAACATTTCGCCGTTCTCG |
| XACb0054R | TTGCTGTTGACCATGTTCGG |

Short underline indicates the restriction enzyme cutting site; long underline indicates the complemented primers for generating deletion mutants.
